# Comparative study of quadratic regression, Lasso, random forest, and multilayer perceptron for modeling optimal solid density in offset printing

**DOI:** 10.1371/journal.pone.0361203

**Published:** 2026-10-21

**Authors:** Bingzhong Qiu, Liangfei Li, Xiaoli Liu, Xiaolei Sheng, Yujun Jiang, Xiaomei Liu, Ying-Leh Ling

**Affiliations:** 1 General Education Department, Qilu Medical University, Zibo, Shandong, China; 2 College of Vocational Education, Tianjin University of Technology and Education, Tianjin, China; 3 Academic Affairs Office, Zibo Polytechnical Vocational School, Zibo, Shandong, China; 4 Department of Mathematics, Science and Computer, Politeknik Kuching Sarawak, Sarawak, Malaysia; University of Donja Gorica, MONTENEGRO

## Abstract

This study compares four modeling methods, namely quadratic regression (QR), Lasso, random forest (RF), and multilayer perceptron (MLP), to determine the optimal solid density for four-color offset printing. Based on 110 valid samples for each ink color, random 5-fold cross-validation (CV) and paired *t*-tests were employed to evaluate model predictive accuracy (${\text{R}}^{\text{2}}$, RMSE) and the stability of the estimated optimal solid density (CV%). Under random 5-fold CV, non-parametric approaches (RF and MLP) outperformed parametric counterparts (QR and Lasso) for black, cyan, and magenta inks, and MLP yielded the optimal performance for yellow ink (${\text{R}}^{\text{2}}$ = 0.9770). Pairwise statistical tests showed no significant differences between RF and MLP for black, cyan, and magenta inks (*p* > 0.05), while MLP was statistically superior for yellow ink under random 5-fold CV (*p* = 0.0296). However, this ranking cannot be generalized to blocked cross‑validation (blocked‑CV). Two blocked‑CV strategies, temporal-block and spatial-block CV, were further implemented to mitigate data leakage arising from high lag-1 autocorrelation (0.96–0.98) in sequentially collected process data. Under blocked-CV, mean ${\text{R}}^{\text{2}}$ values were negative across most models and ink colors, limiting the interpretability of inter-model rankings. While surfaces fitted under random 5-fold CV revealed nonlinear features including broad high-quality plateaus for cyan and magenta inks and multi-peak patterns for yellow ink, such features require cautious physical interpretation given validation-strategy dependence. Despite substantial drops in predictive performance metrics under blocked-CV, the estimated optimal solid densities were insensitive to the fold structure, with deviations ranging from 0.0034 to 0.0152 across all four ink colors. The recommended optimal solid densities are 1.67 (RF) for black ink, 1.44 (RF) for cyan ink, 1.42 (RF) for magenta ink, and 0.99 (MLP) for yellow ink. This study shows that although predictive performance metrics are highly sensitive to the validation strategies, the estimated optimal solid densities are insensitive to the fold structure examined here. This consistency is dataset‑specific and does not by itself establish out‑of‑sample validity under different printing conditions. For offset printing process optimization, the stability of the estimated optimal solid density offers greater practical engineering value than raw predictive performance or model ranking consistency.

## 1 Introduction

### 1.1 Research background

In the domain of packaging and publishing, color reproduction quality in offset printing serves as a critical indicator of the value of printed material. Solid density is a pivotal parameter in printing process control. This key parameter exerts a direct influence on the color saturation and hue accuracy of printed products. Additionally, an indirect effect on tonal gradation and relative contrast is produced through dot gain [1]. The prevailing international standard, ISO 12647−2, stipulates recommended ranges for solid densities in four-color printing. However, the efficacy of such broad intervals, based on statistical experience, is questionable in meeting the precise and customized demands of process parameter optimization required by modern precision printing [2].

In practical printing environments, enterprises must determine the optimal solid density based on actual printing conditions within the standard recommended range. Furthermore, it is imperative to compress the control tolerance to within ±0.05 units to ensure optimal balance between color performance, drying efficiency, and process stability. At present, methodologies for ascertaining the optimal solid density principally comprise two categories. Firstly, trial‑and‑error approaches that depend on the subjective assessment of operators have been shown to be inefficient and incapable of ensuring consistent printing quality. Secondly, data-driven modeling methods, such as QR, Lasso, RF, and MLP, have been employed [3]. However, extant research has focused predominantly on improving the fitting accuracy of individual models, lacking systematic comparison and statistical evaluation of parametric and non-parametric models in the critical task of “optimal solution localization” [4]. This theoretical discrepancy directly results in arbitrary method selection in production practice, thereby affecting the stability of print quality and the scientific nature of process optimization.

Therefore, a comparison of the effectiveness, robustness, and engineering applicability of QR, Lasso, RF, and MLP in determining the optimal solid density for offset printing is of significant theoretical value. Furthermore, this study provides essential guidance for the printing industry's transition from experience-based control to data-driven intelligent optimization.

### 1.2 Literature review

#### 1.2.1 Quadratic regression models.

Quadratic regression, as the core tool of response surface methodology (RSM), has been widely applied in the field of printing parameter optimization [5]. The mathematical form of the model is concise$\left(Z=\beta_{\text{0}}+\beta_{\text{1}}X+\beta_{\text{2}}Y+\beta_{\text{3}}X^{\text{2}}+\beta_{\text{4}}Y^{\text{2}}+\beta_{\text{5}}XY\right)$. Its advantages include analytical solutions [6], ease of confidence interval calculation, and predictable extrapolation behavior, making it suitable for establishing preliminary mappings between process parameters and quality indicators [7].

However, the fundamental limitation of QR lies in its a priori assumption of a global quadratic surface [8]. This assumption introduces systematic bias when characterizing complex interactions, particularly when solid density approaches the physical saturation point of ink transfer. At these points, the actual response surface exhibits nonlinear saturation characteristics that the quadratic form cannot accurately capture [9]. The consequence is the potential generation of a “pseudo-optimal point” —a systematic offset between the model-predicted and actual physical optimum [10].

#### 1.2.2 Lasso regression.

Lasso (Least Absolute Shrinkage and Selection Operator) regression is a regularized extension of linear regression that addresses overfitting and performs automatic feature selection by imposing an L1 penalty on regression coefficients [11]. This method has gained popularity in industrial process modeling due to its ability to handle high-dimensional data and produce sparse, interpretable models [12]. In the context of printing process optimization, Lasso has been applied to predict print quality attributes and identify critical process parameters. Compared to standard QR, Lasso offers improved generalization by preventing coefficient inflation [13], making it particularly suitable when the number of candidate variables is large relative to sample size. However, as a linear method with polynomial expansions, Lasso may still struggle to capture highly nonlinear process behaviors such as saturation effects and multi-modal response surfaces.

#### 1.2.3 Random forest.

Random forest is an ensemble learning method that constructs multiple decision trees during training and outputs the average prediction of individual trees [14]. As a non-parametric approach, RF makes no prior assumptions about the functional form of the relationship between inputs and outputs, offering substantial flexibility for modeling complex industrial processes. Key advantages of RF include robustness to outliers, resistance to overfitting (due to the averaging of many trees), and the ability to capture nonlinear interactions without extensive hyperparameter tuning [15]. In printing applications, RF has been used to predict dot gain, optimize ink transfer, and model color reproduction [16]. The method naturally provides measures of feature importance, offering insights into which process parameters most strongly influence print quality. RF has been identified by multiple reviewers as a natural baseline for comparison against neural networks in regression tasks with moderate sample sizes.

#### 1.2.4 Multilayer perceptron.

In contrast to parametric models, multilayer perceptron, a typical class of feedforward neural networks, offers enhanced flexibility for modeling complex industrial processes, grounded in the Universal Approximation Theorem [17]. In printing quality control, MLP has been applied to color prediction, registration error compensation, and defect detection [18–20].

Nevertheless, MLP faces three significant challenges. First, its “black-box” nature reduces engineers’ trust in model decisions [21]. Second, training requires substantial data to avoid overfitting, yet industrial experimentation often faces cost and time constraints [22]. Third, there is a paucity of systematic comparisons between MLP and alternative methods under unified experimental conditions—particularly when sample sizes are moderate, where MLP may be susceptible to overfitting compared to simpler models [23].

#### 1.2.5 Research gaps.

A thorough review of the existing literature reveals a conspicuous absence of systematic comparative studies on modeling methods for determining the optimal solid density in offset printing, a critical process parameter [24]. A number of extant studies have focused on extending the application of traditional models, such as QR, or on architectural innovations of emerging methods, like neural networks [25]. However, these studies have yet to quantitatively compare the actual performance of four representative modeling paradigms—parametric (QR), regularized parametric (Lasso), ensemble (RF), and multilayer perceptron (MLP)— across core dimensions such as prediction, generalization, optimization, and engineering applicability, all under unified experimental and data conditions.

To address this research gap, this study establishes a rigorous statistical comparison framework to evaluate the comprehensive performance of QR, Lasso, RF, and MLP in optimizing solid density for offset printing. Furthermore, existing comparative studies rarely address how validation strategies and temporal dependence in sequential industrial printing data influence model predictive performance and the robustness of estimated optimal process parameters. The research provides empirical evidence to inform methodology selection for printing process optimization and elucidates the applicable boundaries between parametric, regularized, ensemble, and nonparametric models in addressing practical engineering problems.

### 1.3 Research framework and innovations

This study proposes a comparative evaluation framework for the empirical assessment of four modeling methods—QR, Lasso, RF, and MLP—in determining optimal solid density for offset printing. To this end, separate prediction models were built for each of the four-color inks. Employing random 5-fold CV permitted the quantitative evaluation of model performance across dimensions of prediction accuracy (${\text{R}}^{\text{2}}$, RMSE) and the stability of generalization performance (CV%). Paired *t*-tests were conducted *t*o ascertain the significance of the observed differences. A consistency check was performed to assess agreement between models. These mechanisms may provide methodological foundations for intelligent optimization of printing production.

The innovations of this study are threefold. Methodologically, a unified four-tier comparative framework covering parametric QR, regularized parametric Lasso, ensemble RF, and MLP models was established to systematically quantify the applicability boundaries of different modeling approaches for printing optimization. In terms of process mechanism interpretation, non-parametric models (RF and MLP) captured unique nonlinear response characteristics under random 5‑fold CV, including high-quality plateaus for cyan and magenta inks and multi-peak patterns for yellow ink. These features are unobtainable via parametric methods and help explain the formation of pseudo-optimal points, though such interpretations are validation‑strategy dependent. Practically, this study provides ink-specific model selection guidance, bridging the gap between modeling methodology and intelligent printing optimization.

## 2 Methodology

### 2.1 Experimental design

This study developed four modeling methods—QR, Lasso, RF, and MLP—that used solid density (X) and dot gain (Y) as input variables to predict the relative contrast of offset printing (Z) and to compare their fitting accuracy and generalization capability. As illustrated in Fig 1, all four models employed identical inputs, thereby facilitating a direct comparison of their predictive capabilities. QR assumed a quadratic polynomial relationship; Lasso extended QR with L1 regularization for feature selection; RF constructed an ensemble of decision trees; and MLP adaptively learned the input-output mapping from data. This framework provides direct evidence supporting process model optimization and intelligent quality control.

**Fig 1:**
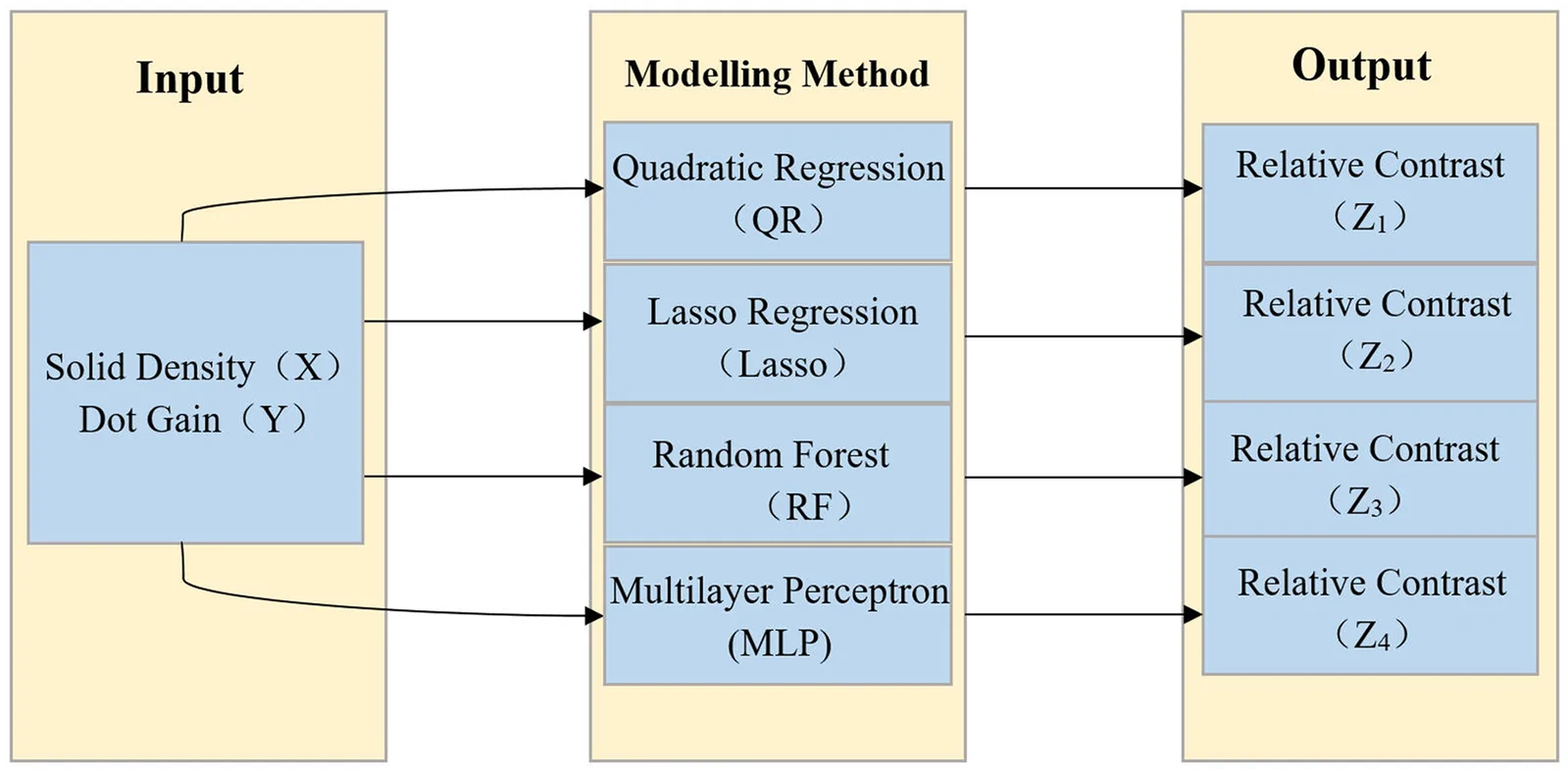
Framework of model construction and comparison. All four models receive identical inputs (solid density X and dot gain Y) and predict relative contrast (Z).

#### 2.1.1 Experimental equipment and materials.

The experiment was conducted under standard industrial conditions, with the configuration as follows:

- Printing equipment: Jiangsu Zhongjing PZ4740E‑AL four‑color offset press, equipped with 175 LPI round dots, standard printing sequence Black–Magenta–Cyan–Yellow, printing pressure 0.15 mm, speed 8000 sheets/h.
- **Measurement instrument:** X‑Rite eXact spectrophotometer (D50 light source, 2° observer), density repeatability ±0.01.
- **Consumables:** Tianjin Toyo TGO‑NO four‑color offset ink, 157 g/m^2^ double‑sided coated paper.
- **Test chart:** IT8.7/4 standard color target, containing four‑color 20‑step gray balance scales.
- **Environmental control:** Temperature 23 ± 1 °C, relative humidity 60 ± 5% RH.

#### 2.1.2 Data collection.

After achieving stable ink–water balance on the printing press, a set of 120 initial sheets was extracted from the continuous printing unit using systematic sampling. After thorough visual inspection to discard defective samples (e.g., ghosting, ink streaks, or soiling), 110 valid samples per color were obtained, yielding a total of 440 data points (110 per color).

The measurements were obtained using a calibrated spectrophotometer to ascertain the solid density (X), dot gain (Y), and relative contrast (Z) for each of the four colors on every sample. Each parameter was measured three times at the same position, and the arithmetic mean was taken as the final value. The quality control threshold was established at ±0.02, based on an instrument repeatability of ±0.01 and industry standards. If the standard deviation of the three replicates exceeded the threshold, the sample was remeasured.

To assess temporal dependence inherent in the continuous sampling protocol, autocorrelation analysis was performed on the Z values for each ink color. The lag-1 autocorrelation coefficients ranged from 0.9642 to 0.9776, indicating strong serial dependence in the data that necessitated careful validation design.

#### 2.1.3 Data analysis.

All data processing and modeling analyses were conducted in MATLAB 2016a (MathWorks, USA). The preprocessing of data and the identification of outliers were conducted using SPSS 26.0. QR and random 5-fold CV were implemented using matrix operations and custom scripts. The Neural Network Toolbox was employed to construct and train the MLP model. The computing platform was composed of an Intel Core i7-11700 processor with 16 GB of RAM, operating on a Windows 11 system.

### 2.2 Predictive modeling

#### 2.2.1 Quadratic regression.

Quadratic regression followed a second-order polynomial relationship:


$$\begin{array}{c} \text{Z}(\text{X},\text{Y})=\beta_{\text{0}}+\beta_{\text{1}}\text{X}+\beta_{\text{2}}\text{Y}+\beta_{\text{3}}{\text{X}}^{\text{2}}+\beta_{\text{4}}{\text{Y}}^{\text{2}}+\beta_{\text{5}}\text{XY}+\epsilon \end{array}$$ (1)

Model coefficients were estimated via ordinary least squares. To maximize Z, we set the first-order partial derivatives to zero and solved the resulting linear system. If the Hessian matrix was invertible, the analytical optimal solution (X, Y) was obtained directly; otherwise, a grid search was adopted [26]. A 100 × 100 grid was used for exhaustive search in full-data fitting and each cross-validation subset.

#### 2.2.2 Lasso regression.

Least Absolute Shrinkage and Selection Operator (Lasso) regression introduced L1 regularization to QR. It added a penalty term to the loss function to drive some coefficients toward zero [27], which enabled automatic feature selection and mitigated overfitting. We adopted the same second-order polynomial features as QR. The regularization parameter λ was optimized via embedded random 5-fold CV within each training subset.

#### 2.2.3 Random forest.

Random forest is an ensemble learning algorithm that integrates multiple decision trees during training and takes the average of individual tree outputs as the final prediction [28]. As a non-parametric method, it requires no predefined functional form and is robust to outliers and overfitting. We trained an ensemble of 50 decision trees using the TreeBagger function in MATLAB, and optimal hyperparameters were determined via grid search within the experimental parameter space.

#### 2.2.4 Multilayer perceptron.

A multilayer perceptron with a single hidden layer was constructed. Based on systematic hyperparameter search over hidden layer sizes [3–6,8,10] using random 5-fold CV, a 5-neuron MLP was adopted for black, cyan, and magenta inks. This configuration exhibits stable performance within 10% of the color-specific optimal accuracy in printing modeling tasks. For yellow ink, the 5-neuron MLP exhibited training instability. Therefore, a 3-neuron MLP was adopted. The hyperbolic tangent (tansig) is used as the hidden layer activation function, and a linear function is used for the output layer. The network was trained using the Levenberg-Marquardt backpropagation algorithm (trainlm) [29], and mean squared error serves as the standard performance metric for model training. Training epochs were set to 300 for full-data modeling and 200 for cross-validation folds. The random seed was fixed to ensure reproducibility. Input and output variables were normalized to [0, 1] using Min-Max normalization, with normalization parameters calculated independently for each training fold. Optimal parameters were determined via grid search within the experimental range of each color.

### 2.3 Model validation and robustness assessment

The generalization ability of the models was evaluated using random 5‑fold CV [30]. In this standard procedure, the full dataset is randomly divided into five mutually exclusive folds. Each fold is successively held out as the validation set, while the remaining four folds serve as the training set. Our implementation followed this scheme, repeating the training and validation cycle to ensure every sample was validated once. To prevent data leakage in the CV process, the normalization parameters (minimum and maximum values) for each fold were derived independently from their corresponding training set and then applied to transform the validation set.

To further assess the robustness of model performance against serial dependence and to mitigate optimistic performance estimates associated with near-duplicate samples across folds, two additional blocked-CV schemes were implemented in this study. Temporal‑block CV divides samples into consecutive blocks according to measurement order, with each block wholly assigned to either training or validation; spatial‑block CV groups samples by the value range of solid density X (not the joint (X, Y) domain). Because solid density increased monotonically with measurement order, the two schemes produced identical or nearly identical folds and should not be regarded as independent validation strategies. These two supplementary schemes complement random CV, providing a more comprehensive assessment of model generalization and robustness. Blocked-CV further enables evaluation of the between-fold variability of the estimated optimal solid density, rather than only predictive performance metrics.

The predictive performance of the models is quantified using the coefficient of determination ($R^{\text{2}}$), root mean square error (RMSE), and coefficient of variation (CV%). Specifically, ${\text{R}}^{\text{2}}$ and RMSE serve as statistical measures of accuracy to assess prediction precision. The CV% (standard deviation/mean × 100%) is calculated on the fold‑wise optimal solid‑density estimates, rather than on prediction metrics, to assess the stability of the estimated optimum across cross‑validation folds. A lower CV% indicates more stable optimal‑density estimation [31].


$$\begin{array}{c} R^{\text{2}}=\text{1}-\frac{\sum_{i=\text{1}}^{n}{\left(Z_{i}-{\hat{Z}}_{i}\right)}^{\text{2}}}{\sum_{i=\text{1}}^{n}{\left(Z_{i}-\overset{―}{Z}\right)}^{\text{2}}} \end{array}$$ (2)

$$\begin{array}{c} \text{RMSE}=\sqrt{\frac{\text{1}}{n}\sum_{i=\text{1}}^{n}{\left(Z_{i}-{\hat{Z}}_{i}\right)}^{\text{2}}} \end{array}$$ (3)

, where $Z_{i}$ is the measured relative contrast of the *i*-th sample, ${\hat{Z}}_{i}\;$is the corresponding model prediction, and $\overset{―}{Z}$ is the mean of the measured values.

To ascertain the statistical significance of performance discrepancies, paired *t*-tests were conducted at the 0.05 significance level on the per-fold differences between model pairs [32]. Normality of the differences was verified using Shapiro-Wilk tests; for cases where the normality assumption was violated, the non-parametric Wilcoxon signed-rank test was used as an alternative. This paired approach is more appropriate than independent two-sample *t*-tests because all models are evaluated on identical training and validation splits within each fold.

In addition, a consistency check was conducted to evaluate inter-model agreement. In this analytical framework, the optimal solution from one model is substituted into other models to predict the corresponding relative contrast value. The discrepancy between the predicted and original optimal values is then used to assess the consistency of the two modeling approaches within the optimal solution region, thereby enhancing the reliability of the process optimization recommendations [33].

### 2.4 Response surface analysis and optimization

#### 2.4.1 Surface reconstruction and visualization.

Within the experimental parameter space, a two-dimensional orthogonal grid was constructed, with adaptive adjustment of grid density according to each color's data distribution to ensure surface smoothness. The coordinates of the grid nodes were entered into each of the four models for forward prediction, respectively. Prediction matrices for relative contrast were obtained and subsequently used to reconstruct the three-dimensional response surfaces. By comparing the topological morphologies of the four response surfaces, the interaction effects between solid density and dot gain and the nonlinear saturation characteristics were intuitively evaluated [34]. All response surfaces were restricted to the convex hull (or data range) of the training data to avoid extrapolation artifacts. Predictions outside the experimental data range were masked as NaN.

#### 2.4.2 Optimal parameter determination.

For the QR model, the optimal point was solved analytically. The first-order partial derivatives of the fitted quadratic surface equation with respect to X and Y were derived and set to zero, yielding a system of linear equations. The solution to this system of equations was the stationary point. When the determinant of the coefficient matrix exceeded a predetermined threshold and the solution lay within the experimental domain, it was accepted as the analytical optimal solution. If the matrix was singular or the solution fell outside the physically feasible domain, a grid search method was employed to conduct an exhaustive search for the global maximum within a reasonable process parameter range [35].

For Lasso, RF, and MLP, a high-resolution grid search method was adopted to sample and predict across the parameter space. The search region was discretized with appropriately fine grid resolution of 100 × 100, and the relative contrast prediction value at each grid node was computed. The grid coordinates ($X^{*}$,${\;Y}^{*}$) corresponding to the maximum prediction value were selected as the optimal process parameters [36,37]. For MLP, these coordinates were mapped back to the actual physical quantities through denormalization. For Lasso and RF, which do not require normalization, the coordinates were used directly.

For blocked-CV, the optimal solid density $X^{*}$ was obtained by training each model on each training block and performing grid search within that block; the reported $X^{*}$ values are the means across the five folds, with standard deviations reported in the corresponding tables.

### 2.5 Ethics and reproducibility statement

This study involves only mathematical modeling of measurements from standard printed test charts and does not involve human participants or animal experiments. Therefore, ethics committee approval was not required. All data were obtained from controlled printing experiments conducted in accordance with academic ethical standards. All experimental data, protocols, code, and supplementary results are available in the Supporting Information (S1 Data, S1 Text, S1–S4 Tables, S1–S12 Figs, S1–S3 Code in S1 File). All key parameters, algorithm settings, and material information are described in the Methodology section to ensure reproducibility.

### 2.6 Inclusivity in global research

The inclusivity questionnaire required by PLOS ONE is not applicable to this study, as it involves no human subjects, field research, or research conducted in low- or middle-income countries. A detailed, step-by-step plan for measuring solid density and determining its optimal values is available as Supporting Information (S1 Text in S1 File: Protocol).

## 3 Results

This study employed four modeling methods—QR, Lasso, RF, and MLP—to characterize the nonlinear relationships among solid density (X), dot gain (Y), and relative contrast (Z) for four-color inks (black, cyan, magenta, and yellow). A comparative analysis was conducted on the prediction accuracy, stability, and response surface characteristics of the four models using 110 sets of process data and random 5-fold CV. In addition, a robustness assessment using blocked-CV was performed to evaluate the sensitivity of the results to serial dependence. The modeling performance, parameter stability, and process recommendations for the four inks are summarized in Table 1.

**Table 1:** Modeling performance and optimal parameters for four-color inks under random 5-fold cross-validation.

Color	Selected model (random CV)	$R^{2}$ (Mean)	RMSE (Mean)	CV% of $X^{*}$	Optimal $X^{*}$
Black	RF	0.9628	0.9339	0.28%	1.67
Cyan	RF	0.9836	0.7604	0.18%	1.44
Magenta	RF	0.9702	0.7351	0.31%	1.42
Yellow	MLP	0.9770	0.6904	0.17%	0.99

**Note:** MLP: multilayer perceptron; RF: random forest; $X^{*}$: optimal solid density; CV%: coefficient of variation of $X^{*}$ across the 5 folds of random CV; Selected model (random CV): best-performing model under random 5-fold CV. This ranking is not valid for blocked-CV, where most models yield negative${\;R}^{\text{2}}$.

### 3.1 Modeling results of black ink

*Prediction accuracy.* RF achieved the best overall performance, with a mean ${\text{R}}^{\text{2}}$ of 0.9628 ± 0.0223 and RMSE of 0.9339 ± 0.2355. MLP delivered comparable performance (${\text{R}}^{\text{2}}\;$= 0.9588 ± 0.0210, RMSE = 1.0012 ± 0.1899). Traditional parametric models showed lower predictive accuracy: QR yielded ${\text{R}}^{\text{2}}\;$= 0.8900 ± 0.0431 and RMSE = 1.6658 ± 0.2261, while Lasso yielded ${\text{R}}^{\text{2}}\;$= 0.8873 ± 0.0488 and RMSE = 1.6794 ± 0.2732 (Table S1 in S1 File). Paired *t*-tests indicated that both RF and MLP significantly outperformed QR (*p* = 0.0035 and 0.0124, respectively), with no significant difference between RF and MLP (*p* = 0.5838). Lasso showed no significant difference from QR (*p* > 0.05). The boxplots (Figs S1, S2 in S1 File) showed tha*t* RF and MLP tended to exhibit more concentrated cross-fold performance distributions, which may indicate improved stability of generalization performance, while the bar chart (Fig S3 in S1 File) visually compared the mean performance of the four models.

*Response surface morphology.* As shown in the four subplots of Fig 2a-2d, constrained by fixed quadratic functional forms, QR (Fig 2a) and Lasso (Fig 2b) may generate gently arched unimodal surfaces with narrow elongated bands of optimal high-contrast regions. The overall surface trends were generally consistent with the distribution of the measured scattered points, showing only slight deviations at the boundaries of the parameter ranges. Notably, both parametric models produced no negative values or abnormal predictions beyond the measured relative contrast range. In contrast, RF (Fig 2c) and MLP (Fig 2d), free from preset functional constraints, exhibited surfaces that more closely followed the sample distribution: RF formed a broad, flat high-contrast plateau around the optimal process parameters, naturally undulating with the measured data, while MLP produced a smoother surface with a continuous gentle slope in the high-value region, showing weaker local fluctuations than RF. All four model surfaces were restricted to the convex hull of the training data to avoid invalid extrapolation beyond the boundaries of the experimental parameter range.

**Fig 2:**
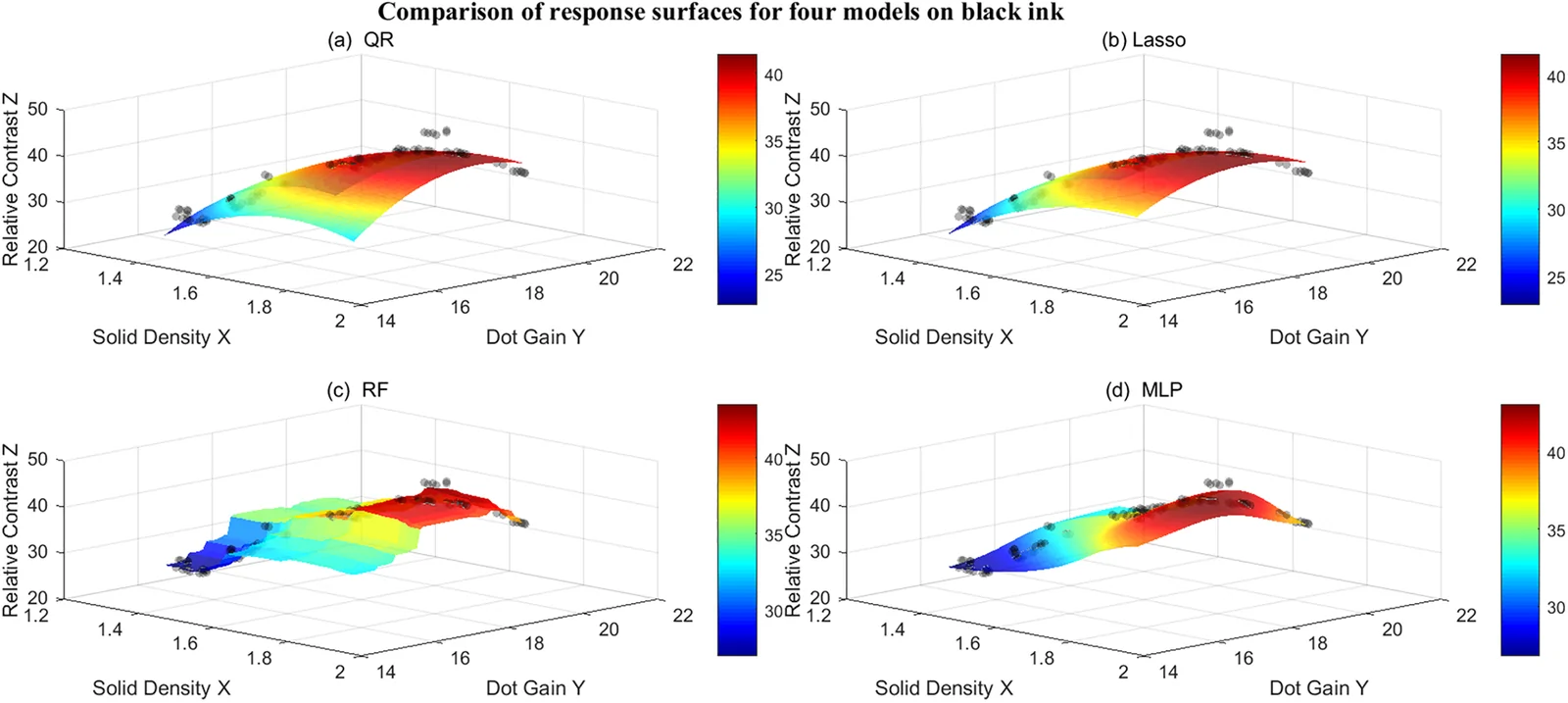
Response surfaces of four models for black ink. (a) Quadratic regression (QR); (b) Lasso; (c) Random forest (RF); (d) Multilayer perceptron (MLP). Gray dots: sample data. All surfaces are restricted to the convex hull of training data.

*Parameter stability.* The mean optimal solid density of RF was 1.6721 ± 0.0046, with a coefficient of variation (CV) of only 0.28%, demonstrating superior stability compared to other models: QR (1.7606 ± 0.0564, CV = 3.20%), Lasso (1.7916 ± 0.0495, CV = 2.76%), and MLP (1.7401 ± 0.0981, CV = 5.64%) (Table S1 in S1 File). RF exhibited the smallest parameter deviation (relative deviation of 0.08%), followed by MLP (0.82%), Lasso (0.95%), and QR (1.16%). Under full-data modeling, RF achieved the maximum predicted relative contrast of 43.5303 at a solid density of 1.6734 and dot gain of 19.1139, outperforming QR (41.4549), Lasso (41.5651), and MLP (43.0389).

*Consistency check.* Cross-prediction was performed by substituting the optimal parameters of each model into the others. The RF optimal point yielded predicted relative contrasts of 42.44 on MLP and 40.66 on QR (Table S1 in S1 File), showing good consistency across models. This stability originates from the broad plateau characteristics of RF and MLP surfaces, whereas QR and Lasso compress the objectively wide optimal region into narrow peaks.

In summary, RF is the selected model under random 5-fold CV for the process–contrast relationship of black ink, with an optimal solid density of 1.67 and target relative contrast ≥ 42.0.

### 3.2 Modeling results of cyan ink

*Prediction accuracy.* RF achieved the best overall performance, with a mean ${\text{R}}^{\text{2}}$ of 0.9836 ± 0.0094 and RMSE of 0.7604 ± 0.2369, followed by MLP (${\text{R}}^{\text{2}\;}$= 0.9753 ± 0.0271, RMSE = 0.8637 ± 0.3567). Traditional parametric models showed lower predictive accuracy: QR yielded ${\text{R}}^{\text{2}}\;$= 0.7635 ± 0.0500, RMSE = 2.9908 ± 0.3917, while Lasso yielded ${\text{R}}^{\text{2}}$= 0.7075 ± 0.0667, RMSE = 3.3153 ± 0.4041 (Table S2 in S1 File). Paired *t*-tests indicated that both RF and MLP significantly outperformed QR (*p* = 0.0004 and 0.0014, respectively), with no significant difference between RF and MLP (*p* = 0.4985). Lasso showed no significant difference from QR (*p* > 0.05). The boxplots (Figs S4, S5 in S1 File) showed tha*t* RF and MLP exhibited more concentrated cross-fold performance distributions, indicating better generalization stability, while the bar chart (Fig S6 in S1 File) visually compared the mean performance of the four models.

*Response surface morphology.* As shown in Fig 3a-3d, constrained by fixed quadratic functional forms, QR (Fig 3a) and Lasso (Fig 3b) may generate steep convex peaked surfaces. The high-value regions were noticeably disconnected from the measured samples, which gradually increased along the solid density axis on the left, failing to capture the progressive rise of the experimental data. Both parametric models exhibited low coefficients of determination. In contrast, RF (Fig 3c) and MLP (Fig 3d), free from preset functional constraints, produced surfaces that closely followed the sample distribution. RF formed a broad, continuous, gently transitioning high-contrast plateau in the optimal parameter region, while MLP exhibited a convex surface with contours tightly adhering to the measured scattered points. All four response surfaces were restricted to the convex hull of the training data to avoid invalid extrapolation beyond the experimental parameter range.

**Fig 3:**
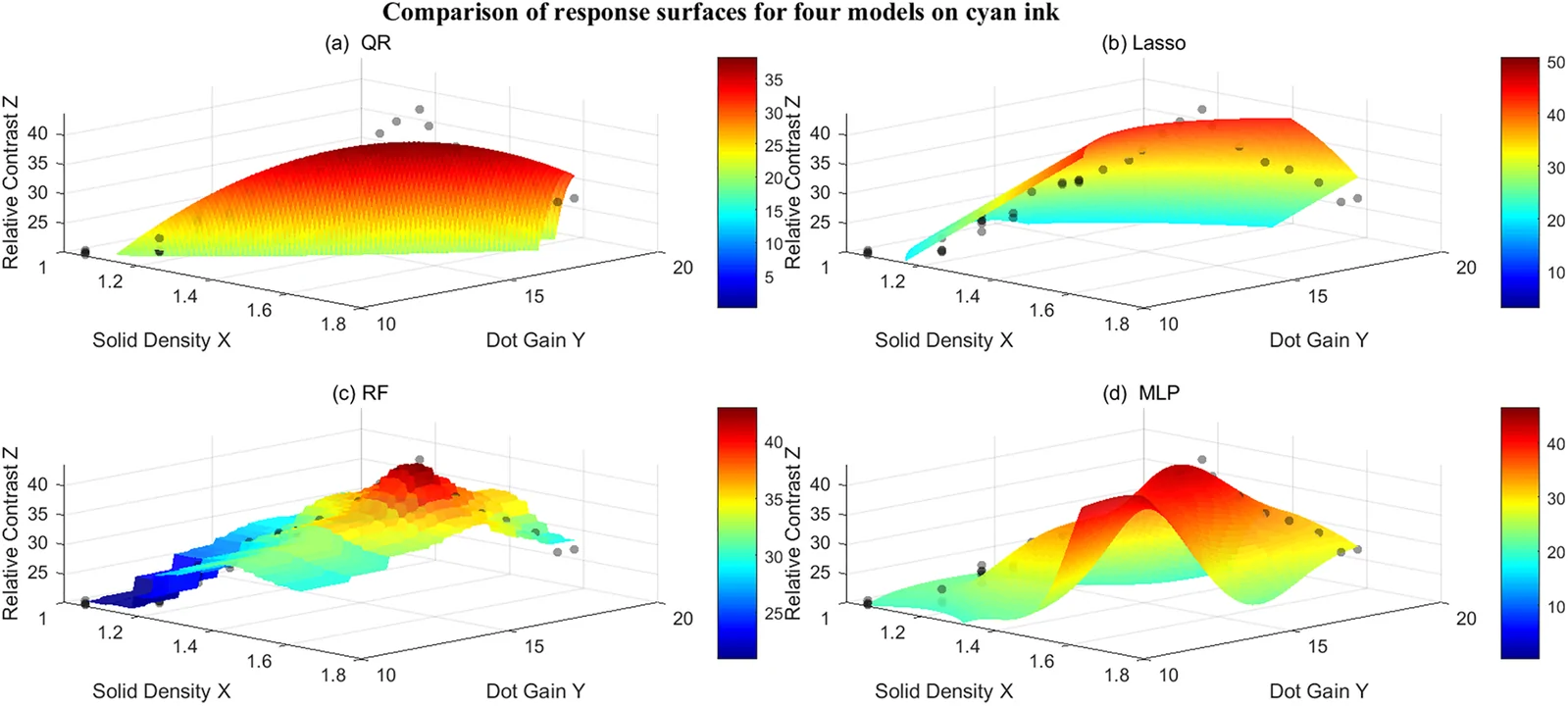
Response surfaces of four models for cyan ink. (a) Quadratic regression (QR); (b) Lasso; (c) Random forest (RF); (d) Multilayer perceptron (MLP). Gray dots: sample data. All surfaces are restricted to the convex hull of training data.

*Parameter stability.* The mean optimal solid density of RF was 1.4378 ± 0.0026, with a coefficient of variation (CV) of 0.18%, QR was 1.4354, Lasso was 1.6300, and MLP was 1.3699 (Table S2 in S1 File). Under full-data modeling, RF achieved a maximum predicted relative contrast of 42.9596 at a solid density of 1.4367 and dot gain of 16.1519, comparable to MLP (43.0462). QR yielded only 38.2823, while Lasso produced an anomalously high peak of 45.8292.

*Consistency check.* The RF optimal point yielded predicted relative contrasts of 42.54 on MLP and 38.24 on QR, and the MLP optimal point yielded 42.43 on RF, showing good consistency across models. This stability originates from the broad plateau characteristics of RF and MLP surfaces.

In summary, RF is the selected model under random 5-fold CV for cyan ink, with an optimal solid density of 1.44 and a target relative contrast ≥ 43.0.

### 3.3 Modeling results of magenta ink

*Prediction accuracy.* Both RF and MLP achieved comparable performance. RF achieved a mean ${\text{R}}^{\text{2}}$ of 0.9702 ± 0.0084 and RMSE of 0.7351 ± 0.1124, while MLP achieved a mean ${\text{R}}^{\text{2}}$ of 0.9778 ± 0.0128 and RMSE of 0.6039 ± 0.0927. Traditional parametric models showed lower predictive accuracy: QR yielded ${\text{R}}^{\text{2}}\;$= 0.9078 ± 0.0474, RMSE = 1.2543 ± 0.0996, while Lasso yielded ${\text{R}}^{\text{2}}\;$= 0.8852 ± 0.0507, RMSE = 1.4157 ± 0.1465 (Table S3 in S1 File). Paired *t*-tests indicated that both RF and MLP significantly outperformed QR (*p* = 0.0342 and 0.0127, respectively), with no significant difference between RF and MLP (*p* = 0.2592). Lasso showed no significant difference from QR (*p* > 0.05). The boxplots (Figs S7, S8 in S1 File) showed tha*t* RF and MLP exhibited more concentrated cross-fold performance distributions. The bar chart with error bars (Fig S9 in S1 File) further showed that the error bar lengths for RF and MLP are significantly shorter than those for QR and Lasso, indicating better generalization stability.

*Response surface morphology*. As shown in Fig 4a-4d, constrained by fixed quadratic polynomial forms, QR (Fig 4a) and Lasso (Fig 4b) may generate steep peaked triangular-shaped surfaces. The optimal response regions were compressed into extremely narrow peaks, and the predicted peak contrast values far exceeded the upper limit of the measured sample range (Lasso reached 63.48). The surface peaks poorly matched the measured scattered points, failing to capture the gradually varying trend of relative contrast with process parameters. In contrast, RF (Fig 4c) and MLP (Fig 4d), free from preset functional constraints, produced surfaces that closely followed the sample distribution. RF exhibited a gently undulating high-contrast plateau with two-stage variation near the optimal parameters, while MLP constructed a continuously smooth high-value surface. All four model surfaces were restricted to the convex hull of the training data to avoid invalid extrapolation beyond the experimental parameter range.

**Fig 4:**
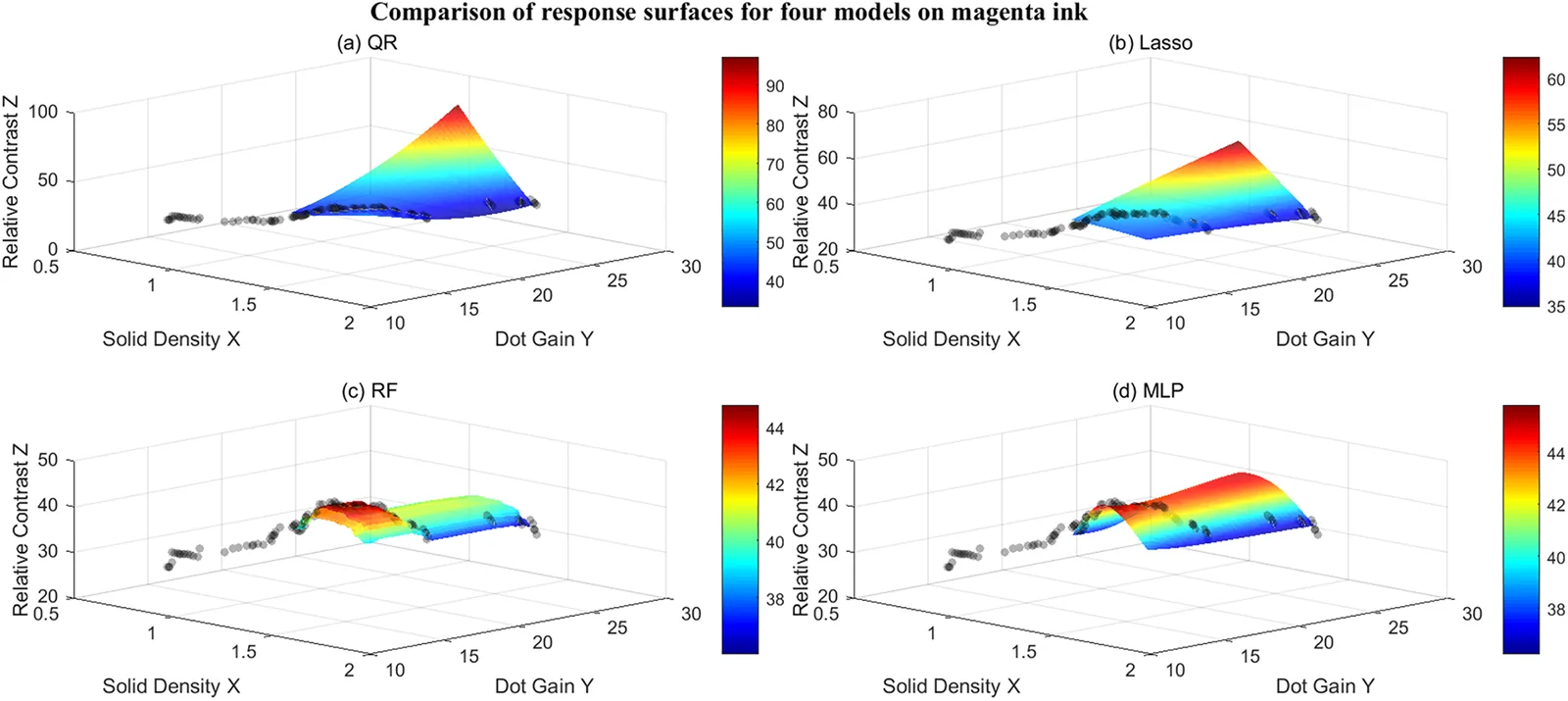
Response surfaces of four models for magenta ink. (a) Quadratic regression (QR); (b) Lasso; (c) Random forest (RF); (d) Multilayer perceptron (MLP). Gray dots: sample data. All surfaces are restricted to the convex hull of training data.

*Parameter stability.* RF showed superior stability compared to MLP. The mean optimal solid density of RF was 1.4205 ± 0.0045 (CV = 0.31%), while MLP exhibited substantially higher variability, with a mean value of 1.4718 and CV of 11.32%. QR was 1.4813 and Lasso was 0.8500 (Table S3 in S1 File). Under full-data modeling, MLP achieved a maximum predicted relative contrast of 45.7903 at a solid density of 1.4671 and dot gain of 14.0000, while RF achieved 44.7679 at a solid density of 1.4165 and dot gain of 15.3671, both close to the upper limit of measured relative contrast. QR yielded 43.3202, whereas Lasso produced an anomalously high predicted peak (63.4824) that exceeded the measured relative contrast range.

*Consistency check.* The RF optimal point yielded predicted relative contrasts of 44.33 on MLP and 43.45 on QR, close to its own peak (44.77). The MLP optimal point yielded 42.65 on RF, showing good consistency across models. This stability originates from the broad plateau characteristics of RF and MLP surfaces.

In summary, RF is the selected model under random 5-fold CV for magenta ink, with an optimal solid density of 1.42 and a target relative contrast ≥ 44.7.

### 3.4 Modeling results of yellow ink

*Prediction accuracy.* MLP with 3 hidden neurons achieved the best overall performance, with a mean ${\text{R}}^{\text{2}}$ of 0.9770 ± 0.0155 and RMSE of 0.6904 ± 0.1425, followed by RF (${\text{R}}^{\text{2}}\;$= 0.9377 ± 0.0420, RMSE = 1.1369 ± 0.2341). Traditional parametric models showed lower predictive accuracy: QR yielded ${\text{R}}^{\text{2}\;}$= 0.8577 ± 0.0423, RMSE = 1.7887 ± 0.2237, while Lasso yielded ${\text{R}}^{\text{2}}\;$= 0.8532 ± 0.0514, RMSE = 1.8070 ± 0.2038 (Table S4 in S1 File). Paired *t*-tests indicated that both MLP and RF significantly outperformed QR (*p* = 0.0008 and 0.0011, respectively), and MLP significantly outperformed RF (*p* = 0.0296). Lasso showed no significant difference from QR (*p* > 0.05). The boxplots (Figs S10, S11 in S1 File) showed that MLP exhibi*t*ed the most concentrated cross-fold performance distributions. The bar chart with error bars (Fig S12 in S1 File) further showed that MLP had the shortest error bars, indicating the best generalization stability.

*Response surface morphology.* As shown in Fig 5a-5d, constrained by fixed quadratic functional forms, QR (Fig 5a) and Lasso (Fig 5b) may generate smooth single-arch surfaces with rounded narrow peaks in the optimal high-contrast regions. Due to the narrow parameter range of yellow ink (solid density 0.85–1.15), the peak flatness of the parametric models was superior to that of magenta and cyan inks. The overall surface trends were generally consistent with the measured scattered points, showing only slight local deviations from the samples. However, the optimal dot gain of Lasso (20.00) exceeded the upper limit of the measured samples (19.04), resulting in poor alignment between the optimal surface region and the measured points. In contrast, RF (Fig 5c) and MLP (Fig 5d), free from preset functional constraints, produced irregular undulating surfaces that closely followed the sample distribution. RF exhibited a multi-peak undulating morphology with broad transition zones between high and low relative contrast regions. However, this multi-peak morphology was observed from surfaces fitted under random 5‑fold CV and should therefore be interpreted with caution, as it does not represent model generalization under blocked-CV validation. MLP exhibited a double-peak surface that closely followed the data variation, although its optimal predicted extreme value slightly exceeded the upper limit of the measured relative contrast range. All four model surfaces were restricted to the convex hull of the training data to avoid invalid extrapolation beyond the parameter boundaries.

**Fig 5:**
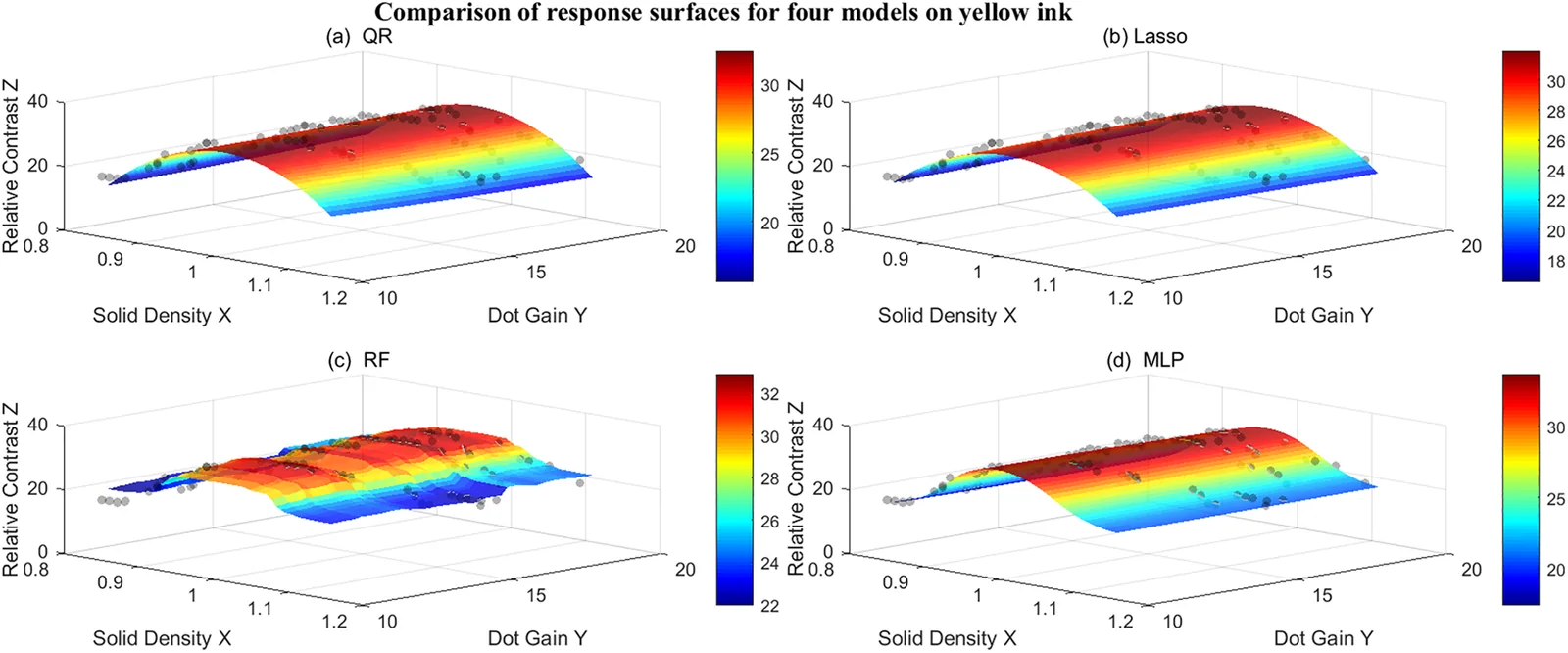
Response surfaces of four models for yellow ink. (a) Quadratic regression (QR); (b) Lasso; (c) Random forest (RF); (d) Multilayer perceptron (MLP). Gray dots: sample data. All surfaces are restricted to the convex hull of training data.

*Parameter stability.* The mean optimal solid density of MLP was 0.9912 ± 0.0017 (CV = 0.17%); RF was 1.0009 ± 0.0054 (CV = 0.54%); QR was 1.0018 ± 0.0021 (CV = 0.21%); and Lasso was 1.0027 ± 0.0070 (CV = 0.70%) (Table S4 in S1 File). Under full-data modeling, MLP achieved a maximum predicted relative contrast of 33.6639 at a solid density of 0.9905, exceeding the upper limit of measured relative contrast, while RF achieved 32.9321, which fell within the measured range. Notably, the 5-neuron MLP exhibited training instability, and the optimal 3-neuron architecture was adopted based on hyperparameter search.

*Consistency check.* The RF optimal point yielded a predicted relative contrast of 32.81 on MLP, consistent with its own peak (32.93). The MLP optimal point fell within the valid data range, and the slightly lower predictions obtained from other models reflect the fundamental differences in model assumptions. The high-contrast region identified by RF remains stable. Under random 5-fold CV, MLP exhibited superior accuracy ($R^{\text{2}}\;$= 0.9770) and was therefore identified as the optimal model for yellow ink under the random CV evaluation framework.

In summary, MLP with 3 hidden neurons is the selected model under random 5-fold CV for yellow ink, with an optimal solid density of 0.99 and a target relative contrast ≥ 32.9.

### 3.5 Robustness of optimal solid density recommendations

To evaluate whether the optimal solid densities are robust to serial dependence (lag-1 autocorrelation: 0.96–0.98), temporal‑block and spatial‑block CV were performed. The key findings are summarized below.

*Absolute performance.* Table 2 reports the mean $R^{\text{2}}$ and RMSE (mean ± SD) for all models under both blocked-CV strategies. Under blocked-CV, $R^{\text{2}}\;$values decreased substantially compared to random CV, with considerable variation across folds. Positive $R^{\text{2}}\;$values were primarily observed in the steady-state folds (Folds 2–4), with black RF reaching 0.59 in Fold 2, cyan MLP reaching 0.94 in Fold 3, magenta RF reaching 0.81 in Fold 4, and yellow MLP reaching 0.90 in Fold 2. This indicates that models retained predictive capability when training and validation blocks shared similar process states. Negative $R^{\text{2}}$ values occurred predominantly in the start-up (Fold 1) and over-inking (Fold 5) folds, reflecting the strong serial dependence in the data.

**Table 2:** Performance metrics (mean $R^{2}$ ± SD and mean RMSE ± SD) for all models under blocked cross-validation strategies.

Ink	Model	Temporal-block CV $R^{2}$	Temporal-block CV RMSE	Spatial-block CV $R^{2}$	Spatial-block CV RMSE
Black	QR	−5.1866 ± 6.7492	4.0574 ± 3.0858	−5.1866 ± 6.7492	4.0574 ± 3.0858
	Lasso	−4.8877 ± 6.5991	3.9036 ± 3.0629	−4.8832 ± 6.6162	3.8819 ± 3.0828
	RF	−0.8640 ± 1.1701	2.3692 ± 0.9252	−0.9555 ± 1.1379	2.4596 ± 1.0249
	MLP	−4.5559 ± 6.2254	3.8197 ± 2.4318	−1.1544 ± 1.9467	2.2432 ± 1.2165
Cyan	QR	−13.6551 ± 14.1343	8.0623 ± 5.9935	−13.6551 ± 14.1343	8.0623 ± 5.9935
	Lasso	−13.6382 ± 13.3114	8.1590 ± 5.8353	−13.7722 ± 13.2238	8.1923 ± 5.8216
	RF	−3.2817 ± 2.1953	4.6299 ± 1.9666	−3.3132 ± 2.3727	4.6227 ± 2.0981
	MLP	−1.7311 ± 4.9462	3.0029 ± 3.6268	−31.5983 ± 67.8713	8.9994 ± 14.4074
Magenta	QR	−87.5452 ± 127.1748	10.9274 ± 12.6297	−87.5452 ± 127.1748	10.9274 ± 12.6297
	Lasso	−153.4272 ± 239.3405	13.9048 ± 16.3502	−154.8304 ± 242.2532	13.9473 ± 16.4140
	RF	−3.5089 ± 3.2709	2.9731 ± 1.9782	−3.6875 ± 3.1709	3.0118 ± 1.8316
	MLP	−59.2478 ± 127.1251	6.9895 ± 9.8943	−18.2026 ± 27.0296	5.3430 ± 6.7908
Yellow	QR	−4.8321 ± 11.0473	1.9492 ± 0.5969	−4.8321 ± 11.0473	1.9492 ± 0.5969
	Lasso	−6.8360 ± 15.2157	2.1214 ± 0.6798	−6.4728 ± 14.8460	1.9687 ± 0.5265
	RF	−9.1718 ± 9.8179	4.2553 ± 2.7833	−7.9739 ± 8.1053	4.1007 ± 2.7458
	MLP	−8.8941 ± 15.9756	4.3523 ± 5.5037	−33.4413 ± 71.3847	7.1992 ± 11.5966

Note: Values are mean ± SD across five folds. Negative mean $R^{\text{2}}\;$values highlight the conservative nature of blocked-CV. Temporal-block and spatial-block CV produced identical or nearly identical folds in this dataset; they are therefore not independent validation strategies.

*Model rankings.* For black, cyan, and magenta inks, the numerical order of model performance under blocked-CV was broadly consistent with random CV (RF/MLP > QR/Lasso). Nevertheless, most models produced negative mean $R^{\text{2}}$ under blocked-CV (Table 2). A negative $R^{\text{2}}$ indicates that a model performs worse than using the training-set mean for prediction; accordingly, these rankings carry no practical predictive meaning and cannot be generalized beyond the random-CV framework. For yellow ink, rankings became unstable, likely due to its narrow data range (X: 0.85–1.15).

*Optimal density robustness.* The temporal-block and spatial-block partitions are not independent validation strategies, as they produced identical or nearly identical folds in this dataset. The optimal solid densities remained consistent across these CV partitioning schemes, with maximum deviations of mean values ranging from 0.0034 to 0.0152 (Table 3), well within the ± 0.05 industrial tolerance, indicating insensitivity to fold‑structure partitioning, though this consistency is dataset‑specific and does not guarantee out‑of‑sample validity under different printing conditions. Nevertheless, stable optimal solid density estimates should not be interpreted as reliable predictive generalization, as shown by the negative R^2^ observed in blocked-CV.

**Table 3:** Comparison of optimal solid densities across the three CV partitioning schemes.

Color	Selected model (random CV)	Random CV $X^{*}$	Temporal-block CV $X^{*}$	Spatial-block CV $X^{*}$	Deviation (Max—Min)
Black	RF	1.6776 ± 0.0054	1.6894 ± 0.0355	1.6928 ± 0.0368	0.0152
Cyan	RF	1.4367 ± 0.0000	1.4259 ± 0.0246	1.4259 ± 0.0246	0.0108
Magenta	RF	1.4194 ± 0.0072	1.4319 ± 0.0308	1.4303 ± 0.0315	0.0125
Yellow	MLP	0.9912 ± 0.0017	0.9946 ± 0.0073	0.9934 ± 0.0133	0.0034

**Note:** Values represent mean ± standard deviation of fold‑wise optimal solid‑density estimates across five folds. Deviation corresponds to maximum‑minimum range of mean optimal $X^{*}\;$across the three CV partitioning schemes. Selected model (random CV): best-performing model under random 5-fold CV. This ranking is not valid for blocked-CV, where most models yield negative${\;R}^{\text{2}}$. As noted in Table 2, the two blocked-CV schemes are not independent.

## 4 Discussion

### 4.1 Mechanistic origins of predictive performance disparities

The performance differences among the four models can be attributed to their divergent assumptions regarding function space [38]. QR and Lasso a priori impose constraints on the process response, confining it to a globally smooth quadratic convex surface. While this strong assumption offers computational efficiency and parametric estimation stability, it also hinders the models’ ability to characterize nonlinear features, such as saturation effects and high-order interactions. As a result, both parametric models may generate “pseudo-optimal points” and, in the case of Lasso, produce anomalously high predicted peaks.

In contrast, RF and MLP, grounded in non-parametric and ensemble learning paradigms, adaptively learn complex input-output mappings without presupposing any global functional form. RF aggregates multiple decision trees, naturally capturing flat plateau regions and local patterns, while MLP, supported by the Universal Approximation Theorem [39], learns smooth continuous mappings. This flexibility enables both RF and MLP to reproduce response surface features observed under random 5‑fold CV, such as plateau-like regions for cyan and magenta, and multi-peak patterns for yellow ink— features not captured by rigid quadratic parameterization. Optimal points from RF and MLP tend to yield higher predictions when projected onto other non-parametric surfaces, while QR and Lasso tended to undervalue these regions because of their artificially compressed peak structures. These observations suggest that the “pseudo-optimal points” produced by parametric models may reflect a mismatch between model topology and the observed data structure under random 5-fold CV, although a physical interpretation of this mismatch requires independent validation. Under random 5‑fold CV, RF and MLP showed substantially lower standard deviations of ${\text{R}}^{\text{2}\;}$compared to QR and Lasso, suggesting enhanced data adaptability and generalization stability within the random‑CV framework.

In summary, under random‑CV, RF and MLP offer clear advantages over QR and Lasso for black, cyan, and magenta inks. These advantages, however, do not hold under blocked‑CV, where predictive performance deteriorated and model rankings became unreliable. For yellow ink, MLP remains the preferred model under random 5‑fold CV due to its superior accuracy (R^2^ = 0.9770) and stability (CV = 0.17%), although its ranking is sensitive to the choice of CV strategy. In engineering practice, the derived optimal density should take precedence over model rankings.

### 4.2 Mechanistic roots of stability disparities and their implications

As shown in Table 1, RF exhibits the highest stability for black (CV = 0.28%), cyan (CV = 0.18%), and magenta (CV = 0.31%) inks. For yellow ink, MLP achieves the highest parameter stability (CV = 0.17%), although its model ranking becomes unstable under blocked-CV, likely due to the narrow data range of yellow ink (X: 0.85–1.15). This color-dependent behavior reflects the differing complexity of each ink's response surface [38].

Specifically, QR and Lasso exhibit pseudo-stability, with remarkably low coefficients of variation resulting from stringent parameter constraints. In essence, the models are constrained to a global quadratic surface. Notwithstanding the variation in the training data subset, the optimization process invariably converges to a unique extreme point that can be obtained through analytical derivation [39]. However, this mathematical stability is achieved at the expense of the capacity to characterize complex process nonlinear features.

RF may achieve notablestability because of its ensemble nature: averaging over multiple decision trees reduces variance and smooths the response surface. This is particularly evident for cyan ink, where RF yields a low coefficient of variation (CV = 0.18%). MLP, while more flexible, exhibits higher sensitivity to training data variations, resulting in greater cross-validation volatility.

Consequently, the CV% of optimal solid density serves as a diagnostic metric for process characteristics. For cyan and magenta, RF's low CV% reflects broad high-quality plateaus, while MLP's moderate CV% for magenta still captures the plateau but with more variability. This observation enables relaxed tolerances for these inks. Conversely, for yellow ink, the MLP CV (0.17%) indicates a narrow optimum under random 5-fold CV, consistent with its multi-peak response observed under the same validation scheme; this interpretation remains tentative given that the multi-peak pattern was not reproduced under blocked-CV.

Black ink serves as an intermediate case within this diagnostic framework. Despite its approximately linear response surface, RF and MLP capture local nonlinear residuals that parametric models fail to account for, thereby reducing prediction error by 39.9% (RF vs QR) and 41.4% (MLP vs QR). The presence of these subtle nonlinearities gives rise to moderate cross-validation variability for MLP, while RF maintains higher stability.

### 4.3 Physicochemical interpretation of chromatic heterogeneity

The following interpretations are hypotheses based on observed data patterns and require independent experimental validation. The color-specific topological features of the response surfaces fitted by RF and MLP under random 5-fold CV may reflect macroscopic manifestations of the unique physicochemical properties of each ink and their interactions with the printing process; however, these features were observed only under random 5-fold CV, and their physical origin has not been independently verified.

Cyan ink displays a “wide plateau + steep edge” structure, which may correspond to the transfer saturation effect of phthalocyanine blue pigment [40]. Upon reaching a critical threshold of solid density, the pigment particles achieve dense coverage on the paper surface. Consequently, the increased ink is primarily absorbed by the fiber pores rather than forming an effective ink layer. This results in a plateau in the relative contrast. Within the designated process window, fluctuations in parameters exert minimal influence on print quality. Due to its global convex assumption, QR and Lasso were unable to recognize this saturation characteristic, and their recommended “sharp vertex” was essentially a rough linear approximation of the plateau region.

Magenta ink displays a similar wide plateau structure possibly originating from the rheological behavior of quinacridone pigments [41]. RF may achieve notable cross-validation stability (CV = 0.31%), while MLP showed higher variability (CV = 11.32%), reflecting the inherent flatness of the plateau. In regions where multiple density values yield comparable relative contrast, different training subsets may select different points as “optimal,” causing the cross-validation mean to shift.

Yellow ink exhibits a fundamentally different “multi-peak fluctuation” characteristic, hypothetically originating from the high transparency of benzidine yellow pigment and its complex emulsion rheological behavior [42]. The dual local optima observed on the response surface fitted under random 5‑fold CV could be consistent with metastable microstructures of ink transfer under varying emulsification states. This hypothesized mechanism, however, remains speculative and requires independent validation beyond the present dataset. The unimodal optimal solution fitted by QR and Lasso may be a “pseudo-optimal point” located in the saddle region between the two peaks observed under random 5-fold CV. MLP reproduced this multi-peak pattern under random 5-fold CV, although the physical origin of this pattern remains unverified and the pattern was not reproduced under blocked-CV. In addition, its peak predicted relative contrast slightly exceeds the measured relative contrast range. Notably, the 3‑neuron MLP achieved notable stability under random 5‑fold CV (CV = 0.17%), though its predicted extreme values exceeded the measured boundary, indicating potential extrapolation risk when combined with degraded generalization under blocked‑CV.

Black ink displays a response surface that is relatively close to a quadratic shape, attributed to the high light absorption and low transparency of carbon black pigment. The relationship between its optical density and ink film thickness approximately follows the linear Lambert-Beer law [43]. RF achieved the best performance for black ink (${\text{R}}^{\text{2}}\;$= 0.9628) with notable stability (CV = 0.28%), indicating the value of ensemble methods even for near-linear surfaces.

### 4.4 Engineering applicability of the four models

QR and Lasso offer advantages in implementation simplicity, computational efficiency, and model interpretability, making them suitable for production environments with limited data or where transparency is required. RF and MLP, despite higher computational cost and reduced interpretability, provide superior prediction accuracy and stability, justifying their deployment when precision is critical and sufficient data are available. The choice of model should therefore balance accuracy requirements against practical constraints such as data availability, computational resources, and engineer trust in model decisions.

For practical deployment, when serial dependence is suspected in the training data, it is advisable to evaluate model performance using both random and blocked-CV strategies. Random CV provides a useful benchmark for comparative studies, while blocked-CV offers a more conservative estimate of model generalization and helps prevent over‑optimistic conclusions. The consistency of optimal solid density recommendations across the CV partitioning schemes examined here should be interpreted as insensitivity to fold structure rather than as out-of-sample validation.

### 4.5 Robustness of optimal density recommendations against serial dependence

The substantial decline in $R^{\text{2}}\;$under blocked-CV indicates that random CV produces optimistic performance estimates due to near-duplicate sample leakage, demonstrating the need for appropriate validation strategies with serially dependent data. The negative $R^{\text{2}}\;$values observed in the start-up (Fold 1) and over-inking (Fold 5) folds indicate that the trained model performs worse than a simple mean predictor when applied to temporally distant validation blocks. This is expected, as these validation blocks lie outside the training data distribution, representing an extreme extrapolation scenario. These results highlight the importance of using blocked-CV to obtain conservative performance estimates.

Despite the decline in absolute${\;R}^{\text{2}}$, the observed stability of estimated optimal solid-density values indicates that the models’ ability to locate the process optimum is less affected by fold structure than their ability to achieve high cross-block prediction accuracy. As shown in Table 3, the optimal densities recommended by RF and MLP varied little across these CV partitioning schemes, with deviations ranging from 0.0034 to 0.0152 across all colors. The convergence of the estimated optimal solid densities across these CV partitioning schemes indicates that the location of the optimum is insensitive to the fold structure examined here, rather than establishing out-of-sample validity. Because the temporal-block and spatial-block partitions produced identical or nearly identical folds in this dataset, they should not be described as substantially different validation strategies.

Therefore, the contribution of this study is not to claim superior predictive accuracy of RF/MLP under all validation conditions, but to show that these methods yield stable optimal-density estimates across the CV partitions explored in this work. For industrial process optimization, this observed stability in locating the optimum is of greater practical value than achieving high absolute $R^{\text{2}}$ values across all process phases. Within this dataset, the insensitivity of the optimal solid density estimates to cross-validation partitioning, rather than the stability of model rankings, is therefore the main practical finding of this study.

## 5 Conclusion

This study systematically compared four modeling methods (QR, Lasso, RF, and MLP) to determine the optimal solid density for four-color offset printing inks, and the core findings are summarized as follows.

- First, under random 5‑fold CV, non-parametric methods (RF and MLP) exhibited significantly higher predictive accuracy than parametric methods (QR and Lasso). RF achieved a mean ${\text{R}}^{\text{2}}$ of 0.9628 and an RMSE of 0.9339 for black ink, while MLP yielded a mean ${\text{R}}^{\text{2}}$ of 0.9770 and an RMSE of 0.6904 for yellow ink. Paired t-tests indicated that both RF and MLP significantly outperformed QR (*p* < 0.05). No significant performance differences were detected between RF and MLP for black, cyan, and magenta inks (*p* > 0.05), while MLP was statistically superior to RF for yellow ink (*p* = 0.0296). Given the high lag‑1 autocorrelation (0.96–0.98) in the sequentially collected data, blocked‑CV was implemented to mitigate temporal‑sample leakage. This approach revealed severe degradation of predictive performance, whereas the estimated optimal solid‑density positions remained insensitive to the fold structure examined here despite substantial declines in absolute ${\text{R}}^{\text{2}}$ values. However, this insensitivity is limited to the present experimental setup and does not guarantee generalization to distinct printing conditions.
- Second, the optimal model tended to vary by ink color. RF was selected for black, cyan, and magenta inks because of its notable predictive accuracy and estimation stability, with a minimum CV value of 0.28% for black ink. A 3-neuron MLP was adopted for yellow ink based on its promising performance under random CV and relatively stable optimal density estimation. It produced the highest relative contrast value (33.66) with improved stability (CV = 0.17%), even though model performance rankings appeared to become unreliable under blocked‑CV.
- Third, under random 5-fold CV fitting, non-parametric models exhibited nonlinear response surface features that were not captured by the parametric models, including plateau-like regions for cyan and magenta inks and multi-peak patterns for yellow ink. These features should be interpreted with caution: they were observed under random 5-fold CV, their physical origin has not been independently verified, and they were not reproduced under blocked-CV. The narrow optimal peaks produced by QR and Lasso may accordingly be regarded as pseudo-optimal points under random 5-fold CV, and the anomalously high predicted values produced by Lasso at local peaks should likewise be treated as a model artifact rather than a process characteristic.

Based on these findings, the optimal solid densities are: black 1.67 (RF), cyan 1.44 (RF), magenta 1.42 (RF), and yellow 0.99 (MLP). This study provides empirical support for data‑driven optimization in offset printing and shows that, for the present dataset, the estimated optimal solid densities are insensitive to the fold structure examined here. This consistency should be interpreted as fold-structure insensitivity rather than out-of-sample validity. The stability of the recommended optima offers greater practical engineering value than the stability of model rankings, which are not reliable under blocked-CV.

Several limitations should be acknowledged. First, all experimental data were collected from a single press run using one ink batch, one paper type, and one set of environmental conditions; therefore, generalization to other equipment, inks, or substrates requires additional validation. Second, the sample size is relatively limited for training the MLP model, and the study considers only two input variables (solid density and dot gain) and a single quality indicator (relative contrast), without exploring multi‑parameter interactions or multi‑objective trade‑offs. Third, the multi‑peak response surface topology for yellow ink is an observation derived from the present dataset under random 5‑fold CV, and its physical origin requires independent experimental validation. Fourth, the printing data exhibit strong serial dependence (lag‑1 autocorrelation: 0.96–0.98), which affects the interpretation of conventional CV results. Although blocked‑CV was adopted to mitigate data leakage and assess the sensitivity of the estimated optimal solid densities to fold structure, the degraded absolute R^2^ values under blocked‑CV should be interpreted with caution.

To address these limitations, future studies will focus on the following three aspects. First, transfer learning will be employed to rapidly adapt to different equipment and materials using limited on-site data. Second, online adaptive algorithms will be developed to track time-varying factors such as ink batch variations and equipment drift. Third, multi-objective optimization frameworks integrating colorimetry, cost, and energy consumption will be established to move beyond single-quality-indicator optimization.

## Supporting information

S1 File S1 Data. Raw measurement data for four-color inks. This file contains 440 measurements of solid density (X), dot gain (Y), and relative contrast (Z) for black, cyan, magenta, and yellow inks (110 data points per color), collected under controlled conditions as described in the main paper. These data form the basis for all modeling and validation analyses. **S1 Text. Protocol.** This document contains detailed experimental procedures, equipment specifications, MATLAB code, and troubleshooting guidelines supporting the methods described in the main paper. All steps are provided to ensure full reproducibility of the reported results. **S1 Table. Performance and robustness statistics for black ink.** Comparison of QR, Lasso, RF, and MLP models for black ink, including prediction performance (R^2^, RMSE), optimal parameters, robustness (CV%), and consistency check results. Paired t-test results are also provided. **S2 Table. Performance and robustness statistics for cyan ink.** Comparison of QR, Lasso, RF, and MLP models for cyan ink, including prediction performance (R^2^, RMSE), optimal parameters, robustness (CV%), and consistency check results. Paired t-test results are also provided. **S3 Table. Performance and robustness statistics for magenta ink.** Comparison of QR, Lasso, RF, and MLP models for magenta ink, including prediction performance (R^2^ RMSE), optimal parameters, robustness (CV%), and consistency check results. Paired t-test results are also provided. **S4 Table. Performance and robustness statistics for yellow ink.** Comparison of QR, Lasso, RF, and MLP models for yellow ink, including prediction performance (R^2^, RMSE), optimal parameters, robustness (CV%), and consistency check results. Paired t-test results are also provided. Note: MLP with 3 hidden neurons was adopted (5-neuron MLP exhibited training instability). **S1 Fig. Distribution of R^2^ for four models on black ink under 5-fold cross-validation.** Boxplots of R^2^ for QR, Lasso, RF, and MLP models. RF and MLP show higher median R^2^ (0.98) than QR and Lasso (0.925–0.94). **S2 Fig. Distribution of RMSE for four models on black ink under 5-fold cross-validation.** Boxplots of RMSE for QR, Lasso, RF, and MLP models. RF and MLP achieve lower median RMSE (1.10) compared to QR and Lasso (1.65). **S3 Fig. Comparison of mean R^2^ and mean RMSE for four models on black ink.**Bar charts of mean R^2^ and mean RMSE. RF and MLP outperform QR and Lasso in both metrics. **S4 Fig. Distribution of R^2^ for four models on cyan ink under 5-fold cross-validation.** Boxplots of R^2^ for QR, Lasso, RF, and MLP models. RF and MLP show higher median R^2^ (0.95) than QR and Lasso (0.78). **S5 Fig. Distribution of RMSE for four models on cyan ink under 5-fold cross-validation.** Boxplots of RMSE for QR, Lasso, RF, and MLP models. RF and MLP achieve lower median RMSE (0.8) compared to QR and Lasso (3.2–3.4). **S6 Fig. Comparison of mean R^2^ and mean RMSE for four models on cyan ink.** Bar charts of mean R^2^ and mean RMSE. RF and MLP outperform QR and Lasso in both metrics. **S7 Fig. Distribution of R^2^ for four models on magenta ink under 5-fold cross-validation.** Boxplots of R^2^ for QR, Lasso, RF, and MLP models. MLP shows the highest median R^2^ (0.98), followed by RF (0.97), outperforming QR and Lasso (0.92). **S8 Fig. Distribution of RMSE for four models on magenta ink under 5-fold cross-validation.** Boxplots of RMSE for QR, Lasso, RF, and MLP models. MLP achieves the lowest median RMSE (0.70), followed by RF (0.80), outperforming QR (1.30) and Lasso (1.40). **S9 Fig. Comparison of mean R^2^ and mean RMSE for four models on magenta ink.** Bar charts of mean R^2^ and mean RMSE. MLP and RF outperform QR and Lasso in both metrics. **S10 Fig. Distribution of R^2^ for four models on yellow ink under 5-fold cross-validation.** Boxplots of R^2^ for QR, Lasso, RF, and MLP models. MLP shows the highest median R^2^ (0.96), followed by RF (0.95), outperforming QR (0.88) and Lasso (0.89). **S11 Fig. Distribution of RMSE for four models on yellow ink under 5-fold cross-validation.** Boxplots of RMSE for QR, Lasso, RF, and MLP models. MLP achieves the lowest median RMSE (0.65), followed by RF (1.0), outperforming QR and Lasso (1.65). **S12 Fig. Comparison of mean R^2^ and mean RMSE for four models on yellow ink.** Bar charts of mean R^2^ and mean RMSE. MLP and RF outperform QR and Lasso in both metrics. **S1 Code. MATLAB scripts for main experiment.** MATLAB scripts for four-model comparison (QR, Lasso, RF, MLP) across four ink colors via 5-fold cross-validation. **S2 Code. Normality Testing and Statistical Analysis.** MATLAB scripts for normality verification and pairwise statistical tests on model performance across four ink colors. **S3 Code. MATLAB scripts for MLP hyperparameter tuning.** MATLAB scripts for determining the optimal number of hidden neurons for black, cyan, magenta, and yellow inks via 5-fold cross-validation. Hidden layer sizes [3–6,8,10] were tested.
(ZIP)
