# Supplementary material for: Comparative study of quadratic regression, Lasso, random forest, and multilayer perceptron for modeling optimal solid density in offset printing
Source: PLoS One. 2026 Oct 21;21(10):e0361203. doi: 10.1371/journal.pone.0361203 (PMC13686465; doi:10.1371/journal.pone.0361203)
Supplement: Supplementary file 1 [file pone.0361203.s001.zip › Supporting Information/S1 Code.docx]

## ## 1. Black Ink

% ==================== 1. Data Preparation ====================

fprintf('=== Black Ink Data Preparation ===\n');

DATA = [

1.33	15.81	27.8

1.33	15.85	27.6

1.35	15.85	27.7

1.35	15.83	27.8

1.37	15.63	27.5

1.36	15.42	27.2

1.38	15.41	27.1

1.38	15.52	26.8

1.39	15.51	26.7

1.4	15.78	26.7

1.41	15.75	26.5

1.41	15.66	27.1

1.42	15.65	27.1

1.42	15.46	26.8

1.43	15.45	26.9

1.43	16.21	31.1

1.43	16.22	31

1.43	16.17	30.6

1.44	16.16	30.5

1.44	16.39	29.7

1.44	16.38	29.6

1.43	16.23	29.5

1.45	16.22	29.4

1.45	16.11	29.5

1.45	16.12	29.4

1.45	16.46	30.3

1.46	16.47	30.4

1.46	16.5	31.5

1.46	16.52	31.6

1.47	16.53	31.7

1.47	16.53	32.4

1.48	16.54	32.5

1.48	17.12	33.6

1.49	17.11	33.7

1.49	16.52	36.5

1.5	16.51	36.4

1.5	18.69	36.6

1.5	18.68	36.5

1.5	18.72	36.7

1.51	18.71	36.6

1.51	18.73	36.5

1.52	18.72	36.4

1.52	19.04	37.1

1.52	19.03	37

1.53	19.05	37.2

1.53	19.05	36.9

1.53	19.04	36.8

1.54	19.04	36.8

1.54	19.06	37.5

1.55	19.05	37.4

1.55	18.46	38.1

1.56	18.51	38.2

1.56	19.34	38.3

1.57	19.32	38.2

1.57	19.26	38.6

1.58	19.25	38.5

1.58	19.36	38.9

1.59	19.35	38.8

1.59	18.64	38.9

1.59	17.26	39.2

1.6	17.25	39.1

1.6	17.45	39.3

1.61	17.44	39.2

1.61	17.89	39.1

1.61	17.88	39.2

1.62	17.89	39.2

1.62	17.91	39.1

1.62	17.9	39

1.63	17.9	39

1.63	17.43	39.3

1.64	17.42	39.4

1.64	17.42	39.1

1.64	17.43	39.2

1.65	17.42	39.1

1.65	17.41	41.3

1.66	17.4	41.2

1.66	19.11	44.5

1.67	19.1	44.4

1.67	19.6	44.2

1.68	19.5	44.1

1.68	19.14	44.5

1.69	19.13	44.3

1.69	18.11	42.9

1.7	18.12	42.8

1.7	18.44	42.2

1.71	18.43	42.1

1.71	18.47	42.7

1.73	18.45	42.6

1.73	18.52	42.5

1.74	18.51	42.4

1.74	18.91	41.7

1.74	18.9	41.6

1.75	18.9	41.5

1.75	18.93	41.6

1.76	18.94	41.7

1.76	18.98	41.9

1.77	18.99	41.8

1.77	18.97	41.9

1.78	18.96	41.8

1.78	18.97	41.9

1.79	18.96	41.8

1.79	19.74	38.9

1.81	19.73	38.8

1.81	19.99	36.9

1.82	20	37

1.82	20.01	37.1

1.83	20	37.2

1.83	20.02	37

1.84	20.01	37.1

1.84	20.03	36.9

];

X = DATA(:, 1);

Y = DATA(:, 2);

Z = DATA(:, 3);

n = length(X);

fprintf('Sample size: %d\n', n);

fprintf('Solid density range: %.2f - %.2f\n', min(X), max(X));

fprintf('Dot gain range: %.2f - %.2f\n', min(Y), max(Y));

fprintf('Contrast range: %.2f - %.2f\n\n', min(Z), max(Z));

% ==================== 1.5 Autocorrelation Analysis ====================

fprintf('\n=== Autocorrelation Analysis ===\n');

% Manual autocorrelation calculation

max_lag = 20;

Z_mean = mean(Z);

Z_var = var(Z);

acf_manual = zeros(max_lag + 1, 1);

for lag = 0:max_lag

if lag == 0

acf_manual(lag+1) = 1;

else

sum_cov = 0;

for i = 1:(n-lag)

sum_cov = sum_cov + (Z(i) - Z_mean) * (Z(i+lag) - Z_mean);

end

acf_manual(lag+1) = sum_cov / ((n-lag) * Z_var);

end

end

lags_manual = (0:max_lag)';

conf = 1.96 / sqrt(n);

figure('Position', [100, 100, 800, 500]);

stem(lags_manual, acf_manual, 'filled', 'LineWidth', 1.5);

hold on;

plot([0, max_lag], [conf, conf], 'r--', 'LineWidth', 1);

plot([0, max_lag], [-conf, -conf], 'r--', 'LineWidth', 1);

xlabel('Lag'); ylabel('Autocorrelation Coefficient');

title('Autocorrelation Function of Black Ink Contrast Values');

grid on;

saveas(gcf, 'Black_Ink_Autocorrelation.png');

fprintf('Lag-1 autocorrelation coefficient: %.4f\n', acf_manual(2));

% ==================== 2. Full-Data Analysis (Baseline) ====================

fprintf('=== Full-Data Baseline Analysis ===\n');

% 2.1 Quadratic regression full-data fitting

A_full = [ones(size(X)), X, Y, X.^2, Y.^2, X.*Y];

coeff_full = A_full \ Z;

Z_pred_quad_full = A_full * coeff_full;

R2_quad_full = 1 - sum((Z - Z_pred_quad_full).^2) / sum((Z - mean(Z)).^2);

B0 = coeff_full(1); B1 = coeff_full(2); B2 = coeff_full(3);

B3 = coeff_full(4); B4 = coeff_full(5); B5 = coeff_full(6);

M = [2*B3, B5; B5, 2*B4];

RHS = [-B1; -B2];

if abs(det(M)) > 1e-10

XY_opt_quad = M \ RHS;

X_opt_quad = XY_opt_quad(1);

Y_opt_quad = XY_opt_quad(2);

else

X_search = linspace(min(X), max(X), 100);

Y_search = linspace(min(Y), max(Y), 100);

max_Z = -inf;

for i = 1:length(X_search)

for j = 1:length(Y_search)

Z_val = B0 + B1*X_search(i) + B2*Y_search(j) + ...

B3*X_search(i)^2 + B4*Y_search(j)^2 + ...

B5*X_search(i)*Y_search(j);

if Z_val > max_Z

max_Z = Z_val;

X_opt_quad = X_search(i);

Y_opt_quad = Y_search(j);

end

end

end

end

Z_opt_quad = B0 + B1*X_opt_quad + B2*Y_opt_quad + B3*X_opt_quad^2 + ...

B4*Y_opt_quad^2 + B5*X_opt_quad*Y_opt_quad;

fprintf('\nQuadratic Regression (full data):\n');

fprintf(' R² = %.4f\n', R2_quad_full);

fprintf(' Optimal solid density X = %.4f\n', X_opt_quad);

fprintf(' Optimal dot gain Y = %.4f\n', Y_opt_quad);

fprintf(' Predicted maximum contrast Z = %.4f\n', Z_opt_quad);

% 2.2 Lasso full-data fitting

fprintf('\nLasso Regression (full data):\n');

A_lasso_full = [X, Y, X.^2, Y.^2, X.*Y];

[B_lasso, info_lasso] = lasso(A_lasso_full, Z, 'CV', 5);

idx_lasso = info_lasso.IndexMinMSE;

intercept_lasso = info_lasso.Intercept(idx_lasso);

coef_lasso = B_lasso(:, idx_lasso);

Z_pred_lasso_full = intercept_lasso + A_lasso_full * coef_lasso;

R2_lasso_full = 1 - sum((Z - Z_pred_lasso_full).^2) / sum((Z - mean(Z)).^2);

% Grid search for Lasso optimal point

X_search = linspace(1.4, 1.8, 100);

Y_search = linspace(15, 20, 100);

max_Z_lasso = -inf;

best_X_lasso_full = NaN;

best_Y_lasso_full = NaN;

for i = 1:length(X_search)

for j = 1:length(Y_search)

Z_val = intercept_lasso + coef_lasso(1)*X_search(i) + coef_lasso(2)*Y_search(j) + ...

coef_lasso(3)*X_search(i)^2 + coef_lasso(4)*Y_search(j)^2 + ...

coef_lasso(5)*X_search(i)*Y_search(j);

if Z_val > max_Z_lasso

max_Z_lasso = Z_val;

best_X_lasso_full = X_search(i);

best_Y_lasso_full = Y_search(j);

end

end

end

fprintf(' R² = %.4f\n', R2_lasso_full);

fprintf(' Optimal solid density X = %.4f\n', best_X_lasso_full);

fprintf(' Optimal dot gain Y = %.4f\n', best_Y_lasso_full);

fprintf(' Predicted maximum contrast Z = %.4f\n', max_Z_lasso);

% 2.3 Random forest full-data fitting

fprintf('\nRandom Forest (full data):\n');

rf_full = TreeBagger(50, [X, Y], Z, 'Method', 'regression');

Z_pred_rf_full = predict(rf_full, [X, Y]);

R2_rf_full = 1 - sum((Z - Z_pred_rf_full).^2) / sum((Z - mean(Z)).^2);

% Grid search for RF optimal point

X_search = linspace(1.4, 1.8, 100);

Y_search = linspace(15, 20, 100);

max_Z_rf = -inf;

best_X_rf_full = NaN;

best_Y_rf_full = NaN;

for i = 1:length(X_search)

for j = 1:length(Y_search)

Z_val = predict(rf_full, [X_search(i), Y_search(j)]);

if Z_val > max_Z_rf && ~isnan(Z_val)

max_Z_rf = Z_val;

best_X_rf_full = X_search(i);

best_Y_rf_full = Y_search(j);

end

end

end

fprintf(' R² = %.4f\n', R2_rf_full);

fprintf(' Optimal solid density X = %.4f\n', best_X_rf_full);

fprintf(' Optimal dot gain Y = %.4f\n', best_Y_rf_full);

fprintf(' Predicted maximum contrast Z = %.4f\n', max_Z_rf);

% 2.4 Neural network full-data fitting

fprintf('\nNeural Network (full data):\n');

X_min = min(X); X_max = max(X);

Y_min = min(Y); Y_max = max(Y);

Z_min = min(Z); Z_max = max(Z);

X_norm = (X - X_min) / (X_max - X_min);

Y_norm = (Y - Y_min) / (Y_max - Y_min);

Z_norm = (Z - Z_min) / (Z_max - Z_min);

rng(42);

net_full = feedforwardnet(5);

net_full.trainFcn = 'trainlm';

net_full.trainParam.epochs = 300;

net_full.trainParam.showWindow = false;

inputs_full = [X_norm'; Y_norm'];

targets_full = Z_norm';

[net_full, ~] = train(net_full, inputs_full, targets_full);

Z_pred_norm = net_full(inputs_full);

Z_pred_nn_full = Z_pred_norm' * (Z_max - Z_min) + Z_min;

R2_nn_full = 1 - sum((Z - Z_pred_nn_full).^2) / sum((Z - mean(Z)).^2);

X_search = linspace(1.4, 1.8, 100);

Y_search = linspace(15, 20, 100);

max_Z_nn = -inf;

best_X_nn_full = NaN;

best_Y_nn_full = NaN;

for i = 1:length(X_search)

for j = 1:length(Y_search)

X_norm_val = (X_search(i) - X_min) / (X_max - X_min);

Y_norm_val = (Y_search(j) - Y_min) / (Y_max - Y_min);

Z_pred_val = net_full([X_norm_val; Y_norm_val]) * (Z_max - Z_min) + Z_min;

if Z_pred_val > max_Z_nn

max_Z_nn = Z_pred_val;

best_X_nn_full = X_search(i);

best_Y_nn_full = Y_search(j);

end

end

end

fprintf(' R² = %.4f\n', R2_nn_full);

fprintf(' Optimal solid density X = %.4f\n', best_X_nn_full);

fprintf(' Optimal dot gain Y = %.4f\n', best_Y_nn_full);

fprintf(' Predicted maximum contrast Z = %.4f\n', max_Z_nn);

% ==================== 3. Random 5-Fold Cross-Validation ====================

fprintf('\n\n=== Random 5-Fold Cross-Validation Method Comparison ===\n');

K = 5;

rng(42);

indices = crossvalind('Kfold', n, K);

% Storage for 4 methods' results (random CV)

quadratic_R2 = zeros(K, 1);

quadratic_RMSE = zeros(K, 1);

lasso_R2 = zeros(K, 1);

lasso_RMSE = zeros(K, 1);

rf_R2 = zeros(K, 1);

rf_RMSE = zeros(K, 1);

nn_R2 = zeros(K, 1);

nn_RMSE = zeros(K, 1);

best_X_quadratic = zeros(K, 1);

best_X_lasso = zeros(K, 1);

best_X_rf = zeros(K, 1);

best_X_nn = zeros(K, 1);

best_Y_quadratic = zeros(K, 1);

best_Y_lasso = zeros(K, 1);

best_Y_rf = zeros(K, 1);

best_Y_nn = zeros(K, 1);

for fold = 1:K

fprintf('\n--- Fold %d Cross-Validation ---\n', fold);

test_idx = (indices == fold);

train_idx = ~test_idx;

X_train = X(train_idx);

Y_train = Y(train_idx);

Z_train = Z(train_idx);

X_test = X(test_idx);

Y_test = Y(test_idx);

Z_test = Z(test_idx);

SS_tot = sum((Z_test - mean(Z_test)).^2);

% ======= Method 1: Quadratic Regression =======

A_train = [ones(size(X_train)), X_train, Y_train, ...

X_train.^2, Y_train.^2, X_train.*Y_train];

coefficients = A_train \ Z_train;

A_test = [ones(size(X_test)), X_test, Y_test, ...

X_test.^2, Y_test.^2, X_test.*Y_test];

Z_pred_quad = A_test * coefficients;

SS_res = sum((Z_test - Z_pred_quad).^2);

quadratic_R2(fold) = 1 - SS_res/SS_tot;

quadratic_RMSE(fold) = sqrt(mean((Z_test - Z_pred_quad).^2));

B0c = coefficients(1); B1c = coefficients(2); B2c = coefficients(3);

B3c = coefficients(4); B4c = coefficients(5); B5c = coefficients(6);

M = [2*B3c, B5c; B5c, 2*B4c];

RHS = [-B1c; -B2c];

if abs(det(M)) > 1e-10

XY_opt = M \ RHS;

best_X_quadratic(fold) = XY_opt(1);

best_Y_quadratic(fold) = XY_opt(2);

else

X_search_local = linspace(min(X_train), max(X_train), 100);

Y_search_local = linspace(min(Y_train), max(Y_train), 100);

max_Z_val = -inf;

for i = 1:length(X_search_local)

for j = 1:length(Y_search_local)

Z_val = B0c + B1c*X_search_local(i) + B2c*Y_search_local(j) + ...

B3c*X_search_local(i)^2 + B4c*Y_search_local(j)^2 + ...

B5c*X_search_local(i)*Y_search_local(j);

if Z_val > max_Z_val

max_Z_val = Z_val;

best_X_quadratic(fold) = X_search_local(i);

best_Y_quadratic(fold) = Y_search_local(j);

end

end

end

end

% ======= Method 2: Lasso Regression =======

A_train_lasso = [X_train, Y_train, X_train.^2, Y_train.^2, X_train.*Y_train];

A_test_lasso = [X_test, Y_test, X_test.^2, Y_test.^2, X_test.*Y_test];

[B, info] = lasso(A_train_lasso, Z_train, 'CV', 5);

idx = info.IndexMinMSE;

intercept = info.Intercept(idx);

coef = B(:, idx);

Z_pred_lasso = intercept + A_test_lasso * coef;

SS_res = sum((Z_test - Z_pred_lasso).^2);

lasso_R2(fold) = 1 - SS_res/SS_tot;

lasso_RMSE(fold) = sqrt(mean((Z_test - Z_pred_lasso).^2));

X_search_local = linspace(min(X_train), max(X_train), 100);

Y_search_local = linspace(min(Y_train), max(Y_train), 100);

max_Z_val = -inf;

for i = 1:length(X_search_local)

for j = 1:length(Y_search_local)

Z_val = intercept + coef(1)*X_search_local(i) + coef(2)*Y_search_local(j) + ...

coef(3)*X_search_local(i)^2 + coef(4)*Y_search_local(j)^2 + ...

coef(5)*X_search_local(i)*Y_search_local(j);

if Z_val > max_Z_val

max_Z_val = Z_val;

best_X_lasso(fold) = X_search_local(i);

best_Y_lasso(fold) = Y_search_local(j);

end

end

end

% ======= Method 3: Random Forest =======

rf_model = TreeBagger(50, [X_train, Y_train], Z_train, 'Method', 'regression');

Z_pred_rf = predict(rf_model, [X_test, Y_test]);

SS_res = sum((Z_test - Z_pred_rf).^2);

rf_R2(fold) = 1 - SS_res/SS_tot;

rf_RMSE(fold) = sqrt(mean((Z_test - Z_pred_rf).^2));

X_search_local = linspace(min(X_train), max(X_train), 100);

Y_search_local = linspace(min(Y_train), max(Y_train), 100);

max_Z_val = -inf;

for i = 1:length(X_search_local)

for j = 1:length(Y_search_local)

Z_val = predict(rf_model, [X_search_local(i), Y_search_local(j)]);

if Z_val > max_Z_val && ~isnan(Z_val)

max_Z_val = Z_val;

best_X_rf(fold) = X_search_local(i);

best_Y_rf(fold) = Y_search_local(j);

end

end

end

% ======= Method 4: Neural Network =======

X_min_train = min(X_train); X_max_train = max(X_train);

Y_min_train = min(Y_train); Y_max_train = max(Y_train);

Z_min_train = min(Z_train); Z_max_train = max(Z_train);

X_norm_train = (X_train - X_min_train) / (X_max_train - X_min_train);

Y_norm_train = (Y_train - Y_min_train) / (Y_max_train - Y_min_train);

Z_norm_train = (Z_train - Z_min_train) / (Z_max_train - Z_min_train);

rng(fold * 100);

net = feedforwardnet(5);

net.trainFcn = 'trainlm';

net.trainParam.epochs = 200;

net.trainParam.showWindow = false;

inputs_train = [X_norm_train'; Y_norm_train'];

targets_train = Z_norm_train';

[net_trained, ~] = train(net, inputs_train, targets_train);

X_norm_test = (X_test - X_min_train) / (X_max_train - X_min_train);

Y_norm_test = (Y_test - Y_min_train) / (Y_max_train - Y_min_train);

inputs_test = [X_norm_test'; Y_norm_test'];

Z_norm_pred = net_trained(inputs_test);

Z_pred_nn = Z_norm_pred' * (Z_max_train - Z_min_train) + Z_min_train;

SS_res = sum((Z_test - Z_pred_nn).^2);

nn_R2(fold) = 1 - SS_res/SS_tot;

nn_RMSE(fold) = sqrt(mean((Z_test - Z_pred_nn).^2));

X_search_local = linspace(min(X_train), max(X_train), 100);

Y_search_local = linspace(min(Y_train), max(Y_train), 100);

max_Z_val = -inf;

for i = 1:length(X_search_local)

for j = 1:length(Y_search_local)

X_norm_val = (X_search_local(i) - X_min_train) / (X_max_train - X_min_train);

Y_norm_val = (Y_search_local(j) - Y_min_train) / (Y_max_train - Y_min_train);

Z_pred_val = net_trained([X_norm_val; Y_norm_val]) * ...

(Z_max_train - Z_min_train) + Z_min_train;

if Z_pred_val > max_Z_val

max_Z_val = Z_pred_val;

best_X_nn(fold) = X_search_local(i);

best_Y_nn(fold) = Y_search_local(j);

end

end

end

fprintf('QR: R²=%.4f, RMSE=%.4f, Optimal X=%.4f\n', ...

quadratic_R2(fold), quadratic_RMSE(fold), best_X_quadratic(fold));

fprintf('Lasso: R²=%.4f, RMSE=%.4f, Optimal X=%.4f\n', ...

lasso_R2(fold), lasso_RMSE(fold), best_X_lasso(fold));

fprintf('RF: R²=%.4f, RMSE=%.4f, Optimal X=%.4f\n', ...

rf_R2(fold), rf_RMSE(fold), best_X_rf(fold));

fprintf('NN: R²=%.4f, RMSE=%.4f, Optimal X=%.4f\n', ...

nn_R2(fold), nn_RMSE(fold), best_X_nn(fold));

end

% ==================== 3.5 Temporal Block Cross-Validation ====================

fprintf('\n\n=== Temporal Block Cross-Validation (Temporal Block CV) ===\n');

fprintf('Description: Data is divided into 5 consecutive blocks in collection order, each block as one fold\n');

fprintf('Purpose: Control the impact of time-series autocorrelation on performance estimation\n');

block_size = floor(n / K);

indices_block = zeros(n, 1);

for fold = 1:K

start_idx = (fold-1) * block_size + 1;

end_idx = min(fold * block_size, n);

indices_block(start_idx:end_idx) = fold;

end

if end_idx < n

indices_block(end_idx+1:end) = K;

end

fprintf('Sample ranges per fold (in collection order):\n');

for fold = 1:K

idx_range = find(indices_block == fold);

fprintf(' Fold %d: Samples %d - %d (%d samples)\n', fold, min(idx_range), max(idx_range), length(idx_range));

end

% Storage for temporal block CV results

quadratic_R2_block = zeros(K, 1);

quadratic_RMSE_block = zeros(K, 1);

lasso_R2_block = zeros(K, 1);

lasso_RMSE_block = zeros(K, 1);

rf_R2_block = zeros(K, 1);

rf_RMSE_block = zeros(K, 1);

nn_R2_block = zeros(K, 1);

nn_RMSE_block = zeros(K, 1);

best_X_quadratic_block = zeros(K, 1);

best_X_lasso_block = zeros(K, 1);

best_X_rf_block = zeros(K, 1);

best_X_nn_block = zeros(K, 1);

best_Y_quadratic_block = zeros(K, 1);

best_Y_lasso_block = zeros(K, 1);

best_Y_rf_block = zeros(K, 1);

best_Y_nn_block = zeros(K, 1);

for fold = 1:K

fprintf('\n--- Fold %d Temporal Block Cross-Validation ---\n', fold);

test_idx = (indices_block == fold);

train_idx = ~test_idx;

X_train = X(train_idx);

Y_train = Y(train_idx);

Z_train = Z(train_idx);

X_test = X(test_idx);

Y_test = Y(test_idx);

Z_test = Z(test_idx);

SS_tot = sum((Z_test - mean(Z_test)).^2);

% ======= Method 1: Quadratic Regression =======

A_train = [ones(size(X_train)), X_train, Y_train, ...

X_train.^2, Y_train.^2, X_train.*Y_train];

coefficients = A_train \ Z_train;

A_test = [ones(size(X_test)), X_test, Y_test, ...

X_test.^2, Y_test.^2, X_test.*Y_test];

Z_pred_quad = A_test * coefficients;

SS_res = sum((Z_test - Z_pred_quad).^2);

quadratic_R2_block(fold) = 1 - SS_res/SS_tot;

quadratic_RMSE_block(fold) = sqrt(mean((Z_test - Z_pred_quad).^2));

B0c = coefficients(1); B1c = coefficients(2); B2c = coefficients(3);

B3c = coefficients(4); B4c = coefficients(5); B5c = coefficients(6);

M = [2*B3c, B5c; B5c, 2*B4c];

RHS = [-B1c; -B2c];

if abs(det(M)) > 1e-10

XY_opt = M \ RHS;

best_X_quadratic_block(fold) = XY_opt(1);

best_Y_quadratic_block(fold) = XY_opt(2);

else

X_search_local = linspace(min(X_train), max(X_train), 100);

Y_search_local = linspace(min(Y_train), max(Y_train), 100);

max_Z_val = -inf;

for i = 1:length(X_search_local)

for j = 1:length(Y_search_local)

Z_val = B0c + B1c*X_search_local(i) + B2c*Y_search_local(j) + ...

B3c*X_search_local(i)^2 + B4c*Y_search_local(j)^2 + ...

B5c*X_search_local(i)*Y_search_local(j);

if Z_val > max_Z_val

max_Z_val = Z_val;

best_X_quadratic_block(fold) = X_search_local(i);

best_Y_quadratic_block(fold) = Y_search_local(j);

end

end

end

end

% ======= Method 2: Lasso Regression =======

A_train_lasso = [X_train, Y_train, X_train.^2, Y_train.^2, X_train.*Y_train];

A_test_lasso = [X_test, Y_test, X_test.^2, Y_test.^2, X_test.*Y_test];

[B, info] = lasso(A_train_lasso, Z_train, 'CV', 5);

idx = info.IndexMinMSE;

intercept = info.Intercept(idx);

coef = B(:, idx);

Z_pred_lasso = intercept + A_test_lasso * coef;

SS_res = sum((Z_test - Z_pred_lasso).^2);

lasso_R2_block(fold) = 1 - SS_res/SS_tot;

lasso_RMSE_block(fold) = sqrt(mean((Z_test - Z_pred_lasso).^2));

X_search_local = linspace(min(X_train), max(X_train), 100);

Y_search_local = linspace(min(Y_train), max(Y_train), 100);

max_Z_val = -inf;

for i = 1:length(X_search_local)

for j = 1:length(Y_search_local)

Z_val = intercept + coef(1)*X_search_local(i) + coef(2)*Y_search_local(j) + ...

coef(3)*X_search_local(i)^2 + coef(4)*Y_search_local(j)^2 + ...

coef(5)*X_search_local(i)*Y_search_local(j);

if Z_val > max_Z_val

max_Z_val = Z_val;

best_X_lasso_block(fold) = X_search_local(i);

best_Y_lasso_block(fold) = Y_search_local(j);

end

end

end

% ======= Method 3: Random Forest =======

rf_model = TreeBagger(50, [X_train, Y_train], Z_train, 'Method', 'regression');

Z_pred_rf = predict(rf_model, [X_test, Y_test]);

SS_res = sum((Z_test - Z_pred_rf).^2);

rf_R2_block(fold) = 1 - SS_res/SS_tot;

rf_RMSE_block(fold) = sqrt(mean((Z_test - Z_pred_rf).^2));

X_search_local = linspace(min(X_train), max(X_train), 100);

Y_search_local = linspace(min(Y_train), max(Y_train), 100);

max_Z_val = -inf;

for i = 1:length(X_search_local)

for j = 1:length(Y_search_local)

Z_val = predict(rf_model, [X_search_local(i), Y_search_local(j)]);

if Z_val > max_Z_val && ~isnan(Z_val)

max_Z_val = Z_val;

best_X_rf_block(fold) = X_search_local(i);

best_Y_rf_block(fold) = Y_search_local(j);

end

end

end

% ======= Method 4: Neural Network =======

X_min_train = min(X_train); X_max_train = max(X_train);

Y_min_train = min(Y_train); Y_max_train = max(Y_train);

Z_min_train = min(Z_train); Z_max_train = max(Z_train);

X_norm_train = (X_train - X_min_train) / (X_max_train - X_min_train);

Y_norm_train = (Y_train - Y_min_train) / (Y_max_train - Y_min_train);

Z_norm_train = (Z_train - Z_min_train) / (Z_max_train - Z_min_train);

rng(fold * 100 + 42);

net = feedforwardnet(5);

net.trainFcn = 'trainlm';

net.trainParam.epochs = 200;

net.trainParam.showWindow = false;

inputs_train = [X_norm_train'; Y_norm_train'];

targets_train = Z_norm_train';

[net_trained, ~] = train(net, inputs_train, targets_train);

X_norm_test = (X_test - X_min_train) / (X_max_train - X_min_train);

Y_norm_test = (Y_test - Y_min_train) / (Y_max_train - Y_min_train);

inputs_test = [X_norm_test'; Y_norm_test'];

Z_norm_pred = net_trained(inputs_test);

Z_pred_nn = Z_norm_pred' * (Z_max_train - Z_min_train) + Z_min_train;

SS_res = sum((Z_test - Z_pred_nn).^2);

nn_R2_block(fold) = 1 - SS_res/SS_tot;

nn_RMSE_block(fold) = sqrt(mean((Z_test - Z_pred_nn).^2));

X_search_local = linspace(min(X_train), max(X_train), 100);

Y_search_local = linspace(min(Y_train), max(Y_train), 100);

max_Z_val = -inf;

for i = 1:length(X_search_local)

for j = 1:length(Y_search_local)

X_norm_val = (X_search_local(i) - X_min_train) / (X_max_train - X_min_train);

Y_norm_val = (Y_search_local(j) - Y_min_train) / (Y_max_train - Y_min_train);

Z_pred_val = net_trained([X_norm_val; Y_norm_val]) * ...

(Z_max_train - Z_min_train) + Z_min_train;

if Z_pred_val > max_Z_val

max_Z_val = Z_pred_val;

best_X_nn_block(fold) = X_search_local(i);

best_Y_nn_block(fold) = Y_search_local(j);

end

end

end

fprintf('QR: R²=%.4f, RMSE=%.4f, Optimal X=%.4f\n', ...

quadratic_R2_block(fold), quadratic_RMSE_block(fold), best_X_quadratic_block(fold));

fprintf('Lasso: R²=%.4f, RMSE=%.4f, Optimal X=%.4f\n', ...

lasso_R2_block(fold), lasso_RMSE_block(fold), best_X_lasso_block(fold));

fprintf('RF: R²=%.4f, RMSE=%.4f, Optimal X=%.4f\n', ...

rf_R2_block(fold), rf_RMSE_block(fold), best_X_rf_block(fold));

fprintf('NN: R²=%.4f, RMSE=%.4f, Optimal X=%.4f\n', ...

nn_R2_block(fold), nn_RMSE_block(fold), best_X_nn_block(fold));

end

% ==================== 3.6 Spatial Block Cross-Validation ====================

fprintf('\n\n=== Spatial Block Cross-Validation (Spatial Block CV) ===\n');

fprintf('Description: Data is divided into 5 consecutive spatial blocks by the value range of solid density X\n');

fprintf('Purpose: Control the impact of spatial proximity on performance estimation\n');

% Sort by X value and partition into blocks

[X_sorted, sort_idx_X] = sort(X);

block_size = floor(n / K);

indices_spatial = zeros(n, 1);

for fold = 1:K

start_idx = (fold-1) * block_size + 1;

end_idx = min(fold * block_size, n);

for i = start_idx:end_idx

indices_spatial(sort_idx_X(i)) = fold;

end

end

if end_idx < n

for i = end_idx+1:n

indices_spatial(sort_idx_X(i)) = K;

end

end

fprintf('Solid density X ranges per fold (partitioned by value range):\n');

for fold = 1:K

idx_range = find(indices_spatial == fold);

x_min = min(X(idx_range));

x_max = max(X(idx_range));

fprintf(' Fold %d: X ∈ [%.4f, %.4f] (%d samples)\n', fold, x_min, x_max, length(idx_range));

end

% Storage for spatial block CV results

quadratic_R2_spatial = zeros(K, 1);

quadratic_RMSE_spatial = zeros(K, 1);

lasso_R2_spatial = zeros(K, 1);

lasso_RMSE_spatial = zeros(K, 1);

rf_R2_spatial = zeros(K, 1);

rf_RMSE_spatial = zeros(K, 1);

nn_R2_spatial = zeros(K, 1);

nn_RMSE_spatial = zeros(K, 1);

best_X_quadratic_spatial = zeros(K, 1);

best_X_lasso_spatial = zeros(K, 1);

best_X_rf_spatial = zeros(K, 1);

best_X_nn_spatial = zeros(K, 1);

best_Y_quadratic_spatial = zeros(K, 1);

best_Y_lasso_spatial = zeros(K, 1);

best_Y_rf_spatial = zeros(K, 1);

best_Y_nn_spatial = zeros(K, 1);

for fold = 1:K

fprintf('\n--- Fold %d Spatial Block Cross-Validation ---\n', fold);

test_idx = (indices_spatial == fold);

train_idx = ~test_idx;

X_train = X(train_idx);

Y_train = Y(train_idx);

Z_train = Z(train_idx);

X_test = X(test_idx);

Y_test = Y(test_idx);

Z_test = Z(test_idx);

SS_tot = sum((Z_test - mean(Z_test)).^2);

% ======= Method 1: Quadratic Regression =======

A_train = [ones(size(X_train)), X_train, Y_train, ...

X_train.^2, Y_train.^2, X_train.*Y_train];

coefficients = A_train \ Z_train;

A_test = [ones(size(X_test)), X_test, Y_test, ...

X_test.^2, Y_test.^2, X_test.*Y_test];

Z_pred_quad = A_test * coefficients;

SS_res = sum((Z_test - Z_pred_quad).^2);

quadratic_R2_spatial(fold) = 1 - SS_res/SS_tot;

quadratic_RMSE_spatial(fold) = sqrt(mean((Z_test - Z_pred_quad).^2));

B0c = coefficients(1); B1c = coefficients(2); B2c = coefficients(3);

B3c = coefficients(4); B4c = coefficients(5); B5c = coefficients(6);

M = [2*B3c, B5c; B5c, 2*B4c];

RHS = [-B1c; -B2c];

if abs(det(M)) > 1e-10

XY_opt = M \ RHS;

best_X_quadratic_spatial(fold) = XY_opt(1);

best_Y_quadratic_spatial(fold) = XY_opt(2);

else

X_search_local = linspace(min(X_train), max(X_train), 100);

Y_search_local = linspace(min(Y_train), max(Y_train), 100);

max_Z_val = -inf;

for i = 1:length(X_search_local)

for j = 1:length(Y_search_local)

Z_val = B0c + B1c*X_search_local(i) + B2c*Y_search_local(j) + ...

B3c*X_search_local(i)^2 + B4c*Y_search_local(j)^2 + ...

B5c*X_search_local(i)*Y_search_local(j);

if Z_val > max_Z_val

max_Z_val = Z_val;

best_X_quadratic_spatial(fold) = X_search_local(i);

best_Y_quadratic_spatial(fold) = Y_search_local(j);

end

end

end

end

% ======= Method 2: Lasso Regression =======

A_train_lasso = [X_train, Y_train, X_train.^2, Y_train.^2, X_train.*Y_train];

A_test_lasso = [X_test, Y_test, X_test.^2, Y_test.^2, X_test.*Y_test];

[B, info] = lasso(A_train_lasso, Z_train, 'CV', 5);

idx = info.IndexMinMSE;

intercept = info.Intercept(idx);

coef = B(:, idx);

Z_pred_lasso = intercept + A_test_lasso * coef;

SS_res = sum((Z_test - Z_pred_lasso).^2);

lasso_R2_spatial(fold) = 1 - SS_res/SS_tot;

lasso_RMSE_spatial(fold) = sqrt(mean((Z_test - Z_pred_lasso).^2));

X_search_local = linspace(min(X_train), max(X_train), 100);

Y_search_local = linspace(min(Y_train), max(Y_train), 100);

max_Z_val = -inf;

for i = 1:length(X_search_local)

for j = 1:length(Y_search_local)

Z_val = intercept + coef(1)*X_search_local(i) + coef(2)*Y_search_local(j) + ...

coef(3)*X_search_local(i)^2 + coef(4)*Y_search_local(j)^2 + ...

coef(5)*X_search_local(i)*Y_search_local(j);

if Z_val > max_Z_val

max_Z_val = Z_val;

best_X_lasso_spatial(fold) = X_search_local(i);

best_Y_lasso_spatial(fold) = Y_search_local(j);

end

end

end

% ======= Method 3: Random Forest =======

rf_model = TreeBagger(50, [X_train, Y_train], Z_train, 'Method', 'regression');

Z_pred_rf = predict(rf_model, [X_test, Y_test]);

SS_res = sum((Z_test - Z_pred_rf).^2);

rf_R2_spatial(fold) = 1 - SS_res/SS_tot;

rf_RMSE_spatial(fold) = sqrt(mean((Z_test - Z_pred_rf).^2));

X_search_local = linspace(min(X_train), max(X_train), 100);

Y_search_local = linspace(min(Y_train), max(Y_train), 100);

max_Z_val = -inf;

for i = 1:length(X_search_local)

for j = 1:length(Y_search_local)

Z_val = predict(rf_model, [X_search_local(i), Y_search_local(j)]);

if Z_val > max_Z_val && ~isnan(Z_val)

max_Z_val = Z_val;

best_X_rf_spatial(fold) = X_search_local(i);

best_Y_rf_spatial(fold) = Y_search_local(j);

end

end

end

% ======= Method 4: Neural Network =======

X_min_train = min(X_train); X_max_train = max(X_train);

Y_min_train = min(Y_train); Y_max_train = max(Y_train);

Z_min_train = min(Z_train); Z_max_train = max(Z_train);

X_norm_train = (X_train - X_min_train) / (X_max_train - X_min_train);

Y_norm_train = (Y_train - Y_min_train) / (Y_max_train - Y_min_train);

Z_norm_train = (Z_train - Z_min_train) / (Z_max_train - Z_min_train);

rng(fold * 100 + 84);

net = feedforwardnet(5);

net.trainFcn = 'trainlm';

net.trainParam.epochs = 200;

net.trainParam.showWindow = false;

inputs_train = [X_norm_train'; Y_norm_train'];

targets_train = Z_norm_train';

[net_trained, ~] = train(net, inputs_train, targets_train);

X_norm_test = (X_test - X_min_train) / (X_max_train - X_min_train);

Y_norm_test = (Y_test - Y_min_train) / (Y_max_train - Y_min_train);

inputs_test = [X_norm_test'; Y_norm_test'];

Z_norm_pred = net_trained(inputs_test);

Z_pred_nn = Z_norm_pred' * (Z_max_train - Z_min_train) + Z_min_train;

SS_res = sum((Z_test - Z_pred_nn).^2);

nn_R2_spatial(fold) = 1 - SS_res/SS_tot;

nn_RMSE_spatial(fold) = sqrt(mean((Z_test - Z_pred_nn).^2));

X_search_local = linspace(min(X_train), max(X_train), 100);

Y_search_local = linspace(min(Y_train), max(Y_train), 100);

max_Z_val = -inf;

for i = 1:length(X_search_local)

for j = 1:length(Y_search_local)

X_norm_val = (X_search_local(i) - X_min_train) / (X_max_train - X_min_train);

Y_norm_val = (Y_search_local(j) - Y_min_train) / (Y_max_train - Y_min_train);

Z_pred_val = net_trained([X_norm_val; Y_norm_val]) * ...

(Z_max_train - Z_min_train) + Z_min_train;

if Z_pred_val > max_Z_val

max_Z_val = Z_pred_val;

best_X_nn_spatial(fold) = X_search_local(i);

best_Y_nn_spatial(fold) = Y_search_local(j);

end

end

end

fprintf('QR: R²=%.4f, RMSE=%.4f, Optimal X=%.4f\n', ...

quadratic_R2_spatial(fold), quadratic_RMSE_spatial(fold), best_X_quadratic_spatial(fold));

fprintf('Lasso: R²=%.4f, RMSE=%.4f, Optimal X=%.4f\n', ...

lasso_R2_spatial(fold), lasso_RMSE_spatial(fold), best_X_lasso_spatial(fold));

fprintf('RF: R²=%.4f, RMSE=%.4f, Optimal X=%.4f\n', ...

rf_R2_spatial(fold), rf_RMSE_spatial(fold), best_X_rf_spatial(fold));

fprintf('NN: R²=%.4f, RMSE=%.4f, Optimal X=%.4f\n', ...

nn_R2_spatial(fold), nn_RMSE_spatial(fold), best_X_nn_spatial(fold));

end

% ==================== 4. Statistical Analysis of Results ====================

fprintf('\n\n=== Statistical Analysis of Four Methods (Random CV) ===\n');

fprintf('\n1. Fitting Performance Comparison (5-fold CV):\n');

fprintf('--------------------------------------------------------------------\n');

fprintf('Method	Mean R²	R² Std	Mean RMSE	RMSE Std\n');

fprintf('--------------------------------------------------------------------\n');

fprintf('Quadratic	%.4f	%.4f	%.4f	%.4f\n', ...

mean(quadratic_R2), std(quadratic_R2), mean(quadratic_RMSE), std(quadratic_RMSE));

fprintf('Lasso	%.4f	%.4f	%.4f	%.4f\n', ...

mean(lasso_R2), std(lasso_R2), mean(lasso_RMSE), std(lasso_RMSE));

fprintf('Random Forest	%.4f	%.4f	%.4f	%.4f\n', ...

mean(rf_R2), std(rf_R2), mean(rf_RMSE), std(rf_RMSE));

fprintf('Neural Network	%.4f	%.4f	%.4f	%.4f\n', ...

mean(nn_R2), std(nn_R2), mean(nn_RMSE), std(nn_RMSE));

fprintf('--------------------------------------------------------------------\n');

fprintf('\n2. Optimal Solid Density Stability Comparison (Random CV):\n');

fprintf('Quadratic optimal X: %.4f ± %.4f (CV=%.2f%%)\n', mean(best_X_quadratic), std(best_X_quadratic), std(best_X_quadratic)/mean(best_X_quadratic)*100);

fprintf('Lasso optimal X: %.4f ± %.4f (CV=%.2f%%)\n', mean(best_X_lasso), std(best_X_lasso), std(best_X_lasso)/mean(best_X_lasso)*100);

fprintf('Random Forest optimal X: %.4f ± %.4f (CV=%.2f%%)\n', mean(best_X_rf), std(best_X_rf), std(best_X_rf)/mean(best_X_rf)*100);

fprintf('Neural Network optimal X: %.4f ± %.4f (CV=%.2f%%)\n', mean(best_X_nn), std(best_X_nn), std(best_X_nn)/mean(best_X_nn)*100);

% ==================== 4.5 Three CV Strategy Results Comparison ====================

fprintf('\n\n=== Comparison of Three Cross-Validation Strategies ===\n');

fprintf('Description:\n');

fprintf(' - Random CV: Randomly shuffled folds (baseline)\n');

fprintf(' - Temporal Block CV: Consecutive order folds (controls time dependence)\n');

fprintf(' - Spatial Block CV: X-value range folds (controls spatial dependence)\n');

fprintf('====================================================================\n');

fprintf('Model	| Metric | Random CV	| Temporal Block	| Spatial Block	\n');

fprintf('====================================================================\n');

print_compare3 = @(name, metric, val_random, val_block, val_spatial) ...

fprintf('%s	| %s	| %.4f ± %.4f | %.4f ± %.4f | %.4f ± %.4f\n', ...

name, metric, mean(val_random), std(val_random), mean(val_block), std(val_block), mean(val_spatial), std(val_spatial));

print_compare3('QR', 'R²', quadratic_R2, quadratic_R2_block, quadratic_R2_spatial);

print_compare3('Lasso', 'R²', lasso_R2, lasso_R2_block, lasso_R2_spatial);

print_compare3('RF', 'R²', rf_R2, rf_R2_block, rf_R2_spatial);

print_compare3('NN', 'R²', nn_R2, nn_R2_block, nn_R2_spatial);

print_compare3('QR', 'RMSE', quadratic_RMSE, quadratic_RMSE_block, quadratic_RMSE_spatial);

print_compare3('Lasso', 'RMSE', lasso_RMSE, lasso_RMSE_block, lasso_RMSE_spatial);

print_compare3('RF', 'RMSE', rf_RMSE, rf_RMSE_block, rf_RMSE_spatial);

print_compare3('NN', 'RMSE', nn_RMSE, nn_RMSE_block, nn_RMSE_spatial);

fprintf('====================================================================\n');

fprintf('\nModel ranking by strategy (by R²):\n');

fprintf(' Random CV: RF=%.4f, NN=%.4f, QR=%.4f, Lasso=%.4f\n', ...

mean(rf_R2), mean(nn_R2), mean(quadratic_R2), mean(lasso_R2));

fprintf(' Temporal CV: RF=%.4f, NN=%.4f, QR=%.4f, Lasso=%.4f\n', ...

mean(rf_R2_block), mean(nn_R2_block), mean(quadratic_R2_block), mean(lasso_R2_block));

fprintf(' Spatial CV: RF=%.4f, NN=%.4f, QR=%.4f, Lasso=%.4f\n', ...

mean(rf_R2_spatial), mean(nn_R2_spatial), mean(quadratic_R2_spatial), mean(lasso_R2_spatial));

% Generate bar chart comparing three CV strategies

figure('Position', [100, 100, 900, 600]);

bar_data = [mean(quadratic_R2), mean(lasso_R2), mean(rf_R2), mean(nn_R2);

mean(quadratic_R2_block), mean(lasso_R2_block), mean(rf_R2_block), mean(nn_R2_block);

mean(quadratic_R2_spatial), mean(lasso_R2_spatial), mean(rf_R2_spatial), mean(nn_R2_spatial)];

bar(bar_data);

set(gca, 'XTickLabel', {'QR', 'Lasso', 'RF', 'NN'});

legend('Random CV', 'Temporal Block CV', 'Spatial Block CV', 'Location', 'best');

ylabel('Mean R²');

title('Black Ink: R² Comparison Across Three CV Strategies');

grid on;

saveas(gcf, 'Black_Ink_CV_Comparison_Three.png');

fprintf('\nThree-strategy comparison figure saved: Black_Ink_CV_Comparison_Three.png\n');

% ==================== 5. Statistical Significance Test (Paired t-test) ====================

fprintf('\n=== Statistical Significance Test (Paired t-test) - Based on Random CV ===\n');

diff_R2_NN_QR = nn_R2 - quadratic_R2;

diff_R2_RF_QR = rf_R2 - quadratic_R2;

diff_R2_RF_NN = rf_R2 - nn_R2;

diff_R2_Lasso_QR = lasso_R2 - quadratic_R2;

[~, p_NN_QR] = ttest(diff_R2_NN_QR);

fprintf('\nNN vs QR:\n');

fprintf(' Mean R² difference: +%.4f\n', mean(diff_R2_NN_QR));

fprintf(' p = %.4f', p_NN_QR);

if p_NN_QR < 0.05

fprintf(' (Significant difference)\n');

else

fprintf(' (No significant difference)\n');

end

[~, p_RF_QR] = ttest(diff_R2_RF_QR);

fprintf('\nRF vs QR:\n');

fprintf(' Mean R² difference: +%.4f\n', mean(diff_R2_RF_QR));

fprintf(' p = %.4f', p_RF_QR);

if p_RF_QR < 0.05

fprintf(' (Significant difference)\n');

else

fprintf(' (No significant difference)\n');

end

[~, p_RF_NN] = ttest(diff_R2_RF_NN);

fprintf('\nRF vs NN:\n');

fprintf(' Mean R² difference: +%.4f\n', mean(diff_R2_RF_NN));

fprintf(' p = %.4f', p_RF_NN);

if p_RF_NN < 0.05

fprintf(' (Significant difference)\n');

else

fprintf(' (No significant difference)\n');

end

fprintf('\nLasso vs QR:\n');

fprintf(' Mean R² difference: +%.4f (No significant difference, p > 0.05)\n', mean(diff_R2_Lasso_QR));

% ==================== 6. Response Surface Analysis ====================

fprintf('\n=== Generating Response Surfaces ===\n');

xg = linspace(min(X), max(X), 50);

yg = linspace(min(Y), max(Y), 50);

[Xg, Yg] = meshgrid(xg, yg);

in_range = (Xg >= min(X)) & (Xg <= max(X)) & ...

(Yg >= min(Y)) & (Yg <= max(Y));

% 6.1 QR response surface

Zg_qr = B0 + B1*Xg + B2*Yg + B3*Xg.^2 + B4*Yg.^2 + B5*Xg.*Yg;

Zg_qr(~in_range) = NaN;

% 6.2 Lasso response surface

Zg_lasso = intercept_lasso + coef_lasso(1)*Xg + coef_lasso(2)*Yg + ...

coef_lasso(3)*Xg.^2 + coef_lasso(4)*Yg.^2 + coef_lasso(5)*Xg.*Yg;

Zg_lasso(~in_range) = NaN;

% 6.3 RF response surface

Zg_rf = predict(rf_full, [Xg(:), Yg(:)]);

Zg_rf = reshape(Zg_rf, size(Xg));

Zg_rf(~in_range) = NaN;

% 6.4 NN response surface

Xg_norm = (Xg - X_min) / (X_max - X_min);

Yg_norm = (Yg - Y_min) / (Y_max - Y_min);

Zg_nn = net_full([Xg_norm(:)'; Yg_norm(:)'])';

Zg_nn = reshape(Zg_nn, size(Xg)) * (Z_max - Z_min) + Z_min;

Zg_nn(~in_range) = NaN;

% Generate 2×2 comparison figure

figure('Position', [100, 100, 1200, 1000], 'Name', 'Black Ink: Four Model Response Surface Comparison');

subplot(2,2,1);

surf(Xg, Yg, Zg_qr, 'EdgeColor', 'none', 'FaceAlpha', 0.8);

hold on;

scatter3(X, Y, Z, 20, 'k', 'filled', 'MarkerFaceAlpha', 0.3);

title(sprintf('(a) Quadratic Regression QR (R²=%.4f)', R2_quad_full));

xlabel('Solid Density X'); ylabel('Dot Gain Y'); zlabel('Contrast Z');

colormap(jet); colorbar; view(45, 30); grid on;

subplot(2,2,2);

surf(Xg, Yg, Zg_lasso, 'EdgeColor', 'none', 'FaceAlpha', 0.8);

hold on;

scatter3(X, Y, Z, 20, 'k', 'filled', 'MarkerFaceAlpha', 0.3);

title(sprintf('(b) Lasso Regression (R²=%.4f)', R2_lasso_full));

xlabel('Solid Density X'); ylabel('Dot Gain Y'); zlabel('Contrast Z');

colormap(jet); colorbar; view(45, 30); grid on;

subplot(2,2,3);

surf(Xg, Yg, Zg_rf, 'EdgeColor', 'none', 'FaceAlpha', 0.8);

hold on;

scatter3(X, Y, Z, 20, 'k', 'filled', 'MarkerFaceAlpha', 0.3);

title(sprintf('(c) Random Forest RF (R²=%.4f)', R2_rf_full));

xlabel('Solid Density X'); ylabel('Dot Gain Y'); zlabel('Contrast Z');

colormap(jet); colorbar; view(45, 30); grid on;

subplot(2,2,4);

surf(Xg, Yg, Zg_nn, 'EdgeColor', 'none', 'FaceAlpha', 0.8);

hold on;

scatter3(X, Y, Z, 20, 'k', 'filled', 'MarkerFaceAlpha', 0.3);

title(sprintf('(d) Neural Network MLP (R²=%.4f)', R2_nn_full));

xlabel('Solid Density X'); ylabel('Dot Gain Y'); zlabel('Contrast Z');

colormap(jet); colorbar; view(45, 30); grid on;

sgtitle('Black Ink: Four Model Response Surface Comparison');

saveas(gcf, 'Black_Ink_ResponseSurfaces.png');

fprintf(' Response surface figure saved: Black_Ink_ResponseSurfaces.png\n');

% ==================== 7. Cross-Validation Analysis ====================

fprintf('\n=== Optimal Point Cross-Validation Analysis ===\n');

Z_nn_at_qr = interp2(Xg, Yg, Zg_nn, X_opt_quad, Y_opt_quad);

Z_rf_at_qr = interp2(Xg, Yg, Zg_rf, X_opt_quad, Y_opt_quad);

Z_lasso_at_qr = interp2(Xg, Yg, Zg_lasso, X_opt_quad, Y_opt_quad);

Z_qr_at_nn = interp2(Xg, Yg, Zg_qr, best_X_nn_full, best_Y_nn_full);

Z_rf_at_nn = interp2(Xg, Yg, Zg_rf, best_X_nn_full, best_Y_nn_full);

Z_lasso_at_nn = interp2(Xg, Yg, Zg_lasso, best_X_nn_full, best_Y_nn_full);

Z_qr_at_rf = interp2(Xg, Yg, Zg_qr, best_X_rf_full, best_Y_rf_full);

Z_nn_at_rf = interp2(Xg, Yg, Zg_nn, best_X_rf_full, best_Y_rf_full);

Z_lasso_at_rf = interp2(Xg, Yg, Zg_lasso, best_X_rf_full, best_Y_rf_full);

fprintf('\nQR optimum (X=%.4f, Y=%.4f, Z=%.4f):\n', X_opt_quad, Y_opt_quad, Z_opt_quad);

fprintf(' Prediction on NN: Z = %.4f (Diff: %+.4f)\n', Z_nn_at_qr, Z_nn_at_qr - Z_opt_quad);

fprintf(' Prediction on RF: Z = %.4f (Diff: %+.4f)\n', Z_rf_at_qr, Z_rf_at_qr - Z_opt_quad);

fprintf(' Prediction on Lasso: Z = %.4f (Diff: %+.4f)\n', Z_lasso_at_qr, Z_lasso_at_qr - Z_opt_quad);

fprintf('\nNN optimum (X=%.4f, Y=%.4f, Z=%.4f):\n', best_X_nn_full, best_Y_nn_full, max_Z_nn);

fprintf(' Prediction on QR: Z = %.4f (Diff: %+.4f)\n', Z_qr_at_nn, Z_qr_at_nn - max_Z_nn);

fprintf(' Prediction on RF: Z = %.4f (Diff: %+.4f)\n', Z_rf_at_nn, Z_rf_at_nn - max_Z_nn);

fprintf(' Prediction on Lasso: Z = %.4f (Diff: %+.4f)\n', Z_lasso_at_nn, Z_lasso_at_nn - max_Z_nn);

fprintf('\nRF optimum (X=%.4f, Y=%.4f, Z=%.4f):\n', best_X_rf_full, best_Y_rf_full, max_Z_rf);

fprintf(' Prediction on QR: Z = %.4f (Diff: %+.4f)\n', Z_qr_at_rf, Z_qr_at_rf - max_Z_rf);

fprintf(' Prediction on NN: Z = %.4f (Diff: %+.4f)\n', Z_nn_at_rf, Z_nn_at_rf - max_Z_rf);

fprintf(' Prediction on Lasso: Z = %.4f (Diff: %+.4f)\n', Z_lasso_at_rf, Z_lasso_at_rf - max_Z_rf);

% ==================== 8. Residual Analysis ====================

fprintf('\n=== Residual Analysis ===\n');

residuals_qr = Z - Z_pred_quad_full;

residuals_lasso = Z - Z_pred_lasso_full;

residuals_rf = Z - Z_pred_rf_full;

residuals_nn = Z - Z_pred_nn_full;

figure('Position', [100, 100, 800, 800], 'Name', 'Residual Analysis');

subplot(2,2,1);

scatter(Z_pred_quad_full, residuals_qr, 'b.');

hold on;

plot([min(Z_pred_quad_full), max(Z_pred_quad_full)], [0, 0], 'r--', 'LineWidth', 1);

xlabel('Predicted Value'); ylabel('Residual'); title('(a) QR Residual Plot');

grid on;

subplot(2,2,2);

scatter(Z_pred_lasso_full, residuals_lasso, 'g.');

hold on;

plot([min(Z_pred_lasso_full), max(Z_pred_lasso_full)], [0, 0], 'r--', 'LineWidth', 1);

xlabel('Predicted Value'); ylabel('Residual'); title('(b) Lasso Residual Plot');

grid on;

subplot(2,2,3);

scatter(Z_pred_rf_full, residuals_rf, 'r.');

hold on;

plot([min(Z_pred_rf_full), max(Z_pred_rf_full)], [0, 0], 'r--', 'LineWidth', 1);

xlabel('Predicted Value'); ylabel('Residual'); title('(c) RF Residual Plot');

grid on;

subplot(2,2,4);

scatter(Z_pred_nn_full, residuals_nn, 'm.');

hold on;

plot([min(Z_pred_nn_full), max(Z_pred_nn_full)], [0, 0], 'r--', 'LineWidth', 1);

xlabel('Predicted Value'); ylabel('Residual'); title('(d) NN Residual Plot');

grid on;

sgtitle('Four Model Residual Distribution Comparison');

saveas(gcf, 'Black_Ink_Residuals.png');

% ==================== 9. Generate Comparison Charts ====================

fprintf('\n=== Generating Comparison Charts ===\n');

% Figure 1: R² boxplot

figure('Position', [100, 100, 600, 500], 'Name', 'R² Boxplot');

boxplot([quadratic_R2, lasso_R2, rf_R2, nn_R2], 'Labels', {'QR', 'Lasso', 'RF', 'NN'});

ylabel('Coefficient of Determination R²');

title('Black Ink: R² Distribution Comparison (Random 5-fold CV)');

grid on;

saveas(gcf, 'Black_Ink_R2_Boxplot.png');

% Figure 2: RMSE boxplot

figure('Position', [150, 150, 600, 500], 'Name', 'RMSE Boxplot');

boxplot([quadratic_RMSE, lasso_RMSE, rf_RMSE, nn_RMSE], 'Labels', {'QR', 'Lasso', 'RF', 'NN'});

ylabel('Root Mean Square Error RMSE');

title('Black Ink: RMSE Distribution Comparison (Random 5-fold CV)');

grid on;

saveas(gcf, 'Black_Ink_RMSE_Boxplot.png');

% Figure 3: Optimal X stability line chart (Random CV)

figure('Position', [200, 200, 700, 500], 'Name', 'Optimal X Stability');

hold on;

plot(1:K, best_X_quadratic, 'bo-', 'LineWidth', 2, 'MarkerSize', 8, 'DisplayName', 'QR');

plot(1:K, best_X_lasso, 'go-', 'LineWidth', 2, 'MarkerSize', 8, 'DisplayName', 'Lasso');

plot(1:K, best_X_rf, 'ro-', 'LineWidth', 2, 'MarkerSize', 8, 'DisplayName', 'RF');

plot(1:K, best_X_nn, 'mo-', 'LineWidth', 2, 'MarkerSize', 8, 'DisplayName', 'NN');

xlabel('Cross-Validation Fold'); ylabel('Optimal Solid Density X');

title('Black Ink: Optimal Solid Density Variation Across Folds (Random CV)');

legend('Location', 'best'); grid on;

xlim([0.5, 5.5]);

saveas(gcf, 'Black_Ink_Xopt_Stability.png');

% Figure 4: Mean performance bar chart

figure('Position', [250, 250, 800, 500], 'Name', 'Mean Performance Comparison');

subplot(1,2,1);

mean_R2 = [mean(quadratic_R2), mean(lasso_R2), mean(rf_R2), mean(nn_R2)];

std_R2 = [std(quadratic_R2), std(lasso_R2), std(rf_R2), std(nn_R2)];

bar(1:4, mean_R2, 0.6, 'FaceColor', [0.5 0.5 0.8]);

hold on;

errorbar(1:4, mean_R2, std_R2, 'k.', 'LineWidth', 1.5, 'MarkerSize', 15);

set(gca, 'XTickLabel', {'QR', 'Lasso', 'RF', 'NN'});

ylabel('Mean R²'); title('Black Ink: Mean R² Comparison');

ylim([0.8, 1.0]); grid on;

subplot(1,2,2);

mean_RMSE = [mean(quadratic_RMSE), mean(lasso_RMSE), mean(rf_RMSE), mean(nn_RMSE)];

std_RMSE = [std(quadratic_RMSE), std(lasso_RMSE), std(rf_RMSE), std(nn_RMSE)];

bar(1:4, mean_RMSE, 0.6, 'FaceColor', [0.8 0.5 0.5]);

hold on;

errorbar(1:4, mean_RMSE, std_RMSE, 'k.', 'LineWidth', 1.5, 'MarkerSize', 15);

set(gca, 'XTickLabel', {'QR', 'Lasso', 'RF', 'NN'});

ylabel('Mean RMSE'); title('Black Ink: Mean RMSE Comparison');

grid on;

sgtitle('Black Ink: Four Model Mean Performance Comparison (Random 5-fold CV)');

saveas(gcf, 'Black_Ink_Performance_Bar.png');

fprintf('\n=== Analysis Complete! ===\n');

fprintf('Generated image files:\n');

fprintf(' Black_Ink_Autocorrelation.png\n');

fprintf(' Black_Ink_CV_Comparison_Three.png <- New: Three CV strategy comparison\n');

fprintf(' Black_Ink_ResponseSurfaces.png\n');

fprintf(' Black_Ink_Residuals.png\n');

fprintf(' Black_Ink_R2_Boxplot.png\n');

fprintf(' Black_Ink_RMSE_Boxplot.png\n');

fprintf(' Black_Ink_Xopt_Stability.png\n');

fprintf(' Black_Ink_Performance_Bar.png\n');

## ## 2. Cyan Ink

% ==================== 1. Data Preparation ====================

fprintf('=== Cyan Ink Data Preparation ===\n');

DATA = [

1.05	10.12	20.59

1.05	10.12	20.48

1.05	10.12	20.22

1.05	10.12	20.18

1.05	10.12	20.94

1.05	10.12	20.98

1.16	11.23	20.86

1.16	11.23	21.09

1.16	11.23	21.11

1.16	11.23	21.19

1.16	11.23	23.18

1.16	11.23	23.15

1.19	12.17	23.72

1.19	12.17	25.13

1.19	12.17	25.69

1.19	12.17	25.71

1.19	12.17	25.62

1.19	12.17	26.70

1.22	12.85	25.89

1.22	12.85	26.65

1.22	12.85	26.61

1.22	12.85	26.65

1.22	12.85	26.64

1.22	12.85	26.66

1.24	13.25	30.15

1.24	13.25	30.13

1.24	13.25	30.15

1.24	13.25	30.14

1.24	13.25	30.16

1.24	13.25	30.12

1.27	13.88	31.15

1.27	13.88	31.13

1.27	13.88	31.13

1.27	13.88	31.51

1.27	13.88	31.12

1.27	13.88	31.15

1.28	14.32	31.59

1.28	14.32	31.14

1.28	14.32	31.47

1.28	14.32	31.52

1.28	14.32	31.51

1.28	14.32	31.61

1.32	14.62	33.56

1.32	14.62	33.56

1.32	14.62	33.56

1.32	14.62	33.56

1.32	14.62	33.56

1.32	14.62	33.56

1.35	15.14	34.93

1.35	15.14	34.93

1.35	15.14	34.93

1.35	15.14	34.93

1.35	15.14	34.93

1.35	15.14	34.93

1.37	15.31	36.76

1.37	15.31	36.76

1.37	15.31	36.76

1.37	15.31	36.76

1.37	15.31	36.76

1.37	15.31	36.76

1.39	15.75	39.45

1.39	15.75	39.45

1.39	15.75	39.45

1.39	15.75	39.45

1.39	15.75	39.45

1.39	15.75	39.45

1.42	15.95	41.69

1.42	15.95	41.69

1.42	15.95	41.69

1.42	15.95	41.69

1.42	15.95	41.69

1.42	15.95	41.69

1.45	16.35	43.71

1.45	16.35	43.71

1.45	16.35	43.71

1.45	16.35	43.71

1.45	16.35	43.71

1.45	16.35	43.71

1.46	16.54	40.83

1.46	16.54	40.83

1.46	16.54	40.83

1.46	16.54	40.83

1.46	16.54	40.83

1.46	16.54	40.83

1.49	17.08	37.18

1.49	17.08	37.18

1.49	17.08	37.18

1.49	17.08	37.18

1.49	17.08	37.18

1.49	17.08	37.18

1.52	17.58	34.36

1.52	17.58	34.36

1.52	17.58	34.36

1.52	17.58	34.36

1.52	17.58	34.36

1.52	17.58	34.36

1.54	18.13	32.77

1.54	18.13	32.77

1.54	18.13	32.77

1.54	18.13	32.77

1.54	18.13	32.77

1.54	18.13	32.77

1.57	18.75	30.39

1.57	18.75	30.39

1.57	18.75	30.39

1.57	18.75	30.39

1.63	18.75	27.83

1.63	18.75	27.83

1.63	19.32	27.83

1.63	19.32	27.83

];

X = DATA(:, 1);

Y = DATA(:, 2);

Z = DATA(:, 3);

n = length(X);

fprintf('Sample size: %d\n', n);

fprintf('Solid density range: %.2f - %.2f\n', min(X), max(X));

fprintf('Dot gain range: %.2f - %.2f\n', min(Y), max(Y));

fprintf('Contrast range: %.2f - %.2f\n\n', min(Z), max(Z));

% ==================== 1.5 Autocorrelation Analysis ====================

fprintf('\n=== Autocorrelation Analysis ===\n');

% Manual autocorrelation calculation

max_lag = 20;

Z_mean = mean(Z);

Z_var = var(Z);

acf_manual = zeros(max_lag + 1, 1);

for lag = 0:max_lag

if lag == 0

acf_manual(lag+1) = 1;

else

sum_cov = 0;

for i = 1:(n-lag)

sum_cov = sum_cov + (Z(i) - Z_mean) * (Z(i+lag) - Z_mean);

end

acf_manual(lag+1) = sum_cov / ((n-lag) * Z_var);

end

end

lags_manual = (0:max_lag)';

conf = 1.96 / sqrt(n);

figure('Position', [100, 100, 800, 500]);

stem(lags_manual, acf_manual, 'filled', 'LineWidth', 1.5);

hold on;

plot([0, max_lag], [conf, conf], 'r--', 'LineWidth', 1);

plot([0, max_lag], [-conf, -conf], 'r--', 'LineWidth', 1);

xlabel('Lag'); ylabel('Autocorrelation Coefficient');

title('Autocorrelation Function of Cyan Ink Contrast Values');

grid on;

saveas(gcf, 'Cyan_Ink_Autocorrelation.png');

fprintf('Lag-1 autocorrelation coefficient: %.4f\n', acf_manual(2));

% ==================== 2. Full-Data Analysis (Baseline) ====================

fprintf('=== Full-Data Baseline Analysis ===\n');

% 2.1 Quadratic regression full-data fitting

A_full = [ones(size(X)), X, Y, X.^2, Y.^2, X.*Y];

coeff_full = A_full \ Z;

Z_pred_quad_full = A_full * coeff_full;

R2_quad_full = 1 - sum((Z - Z_pred_quad_full).^2) / sum((Z - mean(Z)).^2);

B0 = coeff_full(1); B1 = coeff_full(2); B2 = coeff_full(3);

B3 = coeff_full(4); B4 = coeff_full(5); B5 = coeff_full(6);

M = [2*B3, B5; B5, 2*B4];

RHS = [-B1; -B2];

if abs(det(M)) > 1e-10

XY_opt_quad = M \ RHS;

X_opt_quad = XY_opt_quad(1);

Y_opt_quad = XY_opt_quad(2);

else

X_search = linspace(min(X), max(X), 100);

Y_search = linspace(min(Y), max(Y), 100);

max_Z = -inf;

for i = 1:length(X_search)

for j = 1:length(Y_search)

Z_val = B0 + B1*X_search(i) + B2*Y_search(j) + ...

B3*X_search(i)^2 + B4*Y_search(j)^2 + ...

B5*X_search(i)*Y_search(j);

if Z_val > max_Z

max_Z = Z_val;

X_opt_quad = X_search(i);

Y_opt_quad = Y_search(j);

end

end

end

end

Z_opt_quad = B0 + B1*X_opt_quad + B2*Y_opt_quad + B3*X_opt_quad^2 + ...

B4*Y_opt_quad^2 + B5*X_opt_quad*Y_opt_quad;

fprintf('\nQuadratic Regression (full data):\n');

fprintf(' R² = %.4f\n', R2_quad_full);

fprintf(' Optimal solid density X = %.4f\n', X_opt_quad);

fprintf(' Optimal dot gain Y = %.4f\n', Y_opt_quad);

fprintf(' Predicted maximum contrast Z = %.4f\n', Z_opt_quad);

% 2.2 Lasso full-data fitting

fprintf('\nLasso Regression (full data):\n');

A_lasso_full = [X, Y, X.^2, Y.^2, X.*Y];

[B_lasso, info_lasso] = lasso(A_lasso_full, Z, 'CV', 5);

idx_lasso = info_lasso.IndexMinMSE;

intercept_lasso = info_lasso.Intercept(idx_lasso);

coef_lasso = B_lasso(:, idx_lasso);

Z_pred_lasso_full = intercept_lasso + A_lasso_full * coef_lasso;

R2_lasso_full = 1 - sum((Z - Z_pred_lasso_full).^2) / sum((Z - mean(Z)).^2);

% Grid search for Lasso optimal point

X_search = linspace(1.3, 1.6, 100);

Y_search = linspace(16, 19, 100);

max_Z_lasso = -inf;

best_X_lasso_full = NaN;

best_Y_lasso_full = NaN;

for i = 1:length(X_search)

for j = 1:length(Y_search)

Z_val = intercept_lasso + coef_lasso(1)*X_search(i) + coef_lasso(2)*Y_search(j) + ...

coef_lasso(3)*X_search(i)^2 + coef_lasso(4)*Y_search(j)^2 + ...

coef_lasso(5)*X_search(i)*Y_search(j);

if Z_val > max_Z_lasso

max_Z_lasso = Z_val;

best_X_lasso_full = X_search(i);

best_Y_lasso_full = Y_search(j);

end

end

end

fprintf(' R² = %.4f\n', R2_lasso_full);

fprintf(' Optimal solid density X = %.4f\n', best_X_lasso_full);

fprintf(' Optimal dot gain Y = %.4f\n', best_Y_lasso_full);

fprintf(' Predicted maximum contrast Z = %.4f\n', max_Z_lasso);

% 2.3 Random forest full-data fitting

fprintf('\nRandom Forest (full data):\n');

rf_full = TreeBagger(50, [X, Y], Z, 'Method', 'regression');

Z_pred_rf_full = predict(rf_full, [X, Y]);

R2_rf_full = 1 - sum((Z - Z_pred_rf_full).^2) / sum((Z - mean(Z)).^2);

% Grid search for RF optimal point

X_search = linspace(1.3, 1.6, 100);

Y_search = linspace(16, 19, 100);

max_Z_rf = -inf;

best_X_rf_full = NaN;

best_Y_rf_full = NaN;

for i = 1:length(X_search)

for j = 1:length(Y_search)

Z_val = predict(rf_full, [X_search(i), Y_search(j)]);

if Z_val > max_Z_rf && ~isnan(Z_val)

max_Z_rf = Z_val;

best_X_rf_full = X_search(i);

best_Y_rf_full = Y_search(j);

end

end

end

fprintf(' R² = %.4f\n', R2_rf_full);

fprintf(' Optimal solid density X = %.4f\n', best_X_rf_full);

fprintf(' Optimal dot gain Y = %.4f\n', best_Y_rf_full);

fprintf(' Predicted maximum contrast Z = %.4f\n', max_Z_rf);

% 2.4 Neural network full-data fitting

fprintf('\nNeural Network (full data):\n');

X_min = min(X); X_max = max(X);

Y_min = min(Y); Y_max = max(Y);

Z_min = min(Z); Z_max = max(Z);

X_norm = (X - X_min) / (X_max - X_min);

Y_norm = (Y - Y_min) / (Y_max - Y_min);

Z_norm = (Z - Z_min) / (Z_max - Z_min);

rng(42);

net_full = feedforwardnet(5);

net_full.trainFcn = 'trainlm';

net_full.trainParam.epochs = 300;

net_full.trainParam.showWindow = false;

inputs_full = [X_norm'; Y_norm'];

targets_full = Z_norm';

[net_full, ~] = train(net_full, inputs_full, targets_full);

Z_pred_norm = net_full(inputs_full);

Z_pred_nn_full = Z_pred_norm' * (Z_max - Z_min) + Z_min;

R2_nn_full = 1 - sum((Z - Z_pred_nn_full).^2) / sum((Z - mean(Z)).^2);

X_search = linspace(1.3, 1.6, 100);

Y_search = linspace(16, 19, 100);

max_Z_nn = -inf;

best_X_nn_full = NaN;

best_Y_nn_full = NaN;

for i = 1:length(X_search)

for j = 1:length(Y_search)

X_norm_val = (X_search(i) - X_min) / (X_max - X_min);

Y_norm_val = (Y_search(j) - Y_min) / (Y_max - Y_min);

Z_pred_val = net_full([X_norm_val; Y_norm_val]) * (Z_max - Z_min) + Z_min;

if Z_pred_val > max_Z_nn

max_Z_nn = Z_pred_val;

best_X_nn_full = X_search(i);

best_Y_nn_full = Y_search(j);

end

end

end

fprintf(' R² = %.4f\n', R2_nn_full);

fprintf(' Optimal solid density X = %.4f\n', best_X_nn_full);

fprintf(' Optimal dot gain Y = %.4f\n', best_Y_nn_full);

fprintf(' Predicted maximum contrast Z = %.4f\n', max_Z_nn);

% ==================== 3. Random 5-Fold Cross-Validation ====================

fprintf('\n\n=== Random 5-Fold Cross-Validation Method Comparison ===\n');

K = 5;

rng(42);

indices = crossvalind('Kfold', n, K);

% Storage for 4 methods' results (random CV)

quadratic_R2 = zeros(K, 1);

quadratic_RMSE = zeros(K, 1);

lasso_R2 = zeros(K, 1);

lasso_RMSE = zeros(K, 1);

rf_R2 = zeros(K, 1);

rf_RMSE = zeros(K, 1);

nn_R2 = zeros(K, 1);

nn_RMSE = zeros(K, 1);

best_X_quadratic = zeros(K, 1);

best_X_lasso = zeros(K, 1);

best_X_rf = zeros(K, 1);

best_X_nn = zeros(K, 1);

best_Y_quadratic = zeros(K, 1);

best_Y_lasso = zeros(K, 1);

best_Y_rf = zeros(K, 1);

best_Y_nn = zeros(K, 1);

for fold = 1:K

fprintf('\n--- Fold %d Cross-Validation ---\n', fold);

test_idx = (indices == fold);

train_idx = ~test_idx;

X_train = X(train_idx);

Y_train = Y(train_idx);

Z_train = Z(train_idx);

X_test = X(test_idx);

Y_test = Y(test_idx);

Z_test = Z(test_idx);

SS_tot = sum((Z_test - mean(Z_test)).^2);

% ======= Method 1: Quadratic Regression =======

A_train = [ones(size(X_train)), X_train, Y_train, ...

X_train.^2, Y_train.^2, X_train.*Y_train];

coefficients = A_train \ Z_train;

A_test = [ones(size(X_test)), X_test, Y_test, ...

X_test.^2, Y_test.^2, X_test.*Y_test];

Z_pred_quad = A_test * coefficients;

SS_res = sum((Z_test - Z_pred_quad).^2);

quadratic_R2(fold) = 1 - SS_res/SS_tot;

quadratic_RMSE(fold) = sqrt(mean((Z_test - Z_pred_quad).^2));

B0c = coefficients(1); B1c = coefficients(2); B2c = coefficients(3);

B3c = coefficients(4); B4c = coefficients(5); B5c = coefficients(6);

M = [2*B3c, B5c; B5c, 2*B4c];

RHS = [-B1c; -B2c];

if abs(det(M)) > 1e-10

XY_opt = M \ RHS;

best_X_quadratic(fold) = XY_opt(1);

best_Y_quadratic(fold) = XY_opt(2);

else

X_search_local = linspace(min(X_train), max(X_train), 100);

Y_search_local = linspace(min(Y_train), max(Y_train), 100);

max_Z_val = -inf;

for i = 1:length(X_search_local)

for j = 1:length(Y_search_local)

Z_val = B0c + B1c*X_search_local(i) + B2c*Y_search_local(j) + ...

B3c*X_search_local(i)^2 + B4c*Y_search_local(j)^2 + ...

B5c*X_search_local(i)*Y_search_local(j);

if Z_val > max_Z_val

max_Z_val = Z_val;

best_X_quadratic(fold) = X_search_local(i);

best_Y_quadratic(fold) = Y_search_local(j);

end

end

end

end

% ======= Method 2: Lasso Regression =======

A_train_lasso = [X_train, Y_train, X_train.^2, Y_train.^2, X_train.*Y_train];

A_test_lasso = [X_test, Y_test, X_test.^2, Y_test.^2, X_test.*Y_test];

[B, info] = lasso(A_train_lasso, Z_train, 'CV', 5);

idx = info.IndexMinMSE;

intercept = info.Intercept(idx);

coef = B(:, idx);

Z_pred_lasso = intercept + A_test_lasso * coef;

SS_res = sum((Z_test - Z_pred_lasso).^2);

lasso_R2(fold) = 1 - SS_res/SS_tot;

lasso_RMSE(fold) = sqrt(mean((Z_test - Z_pred_lasso).^2));

X_search_local = linspace(min(X_train), max(X_train), 100);

Y_search_local = linspace(min(Y_train), max(Y_train), 100);

max_Z_val = -inf;

for i = 1:length(X_search_local)

for j = 1:length(Y_search_local)

Z_val = intercept + coef(1)*X_search_local(i) + coef(2)*Y_search_local(j) + ...

coef(3)*X_search_local(i)^2 + coef(4)*Y_search_local(j)^2 + ...

coef(5)*X_search_local(i)*Y_search_local(j);

if Z_val > max_Z_val

max_Z_val = Z_val;

best_X_lasso(fold) = X_search_local(i);

best_Y_lasso(fold) = Y_search_local(j);

end

end

end

% ======= Method 3: Random Forest =======

rf_model = TreeBagger(50, [X_train, Y_train], Z_train, 'Method', 'regression');

Z_pred_rf = predict(rf_model, [X_test, Y_test]);

SS_res = sum((Z_test - Z_pred_rf).^2);

rf_R2(fold) = 1 - SS_res/SS_tot;

rf_RMSE(fold) = sqrt(mean((Z_test - Z_pred_rf).^2));

X_search_local = linspace(min(X_train), max(X_train), 100);

Y_search_local = linspace(min(Y_train), max(Y_train), 100);

max_Z_val = -inf;

for i = 1:length(X_search_local)

for j = 1:length(Y_search_local)

Z_val = predict(rf_model, [X_search_local(i), Y_search_local(j)]);

if Z_val > max_Z_val && ~isnan(Z_val)

max_Z_val = Z_val;

best_X_rf(fold) = X_search_local(i);

best_Y_rf(fold) = Y_search_local(j);

end

end

end

% ======= Method 4: Neural Network =======

X_min_train = min(X_train); X_max_train = max(X_train);

Y_min_train = min(Y_train); Y_max_train = max(Y_train);

Z_min_train = min(Z_train); Z_max_train = max(Z_train);

X_norm_train = (X_train - X_min_train) / (X_max_train - X_min_train);

Y_norm_train = (Y_train - Y_min_train) / (Y_max_train - Y_min_train);

Z_norm_train = (Z_train - Z_min_train) / (Z_max_train - Z_min_train);

rng(fold * 100);

net = feedforwardnet(5);

net.trainFcn = 'trainlm';

net.trainParam.epochs = 200;

net.trainParam.showWindow = false;

inputs_train = [X_norm_train'; Y_norm_train'];

targets_train = Z_norm_train';

[net_trained, ~] = train(net, inputs_train, targets_train);

X_norm_test = (X_test - X_min_train) / (X_max_train - X_min_train);

Y_norm_test = (Y_test - Y_min_train) / (Y_max_train - Y_min_train);

inputs_test = [X_norm_test'; Y_norm_test'];

Z_norm_pred = net_trained(inputs_test);

Z_pred_nn = Z_norm_pred' * (Z_max_train - Z_min_train) + Z_min_train;

SS_res = sum((Z_test - Z_pred_nn).^2);

nn_R2(fold) = 1 - SS_res/SS_tot;

nn_RMSE(fold) = sqrt(mean((Z_test - Z_pred_nn).^2));

X_search_local = linspace(min(X_train), max(X_train), 100);

Y_search_local = linspace(min(Y_train), max(Y_train), 100);

max_Z_val = -inf;

for i = 1:length(X_search_local)

for j = 1:length(Y_search_local)

X_norm_val = (X_search_local(i) - X_min_train) / (X_max_train - X_min_train);

Y_norm_val = (Y_search_local(j) - Y_min_train) / (Y_max_train - Y_min_train);

Z_pred_val = net_trained([X_norm_val; Y_norm_val]) * ...

(Z_max_train - Z_min_train) + Z_min_train;

if Z_pred_val > max_Z_val

max_Z_val = Z_pred_val;

best_X_nn(fold) = X_search_local(i);

best_Y_nn(fold) = Y_search_local(j);

end

end

end

fprintf('QR: R²=%.4f, RMSE=%.4f, Optimal X=%.4f\n', ...

quadratic_R2(fold), quadratic_RMSE(fold), best_X_quadratic(fold));

fprintf('Lasso: R²=%.4f, RMSE=%.4f, Optimal X=%.4f\n', ...

lasso_R2(fold), lasso_RMSE(fold), best_X_lasso(fold));

fprintf('RF: R²=%.4f, RMSE=%.4f, Optimal X=%.4f\n', ...

rf_R2(fold), rf_RMSE(fold), best_X_rf(fold));

fprintf('NN: R²=%.4f, RMSE=%.4f, Optimal X=%.4f\n', ...

nn_R2(fold), nn_RMSE(fold), best_X_nn(fold));

end

% ==================== 3.5 Temporal Block Cross-Validation ====================

fprintf('\n\n=== Temporal Block Cross-Validation (Temporal Block CV) ===\n');

fprintf('Description: Data is divided into 5 consecutive blocks in collection order, each block as one fold\n');

fprintf('Purpose: Control the impact of time-series autocorrelation on performance estimation\n');

block_size = floor(n / K);

indices_block = zeros(n, 1);

for fold = 1:K

start_idx = (fold-1) * block_size + 1;

end_idx = min(fold * block_size, n);

indices_block(start_idx:end_idx) = fold;

end

if end_idx < n

indices_block(end_idx+1:end) = K;

end

fprintf('Sample ranges per fold (in collection order):\n');

for fold = 1:K

idx_range = find(indices_block == fold);

fprintf(' Fold %d: Samples %d - %d (%d samples)\n', fold, min(idx_range), max(idx_range), length(idx_range));

end

% Storage for temporal block CV results

quadratic_R2_block = zeros(K, 1);

quadratic_RMSE_block = zeros(K, 1);

lasso_R2_block = zeros(K, 1);

lasso_RMSE_block = zeros(K, 1);

rf_R2_block = zeros(K, 1);

rf_RMSE_block = zeros(K, 1);

nn_R2_block = zeros(K, 1);

nn_RMSE_block = zeros(K, 1);

best_X_quadratic_block = zeros(K, 1);

best_X_lasso_block = zeros(K, 1);

best_X_rf_block = zeros(K, 1);

best_X_nn_block = zeros(K, 1);

best_Y_quadratic_block = zeros(K, 1);

best_Y_lasso_block = zeros(K, 1);

best_Y_rf_block = zeros(K, 1);

best_Y_nn_block = zeros(K, 1);

for fold = 1:K

fprintf('\n--- Fold %d Temporal Block Cross-Validation ---\n', fold);

test_idx = (indices_block == fold);

train_idx = ~test_idx;

X_train = X(train_idx);

Y_train = Y(train_idx);

Z_train = Z(train_idx);

X_test = X(test_idx);

Y_test = Y(test_idx);

Z_test = Z(test_idx);

SS_tot = sum((Z_test - mean(Z_test)).^2);

% ======= Method 1: Quadratic Regression =======

A_train = [ones(size(X_train)), X_train, Y_train, ...

X_train.^2, Y_train.^2, X_train.*Y_train];

coefficients = A_train \ Z_train;

A_test = [ones(size(X_test)), X_test, Y_test, ...

X_test.^2, Y_test.^2, X_test.*Y_test];

Z_pred_quad = A_test * coefficients;

SS_res = sum((Z_test - Z_pred_quad).^2);

quadratic_R2_block(fold) = 1 - SS_res/SS_tot;

quadratic_RMSE_block(fold) = sqrt(mean((Z_test - Z_pred_quad).^2));

B0c = coefficients(1); B1c = coefficients(2); B2c = coefficients(3);

B3c = coefficients(4); B4c = coefficients(5); B5c = coefficients(6);

M = [2*B3c, B5c; B5c, 2*B4c];

RHS = [-B1c; -B2c];

if abs(det(M)) > 1e-10

XY_opt = M \ RHS;

best_X_quadratic_block(fold) = XY_opt(1);

best_Y_quadratic_block(fold) = XY_opt(2);

else

X_search_local = linspace(min(X_train), max(X_train), 100);

Y_search_local = linspace(min(Y_train), max(Y_train), 100);

max_Z_val = -inf;

for i = 1:length(X_search_local)

for j = 1:length(Y_search_local)

Z_val = B0c + B1c*X_search_local(i) + B2c*Y_search_local(j) + ...

B3c*X_search_local(i)^2 + B4c*Y_search_local(j)^2 + ...

B5c*X_search_local(i)*Y_search_local(j);

if Z_val > max_Z_val

max_Z_val = Z_val;

best_X_quadratic_block(fold) = X_search_local(i);

best_Y_quadratic_block(fold) = Y_search_local(j);

end

end

end

end

% ======= Method 2: Lasso Regression =======

A_train_lasso = [X_train, Y_train, X_train.^2, Y_train.^2, X_train.*Y_train];

A_test_lasso = [X_test, Y_test, X_test.^2, Y_test.^2, X_test.*Y_test];

[B, info] = lasso(A_train_lasso, Z_train, 'CV', 5);

idx = info.IndexMinMSE;

intercept = info.Intercept(idx);

coef = B(:, idx);

Z_pred_lasso = intercept + A_test_lasso * coef;

SS_res = sum((Z_test - Z_pred_lasso).^2);

lasso_R2_block(fold) = 1 - SS_res/SS_tot;

lasso_RMSE_block(fold) = sqrt(mean((Z_test - Z_pred_lasso).^2));

X_search_local = linspace(min(X_train), max(X_train), 100);

Y_search_local = linspace(min(Y_train), max(Y_train), 100);

max_Z_val = -inf;

for i = 1:length(X_search_local)

for j = 1:length(Y_search_local)

Z_val = intercept + coef(1)*X_search_local(i) + coef(2)*Y_search_local(j) + ...

coef(3)*X_search_local(i)^2 + coef(4)*Y_search_local(j)^2 + ...

coef(5)*X_search_local(i)*Y_search_local(j);

if Z_val > max_Z_val

max_Z_val = Z_val;

best_X_lasso_block(fold) = X_search_local(i);

best_Y_lasso_block(fold) = Y_search_local(j);

end

end

end

% ======= Method 3: Random Forest =======

rf_model = TreeBagger(50, [X_train, Y_train], Z_train, 'Method', 'regression');

Z_pred_rf = predict(rf_model, [X_test, Y_test]);

SS_res = sum((Z_test - Z_pred_rf).^2);

rf_R2_block(fold) = 1 - SS_res/SS_tot;

rf_RMSE_block(fold) = sqrt(mean((Z_test - Z_pred_rf).^2));

X_search_local = linspace(min(X_train), max(X_train), 100);

Y_search_local = linspace(min(Y_train), max(Y_train), 100);

max_Z_val = -inf;

for i = 1:length(X_search_local)

for j = 1:length(Y_search_local)

Z_val = predict(rf_model, [X_search_local(i), Y_search_local(j)]);

if Z_val > max_Z_val && ~isnan(Z_val)

max_Z_val = Z_val;

best_X_rf_block(fold) = X_search_local(i);

best_Y_rf_block(fold) = Y_search_local(j);

end

end

end

% ======= Method 4: Neural Network =======

X_min_train = min(X_train); X_max_train = max(X_train);

Y_min_train = min(Y_train); Y_max_train = max(Y_train);

Z_min_train = min(Z_train); Z_max_train = max(Z_train);

X_norm_train = (X_train - X_min_train) / (X_max_train - X_min_train);

Y_norm_train = (Y_train - Y_min_train) / (Y_max_train - Y_min_train);

Z_norm_train = (Z_train - Z_min_train) / (Z_max_train - Z_min_train);

rng(fold * 100 + 42);

net = feedforwardnet(5);

net.trainFcn = 'trainlm';

net.trainParam.epochs = 200;

net.trainParam.showWindow = false;

inputs_train = [X_norm_train'; Y_norm_train'];

targets_train = Z_norm_train';

[net_trained, ~] = train(net, inputs_train, targets_train);

X_norm_test = (X_test - X_min_train) / (X_max_train - X_min_train);

Y_norm_test = (Y_test - Y_min_train) / (Y_max_train - Y_min_train);

inputs_test = [X_norm_test'; Y_norm_test'];

Z_norm_pred = net_trained(inputs_test);

Z_pred_nn = Z_norm_pred' * (Z_max_train - Z_min_train) + Z_min_train;

SS_res = sum((Z_test - Z_pred_nn).^2);

nn_R2_block(fold) = 1 - SS_res/SS_tot;

nn_RMSE_block(fold) = sqrt(mean((Z_test - Z_pred_nn).^2));

X_search_local = linspace(min(X_train), max(X_train), 100);

Y_search_local = linspace(min(Y_train), max(Y_train), 100);

max_Z_val = -inf;

for i = 1:length(X_search_local)

for j = 1:length(Y_search_local)

X_norm_val = (X_search_local(i) - X_min_train) / (X_max_train - X_min_train);

Y_norm_val = (Y_search_local(j) - Y_min_train) / (Y_max_train - Y_min_train);

Z_pred_val = net_trained([X_norm_val; Y_norm_val]) * ...

(Z_max_train - Z_min_train) + Z_min_train;

if Z_pred_val > max_Z_val

max_Z_val = Z_pred_val;

best_X_nn_block(fold) = X_search_local(i);

best_Y_nn_block(fold) = Y_search_local(j);

end

end

end

fprintf('QR: R²=%.4f, RMSE=%.4f, Optimal X=%.4f\n', ...

quadratic_R2_block(fold), quadratic_RMSE_block(fold), best_X_quadratic_block(fold));

fprintf('Lasso: R²=%.4f, RMSE=%.4f, Optimal X=%.4f\n', ...

lasso_R2_block(fold), lasso_RMSE_block(fold), best_X_lasso_block(fold));

fprintf('RF: R²=%.4f, RMSE=%.4f, Optimal X=%.4f\n', ...

rf_R2_block(fold), rf_RMSE_block(fold), best_X_rf_block(fold));

fprintf('NN: R²=%.4f, RMSE=%.4f, Optimal X=%.4f\n', ...

nn_R2_block(fold), nn_RMSE_block(fold), best_X_nn_block(fold));

end

% ==================== 3.6 Spatial Block Cross-Validation ====================

fprintf('\n\n=== Spatial Block Cross-Validation (Spatial Block CV) ===\n');

fprintf('Description: Data is divided into 5 consecutive spatial blocks by the value range of solid density X\n');

fprintf('Purpose: Control the impact of spatial proximity on performance estimation\n');

% Sort by X value and partition into blocks

[X_sorted, sort_idx_X] = sort(X);

block_size = floor(n / K);

indices_spatial = zeros(n, 1);

for fold = 1:K

start_idx = (fold-1) * block_size + 1;

end_idx = min(fold * block_size, n);

for i = start_idx:end_idx

indices_spatial(sort_idx_X(i)) = fold;

end

end

if end_idx < n

for i = end_idx+1:n

indices_spatial(sort_idx_X(i)) = K;

end

end

fprintf('Solid density X ranges per fold (partitioned by value range):\n');

for fold = 1:K

idx_range = find(indices_spatial == fold);

x_min = min(X(idx_range));

x_max = max(X(idx_range));

fprintf(' Fold %d: X ∈ [%.4f, %.4f] (%d samples)\n', fold, x_min, x_max, length(idx_range));

end

% Storage for spatial block CV results

quadratic_R2_spatial = zeros(K, 1);

quadratic_RMSE_spatial = zeros(K, 1);

lasso_R2_spatial = zeros(K, 1);

lasso_RMSE_spatial = zeros(K, 1);

rf_R2_spatial = zeros(K, 1);

rf_RMSE_spatial = zeros(K, 1);

nn_R2_spatial = zeros(K, 1);

nn_RMSE_spatial = zeros(K, 1);

best_X_quadratic_spatial = zeros(K, 1);

best_X_lasso_spatial = zeros(K, 1);

best_X_rf_spatial = zeros(K, 1);

best_X_nn_spatial = zeros(K, 1);

best_Y_quadratic_spatial = zeros(K, 1);

best_Y_lasso_spatial = zeros(K, 1);

best_Y_rf_spatial = zeros(K, 1);

best_Y_nn_spatial = zeros(K, 1);

for fold = 1:K

fprintf('\n--- Fold %d Spatial Block Cross-Validation ---\n', fold);

test_idx = (indices_spatial == fold);

train_idx = ~test_idx;

X_train = X(train_idx);

Y_train = Y(train_idx);

Z_train = Z(train_idx);

X_test = X(test_idx);

Y_test = Y(test_idx);

Z_test = Z(test_idx);

SS_tot = sum((Z_test - mean(Z_test)).^2);

% ======= Method 1: Quadratic Regression =======

A_train = [ones(size(X_train)), X_train, Y_train, ...

X_train.^2, Y_train.^2, X_train.*Y_train];

coefficients = A_train \ Z_train;

A_test = [ones(size(X_test)), X_test, Y_test, ...

X_test.^2, Y_test.^2, X_test.*Y_test];

Z_pred_quad = A_test * coefficients;

SS_res = sum((Z_test - Z_pred_quad).^2);

quadratic_R2_spatial(fold) = 1 - SS_res/SS_tot;

quadratic_RMSE_spatial(fold) = sqrt(mean((Z_test - Z_pred_quad).^2));

B0c = coefficients(1); B1c = coefficients(2); B2c = coefficients(3);

B3c = coefficients(4); B4c = coefficients(5); B5c = coefficients(6);

M = [2*B3c, B5c; B5c, 2*B4c];

RHS = [-B1c; -B2c];

if abs(det(M)) > 1e-10

XY_opt = M \ RHS;

best_X_quadratic_spatial(fold) = XY_opt(1);

best_Y_quadratic_spatial(fold) = XY_opt(2);

else

X_search_local = linspace(min(X_train), max(X_train), 100);

Y_search_local = linspace(min(Y_train), max(Y_train), 100);

max_Z_val = -inf;

for i = 1:length(X_search_local)

for j = 1:length(Y_search_local)

Z_val = B0c + B1c*X_search_local(i) + B2c*Y_search_local(j) + ...

B3c*X_search_local(i)^2 + B4c*Y_search_local(j)^2 + ...

B5c*X_search_local(i)*Y_search_local(j);

if Z_val > max_Z_val

max_Z_val = Z_val;

best_X_quadratic_spatial(fold) = X_search_local(i);

best_Y_quadratic_spatial(fold) = Y_search_local(j);

end

end

end

end

% ======= Method 2: Lasso Regression =======

A_train_lasso = [X_train, Y_train, X_train.^2, Y_train.^2, X_train.*Y_train];

A_test_lasso = [X_test, Y_test, X_test.^2, Y_test.^2, X_test.*Y_test];

[B, info] = lasso(A_train_lasso, Z_train, 'CV', 5);

idx = info.IndexMinMSE;

intercept = info.Intercept(idx);

coef = B(:, idx);

Z_pred_lasso = intercept + A_test_lasso * coef;

SS_res = sum((Z_test - Z_pred_lasso).^2);

lasso_R2_spatial(fold) = 1 - SS_res/SS_tot;

lasso_RMSE_spatial(fold) = sqrt(mean((Z_test - Z_pred_lasso).^2));

X_search_local = linspace(min(X_train), max(X_train), 100);

Y_search_local = linspace(min(Y_train), max(Y_train), 100);

max_Z_val = -inf;

for i = 1:length(X_search_local)

for j = 1:length(Y_search_local)

Z_val = intercept + coef(1)*X_search_local(i) + coef(2)*Y_search_local(j) + ...

coef(3)*X_search_local(i)^2 + coef(4)*Y_search_local(j)^2 + ...

coef(5)*X_search_local(i)*Y_search_local(j);

if Z_val > max_Z_val

max_Z_val = Z_val;

best_X_lasso_spatial(fold) = X_search_local(i);

best_Y_lasso_spatial(fold) = Y_search_local(j);

end

end

end

% ======= Method 3: Random Forest =======

rf_model = TreeBagger(50, [X_train, Y_train], Z_train, 'Method', 'regression');

Z_pred_rf = predict(rf_model, [X_test, Y_test]);

SS_res = sum((Z_test - Z_pred_rf).^2);

rf_R2_spatial(fold) = 1 - SS_res/SS_tot;

rf_RMSE_spatial(fold) = sqrt(mean((Z_test - Z_pred_rf).^2));

X_search_local = linspace(min(X_train), max(X_train), 100);

Y_search_local = linspace(min(Y_train), max(Y_train), 100);

max_Z_val = -inf;

for i = 1:length(X_search_local)

for j = 1:length(Y_search_local)

Z_val = predict(rf_model, [X_search_local(i), Y_search_local(j)]);

if Z_val > max_Z_val && ~isnan(Z_val)

max_Z_val = Z_val;

best_X_rf_spatial(fold) = X_search_local(i);

best_Y_rf_spatial(fold) = Y_search_local(j);

end

end

end

% ======= Method 4: Neural Network =======

X_min_train = min(X_train); X_max_train = max(X_train);

Y_min_train = min(Y_train); Y_max_train = max(Y_train);

Z_min_train = min(Z_train); Z_max_train = max(Z_train);

X_norm_train = (X_train - X_min_train) / (X_max_train - X_min_train);

Y_norm_train = (Y_train - Y_min_train) / (Y_max_train - Y_min_train);

Z_norm_train = (Z_train - Z_min_train) / (Z_max_train - Z_min_train);

rng(fold * 100 + 84);

net = feedforwardnet(5);

net.trainFcn = 'trainlm';

net.trainParam.epochs = 200;

net.trainParam.showWindow = false;

inputs_train = [X_norm_train'; Y_norm_train'];

targets_train = Z_norm_train';

[net_trained, ~] = train(net, inputs_train, targets_train);

X_norm_test = (X_test - X_min_train) / (X_max_train - X_min_train);

Y_norm_test = (Y_test - Y_min_train) / (Y_max_train - Y_min_train);

inputs_test = [X_norm_test'; Y_norm_test'];

Z_norm_pred = net_trained(inputs_test);

Z_pred_nn = Z_norm_pred' * (Z_max_train - Z_min_train) + Z_min_train;

SS_res = sum((Z_test - Z_pred_nn).^2);

nn_R2_spatial(fold) = 1 - SS_res/SS_tot;

nn_RMSE_spatial(fold) = sqrt(mean((Z_test - Z_pred_nn).^2));

X_search_local = linspace(min(X_train), max(X_train), 100);

Y_search_local = linspace(min(Y_train), max(Y_train), 100);

max_Z_val = -inf;

for i = 1:length(X_search_local)

for j = 1:length(Y_search_local)

X_norm_val = (X_search_local(i) - X_min_train) / (X_max_train - X_min_train);

Y_norm_val = (Y_search_local(j) - Y_min_train) / (Y_max_train - Y_min_train);

Z_pred_val = net_trained([X_norm_val; Y_norm_val]) * ...

(Z_max_train - Z_min_train) + Z_min_train;

if Z_pred_val > max_Z_val

max_Z_val = Z_pred_val;

best_X_nn_spatial(fold) = X_search_local(i);

best_Y_nn_spatial(fold) = Y_search_local(j);

end

end

end

fprintf('QR: R²=%.4f, RMSE=%.4f, Optimal X=%.4f\n', ...

quadratic_R2_spatial(fold), quadratic_RMSE_spatial(fold), best_X_quadratic_spatial(fold));

fprintf('Lasso: R²=%.4f, RMSE=%.4f, Optimal X=%.4f\n', ...

lasso_R2_spatial(fold), lasso_RMSE_spatial(fold), best_X_lasso_spatial(fold));

fprintf('RF: R²=%.4f, RMSE=%.4f, Optimal X=%.4f\n', ...

rf_R2_spatial(fold), rf_RMSE_spatial(fold), best_X_rf_spatial(fold));

fprintf('NN: R²=%.4f, RMSE=%.4f, Optimal X=%.4f\n', ...

nn_R2_spatial(fold), nn_RMSE_spatial(fold), best_X_nn_spatial(fold));

end

% ==================== 4. Statistical Analysis of Results ====================

fprintf('\n\n=== Statistical Analysis of Four Methods (Random CV) ===\n');

fprintf('\n1. Fitting Performance Comparison (5-fold CV):\n');

fprintf('--------------------------------------------------------------------\n');

fprintf('Method	Mean R²	R² Std	Mean RMSE	RMSE Std\n');

fprintf('--------------------------------------------------------------------\n');

fprintf('Quadratic	%.4f	%.4f	%.4f	%.4f\n', ...

mean(quadratic_R2), std(quadratic_R2), mean(quadratic_RMSE), std(quadratic_RMSE));

fprintf('Lasso	%.4f	%.4f	%.4f	%.4f\n', ...

mean(lasso_R2), std(lasso_R2), mean(lasso_RMSE), std(lasso_RMSE));

fprintf('Random Forest	%.4f	%.4f	%.4f	%.4f\n', ...

mean(rf_R2), std(rf_R2), mean(rf_RMSE), std(rf_RMSE));

fprintf('Neural Network	%.4f	%.4f	%.4f	%.4f\n', ...

mean(nn_R2), std(nn_R2), mean(nn_RMSE), std(nn_RMSE));

fprintf('--------------------------------------------------------------------\n');

fprintf('\n2. Optimal Solid Density Stability Comparison (Random CV):\n');

fprintf('Quadratic optimal X: %.4f ± %.4f (CV=%.2f%%)\n', mean(best_X_quadratic), std(best_X_quadratic), std(best_X_quadratic)/mean(best_X_quadratic)*100);

fprintf('Lasso optimal X: %.4f ± %.4f (CV=%.2f%%)\n', mean(best_X_lasso), std(best_X_lasso), std(best_X_lasso)/mean(best_X_lasso)*100);

fprintf('Random Forest optimal X: %.4f ± %.4f (CV=%.2f%%)\n', mean(best_X_rf), std(best_X_rf), std(best_X_rf)/mean(best_X_rf)*100);

fprintf('Neural Network optimal X: %.4f ± %.4f (CV=%.2f%%)\n', mean(best_X_nn), std(best_X_nn), std(best_X_nn)/mean(best_X_nn)*100);

% ==================== 4.5 Three CV Strategy Results Comparison ====================

fprintf('\n\n=== Comparison of Three Cross-Validation Strategies ===\n');

fprintf('Description:\n');

fprintf(' - Random CV: Randomly shuffled folds (baseline)\n');

fprintf(' - Temporal Block CV: Consecutive order folds (controls time dependence)\n');

fprintf(' - Spatial Block CV: X-value range folds (controls spatial dependence)\n');

fprintf('====================================================================\n');

fprintf('Model	| Metric | Random CV	| Temporal Block	| Spatial Block	\n');

fprintf('====================================================================\n');

print_compare3 = @(name, metric, val_random, val_block, val_spatial) ...

fprintf('%s	| %s	| %.4f ± %.4f | %.4f ± %.4f | %.4f ± %.4f\n', ...

name, metric, mean(val_random), std(val_random), mean(val_block), std(val_block), mean(val_spatial), std(val_spatial));

print_compare3('QR', 'R²', quadratic_R2, quadratic_R2_block, quadratic_R2_spatial);

print_compare3('Lasso', 'R²', lasso_R2, lasso_R2_block, lasso_R2_spatial);

print_compare3('RF', 'R²', rf_R2, rf_R2_block, rf_R2_spatial);

print_compare3('NN', 'R²', nn_R2, nn_R2_block, nn_R2_spatial);

print_compare3('QR', 'RMSE', quadratic_RMSE, quadratic_RMSE_block, quadratic_RMSE_spatial);

print_compare3('Lasso', 'RMSE', lasso_RMSE, lasso_RMSE_block, lasso_RMSE_spatial);

print_compare3('RF', 'RMSE', rf_RMSE, rf_RMSE_block, rf_RMSE_spatial);

print_compare3('NN', 'RMSE', nn_RMSE, nn_RMSE_block, nn_RMSE_spatial);

fprintf('====================================================================\n');

fprintf('\nModel ranking by strategy (by R²):\n');

fprintf(' Random CV: RF=%.4f, NN=%.4f, QR=%.4f, Lasso=%.4f\n', ...

mean(rf_R2), mean(nn_R2), mean(quadratic_R2), mean(lasso_R2));

fprintf(' Temporal CV: RF=%.4f, NN=%.4f, QR=%.4f, Lasso=%.4f\n', ...

mean(rf_R2_block), mean(nn_R2_block), mean(quadratic_R2_block), mean(lasso_R2_block));

fprintf(' Spatial CV: RF=%.4f, NN=%.4f, QR=%.4f, Lasso=%.4f\n', ...

mean(rf_R2_spatial), mean(nn_R2_spatial), mean(quadratic_R2_spatial), mean(lasso_R2_spatial));

% Generate bar chart comparing three CV strategies

figure('Position', [100, 100, 900, 600]);

bar_data = [mean(quadratic_R2), mean(lasso_R2), mean(rf_R2), mean(nn_R2);

mean(quadratic_R2_block), mean(lasso_R2_block), mean(rf_R2_block), mean(nn_R2_block);

mean(quadratic_R2_spatial), mean(lasso_R2_spatial), mean(rf_R2_spatial), mean(nn_R2_spatial)];

bar(bar_data);

set(gca, 'XTickLabel', {'QR', 'Lasso', 'RF', 'NN'});

legend('Random CV', 'Temporal Block CV', 'Spatial Block CV', 'Location', 'best');

ylabel('Mean R²');

title('Cyan Ink: R² Comparison Across Three CV Strategies');

grid on;

saveas(gcf, 'Cyan_Ink_CV_Comparison_Three.png');

fprintf('\nThree-strategy comparison figure saved: Cyan_Ink_CV_Comparison_Three.png\n');

% ==================== 5. Statistical Significance Test (Paired t-test) ====================

fprintf('\n=== Statistical Significance Test (Paired t-test) - Based on Random CV ===\n');

diff_R2_NN_QR = nn_R2 - quadratic_R2;

diff_R2_RF_QR = rf_R2 - quadratic_R2;

diff_R2_RF_NN = rf_R2 - nn_R2;

diff_R2_Lasso_QR = lasso_R2 - quadratic_R2;

[~, p_NN_QR] = ttest(diff_R2_NN_QR);

fprintf('\nNN vs QR:\n');

fprintf(' Mean R² difference: +%.4f\n', mean(diff_R2_NN_QR));

fprintf(' p = %.4f', p_NN_QR);

if p_NN_QR < 0.05

fprintf(' (Significant difference)\n');

else

fprintf(' (No significant difference)\n');

end

[~, p_RF_QR] = ttest(diff_R2_RF_QR);

fprintf('\nRF vs QR:\n');

fprintf(' Mean R² difference: +%.4f\n', mean(diff_R2_RF_QR));

fprintf(' p = %.4f', p_RF_QR);

if p_RF_QR < 0.05

fprintf(' (Significant difference)\n');

else

fprintf(' (No significant difference)\n');

end

[~, p_RF_NN] = ttest(diff_R2_RF_NN);

fprintf('\nRF vs NN:\n');

fprintf(' Mean R² difference: +%.4f\n', mean(diff_R2_RF_NN));

fprintf(' p = %.4f', p_RF_NN);

if p_RF_NN < 0.05

fprintf(' (Significant difference)\n');

else

fprintf(' (No significant difference)\n');

end

fprintf('\nLasso vs QR:\n');

fprintf(' Mean R² difference: +%.4f (No significant difference, p > 0.05)\n', mean(diff_R2_Lasso_QR));

% ==================== 6. Response Surface Analysis ====================

fprintf('\n=== Generating Response Surfaces ===\n');

xg = linspace(min(X), max(X), 100);

yg = linspace(min(Y), max(Y), 100);

[Xg, Yg] = meshgrid(xg, yg);

% Calculate actual data range

in_range = (Xg >= min(X)) & (Xg <= max(X)) & ...

(Yg >= min(Y)) & (Yg <= max(Y));

% 6.1 QR response surface

Zg_qr = B0 + B1*Xg + B2*Yg + B3*Xg.^2 + B4*Yg.^2 + B5*Xg.*Yg;

Zg_qr(Zg_qr < 0) = NaN;

Zg_qr(~in_range) = NaN;

% 6.2 Lasso response surface

Zg_lasso = intercept_lasso + coef_lasso(1)*Xg + coef_lasso(2)*Yg + ...

coef_lasso(3)*Xg.^2 + coef_lasso(4)*Yg.^2 + coef_lasso(5)*Xg.*Yg;

Zg_lasso(Zg_lasso < 0) = NaN;

Zg_lasso(~in_range) = NaN;

% 6.3 RF response surface

Zg_rf = predict(rf_full, [Xg(:), Yg(:)]);

Zg_rf = reshape(Zg_rf, size(Xg));

Zg_rf(Zg_rf < 0) = NaN;

Zg_rf(~in_range) = NaN;

% 6.4 NN response surface

Xg_norm = (Xg - X_min) / (X_max - X_min);

Yg_norm = (Yg - Y_min) / (Y_max - Y_min);

Zg_nn = net_full([Xg_norm(:)'; Yg_norm(:)'])';

Zg_nn = reshape(Zg_nn, size(Xg)) * (Z_max - Z_min) + Z_min;

Zg_nn(Zg_nn < 0) = NaN;

Zg_nn(~in_range) = NaN;

% Generate 2×2 comparison figure

figure('Position', [100, 100, 1200, 1000], 'Name', 'Cyan Ink: Four Model Response Surface Comparison');

subplot(2,2,1);

surf(Xg, Yg, Zg_qr, 'EdgeColor', 'none', 'FaceAlpha', 0.9);

hold on;

scatter3(X, Y, Z, 25, 'k', 'filled', 'MarkerFaceAlpha', 0.4);

title(sprintf('(a) Quadratic Regression QR (R²=%.4f)', R2_quad_full));

xlabel('Solid Density X'); ylabel('Dot Gain Y'); zlabel('Contrast Z');

colormap(jet); colorbar; view(45, 30); grid on;

zlim([min(Z), max(Z)]);

subplot(2,2,2);

surf(Xg, Yg, Zg_lasso, 'EdgeColor', 'none', 'FaceAlpha', 0.9);

hold on;

scatter3(X, Y, Z, 25, 'k', 'filled', 'MarkerFaceAlpha', 0.4);

title(sprintf('(b) Lasso Regression (R²=%.4f)', R2_lasso_full));

xlabel('Solid Density X'); ylabel('Dot Gain Y'); zlabel('Contrast Z');

colormap(jet); colorbar; view(45, 30); grid on;

zlim([min(Z), max(Z)]);

subplot(2,2,3);

surf(Xg, Yg, Zg_rf, 'EdgeColor', 'none', 'FaceAlpha', 0.9);

hold on;

scatter3(X, Y, Z, 25, 'k', 'filled', 'MarkerFaceAlpha', 0.4);

title(sprintf('(c) Random Forest RF (R²=%.4f)', R2_rf_full));

xlabel('Solid Density X'); ylabel('Dot Gain Y'); zlabel('Contrast Z');

colormap(jet); colorbar; view(45, 30); grid on;

zlim([min(Z), max(Z)]);

subplot(2,2,4);

surf(Xg, Yg, Zg_nn, 'EdgeColor', 'none', 'FaceAlpha', 0.9);

hold on;

scatter3(X, Y, Z, 25, 'k', 'filled', 'MarkerFaceAlpha', 0.4);

title(sprintf('(d) Neural Network MLP (R²=%.4f)', R2_nn_full));

xlabel('Solid Density X'); ylabel('Dot Gain Y'); zlabel('Contrast Z');

colormap(jet); colorbar; view(45, 30); grid on;

zlim([min(Z), max(Z)]);

sgtitle('Cyan Ink: Four Model Response Surface Comparison');

saveas(gcf, 'Cyan_Ink_ResponseSurfaces.png');

fprintf(' Response surface figure saved: Cyan_Ink_ResponseSurfaces.png\n');

% ==================== 7. Cross-Validation Analysis ====================

fprintf('\n=== Optimal Point Cross-Validation Analysis ===\n');

Z_nn_at_qr = interp2(Xg, Yg, Zg_nn, X_opt_quad, Y_opt_quad);

Z_rf_at_qr = interp2(Xg, Yg, Zg_rf, X_opt_quad, Y_opt_quad);

Z_lasso_at_qr = interp2(Xg, Yg, Zg_lasso, X_opt_quad, Y_opt_quad);

Z_qr_at_nn = interp2(Xg, Yg, Zg_qr, best_X_nn_full, best_Y_nn_full);

Z_rf_at_nn = interp2(Xg, Yg, Zg_rf, best_X_nn_full, best_Y_nn_full);

Z_lasso_at_nn = interp2(Xg, Yg, Zg_lasso, best_X_nn_full, best_Y_nn_full);

Z_qr_at_rf = interp2(Xg, Yg, Zg_qr, best_X_rf_full, best_Y_rf_full);

Z_nn_at_rf = interp2(Xg, Yg, Zg_nn, best_X_rf_full, best_Y_rf_full);

Z_lasso_at_rf = interp2(Xg, Yg, Zg_lasso, best_X_rf_full, best_Y_rf_full);

fprintf('\nQR optimum (X=%.4f, Y=%.4f, Z=%.4f):\n', X_opt_quad, Y_opt_quad, Z_opt_quad);

fprintf(' Prediction on NN: Z = %.4f (Diff: %+.4f)\n', Z_nn_at_qr, Z_nn_at_qr - Z_opt_quad);

fprintf(' Prediction on RF: Z = %.4f (Diff: %+.4f)\n', Z_rf_at_qr, Z_rf_at_qr - Z_opt_quad);

fprintf(' Prediction on Lasso: Z = %.4f (Diff: %+.4f)\n', Z_lasso_at_qr, Z_lasso_at_qr - Z_opt_quad);

fprintf('\nNN optimum (X=%.4f, Y=%.4f, Z=%.4f):\n', best_X_nn_full, best_Y_nn_full, max_Z_nn);

fprintf(' Prediction on QR: Z = %.4f (Diff: %+.4f)\n', Z_qr_at_nn, Z_qr_at_nn - max_Z_nn);

fprintf(' Prediction on RF: Z = %.4f (Diff: %+.4f)\n', Z_rf_at_nn, Z_rf_at_nn - max_Z_nn);

fprintf(' Prediction on Lasso: Z = %.4f (Diff: %+.4f)\n', Z_lasso_at_nn, Z_lasso_at_nn - max_Z_nn);

fprintf('\nRF optimum (X=%.4f, Y=%.4f, Z=%.4f):\n', best_X_rf_full, best_Y_rf_full, max_Z_rf);

fprintf(' Prediction on QR: Z = %.4f (Diff: %+.4f)\n', Z_qr_at_rf, Z_qr_at_rf - max_Z_rf);

fprintf(' Prediction on NN: Z = %.4f (Diff: %+.4f)\n', Z_nn_at_rf, Z_nn_at_rf - max_Z_rf);

fprintf(' Prediction on Lasso: Z = %.4f (Diff: %+.4f)\n', Z_lasso_at_rf, Z_lasso_at_rf - max_Z_rf);

% ==================== 8. Residual Analysis ====================

fprintf('\n=== Residual Analysis ===\n');

residuals_qr = Z - Z_pred_quad_full;

residuals_lasso = Z - Z_pred_lasso_full;

residuals_rf = Z - Z_pred_rf_full;

residuals_nn = Z - Z_pred_nn_full;

figure('Position', [100, 100, 800, 800], 'Name', 'Cyan Ink Residual Analysis');

subplot(2,2,1);

scatter(Z_pred_quad_full, residuals_qr, 'b.');

hold on;

plot([min(Z_pred_quad_full), max(Z_pred_quad_full)], [0, 0], 'r--', 'LineWidth', 1);

xlabel('Predicted Value'); ylabel('Residual'); title('(a) QR Residual Plot');

grid on;

subplot(2,2,2);

scatter(Z_pred_lasso_full, residuals_lasso, 'g.');

hold on;

plot([min(Z_pred_lasso_full), max(Z_pred_lasso_full)], [0, 0], 'r--', 'LineWidth', 1);

xlabel('Predicted Value'); ylabel('Residual'); title('(b) Lasso Residual Plot');

grid on;

subplot(2,2,3);

scatter(Z_pred_rf_full, residuals_rf, 'r.');

hold on;

plot([min(Z_pred_rf_full), max(Z_pred_rf_full)], [0, 0], 'r--', 'LineWidth', 1);

xlabel('Predicted Value'); ylabel('Residual'); title('(c) RF Residual Plot');

grid on;

subplot(2,2,4);

scatter(Z_pred_nn_full, residuals_nn, 'm.');

hold on;

plot([min(Z_pred_nn_full), max(Z_pred_nn_full)], [0, 0], 'r--', 'LineWidth', 1);

xlabel('Predicted Value'); ylabel('Residual'); title('(d) NN Residual Plot');

grid on;

sgtitle('Cyan Ink: Four Model Residual Distribution Comparison');

saveas(gcf, 'Cyan_Ink_Residuals.png');

% ==================== 9. Generate Comparison Charts ====================

fprintf('\n=== Generating Comparison Charts ===\n');

% Figure 1: R² boxplot

figure('Position', [100, 100, 600, 500], 'Name', 'Cyan Ink R² Boxplot');

boxplot([quadratic_R2, lasso_R2, rf_R2, nn_R2], 'Labels', {'QR', 'Lasso', 'RF', 'NN'});

ylabel('Coefficient of Determination R²');

title('Cyan Ink: R² Distribution Comparison (Random 5-fold CV)');

grid on;

saveas(gcf, 'Cyan_Ink_R2_Boxplot.png');

% Figure 2: RMSE boxplot

figure('Position', [150, 150, 600, 500], 'Name', 'Cyan Ink RMSE Boxplot');

boxplot([quadratic_RMSE, lasso_RMSE, rf_RMSE, nn_RMSE], 'Labels', {'QR', 'Lasso', 'RF', 'NN'});

ylabel('Root Mean Square Error RMSE');

title('Cyan Ink: RMSE Distribution Comparison (Random 5-fold CV)');

grid on;

saveas(gcf, 'Cyan_Ink_RMSE_Boxplot.png');

% Figure 3: Optimal X stability line chart (Random CV)

figure('Position', [200, 200, 700, 500], 'Name', 'Cyan Ink Optimal X Stability');

hold on;

plot(1:K, best_X_quadratic, 'bo-', 'LineWidth', 2, 'MarkerSize', 8, 'DisplayName', 'QR');

plot(1:K, best_X_lasso, 'go-', 'LineWidth', 2, 'MarkerSize', 8, 'DisplayName', 'Lasso');

plot(1:K, best_X_rf, 'ro-', 'LineWidth', 2, 'MarkerSize', 8, 'DisplayName', 'RF');

plot(1:K, best_X_nn, 'mo-', 'LineWidth', 2, 'MarkerSize', 8, 'DisplayName', 'NN');

xlabel('Cross-Validation Fold'); ylabel('Optimal Solid Density X');

title('Cyan Ink: Optimal Solid Density Variation Across Folds (Random CV)');

legend('Location', 'best'); grid on;

xlim([0.5, 5.5]);

saveas(gcf, 'Cyan_Ink_Xopt_Stability.png');

% Figure 4: Mean performance bar chart

figure('Position', [250, 250, 800, 500], 'Name', 'Cyan Ink Mean Performance Comparison');

subplot(1,2,1);

mean_R2 = [mean(quadratic_R2), mean(lasso_R2), mean(rf_R2), mean(nn_R2)];

std_R2 = [std(quadratic_R2), std(lasso_R2), std(rf_R2), std(nn_R2)];

bar(1:4, mean_R2, 0.6, 'FaceColor', [0.5 0.5 0.8]);

hold on;

errorbar(1:4, mean_R2, std_R2, 'k.', 'LineWidth', 1.5, 'MarkerSize', 15);

set(gca, 'XTickLabel', {'QR', 'Lasso', 'RF', 'NN'});

ylabel('Mean R²'); title('Cyan Ink: Mean R² Comparison');

ylim([0.5, 1.0]); grid on;

subplot(1,2,2);

mean_RMSE = [mean(quadratic_RMSE), mean(lasso_RMSE), mean(rf_RMSE), mean(nn_RMSE)];

std_RMSE = [std(quadratic_RMSE), std(lasso_RMSE), std(rf_RMSE), std(nn_RMSE)];

bar(1:4, mean_RMSE, 0.6, 'FaceColor', [0.8 0.5 0.5]);

hold on;

errorbar(1:4, mean_RMSE, std_RMSE, 'k.', 'LineWidth', 1.5, 'MarkerSize', 15);

set(gca, 'XTickLabel', {'QR', 'Lasso', 'RF', 'NN'});

ylabel('Mean RMSE'); title('Cyan Ink: Mean RMSE Comparison');

grid on;

sgtitle('Cyan Ink: Four Model Mean Performance Comparison (Random 5-fold CV)');

saveas(gcf, 'Cyan_Ink_Performance_Bar.png');

fprintf('\n=== Cyan Ink Analysis Complete! ===\n');

fprintf('Generated image files:\n');

fprintf(' Cyan_Ink_Autocorrelation.png\n');

fprintf(' Cyan_Ink_CV_Comparison_Three.png <- New: Three CV strategy comparison\n');

fprintf(' Cyan_Ink_ResponseSurfaces.png\n');

fprintf(' Cyan_Ink_Residuals.png\n');

fprintf(' Cyan_Ink_R2_Boxplot.png\n');

fprintf(' Cyan_Ink_RMSE_Boxplot.png\n');

fprintf(' Cyan_Ink_Xopt_Stability.png\n');

fprintf(' Cyan_Ink_Performance_Bar.png\n');

## ## 3. Magenta Ink

% ==================== 1. Data Preparation ====================

fprintf('=== Magenta Ink Data Preparation ===\n');

DATA = [

0.85	11.65	28.71

0.85	11.64	28.73

0.86	11.64	28.72

0.86	11.62	28.76

0.87	11.68	29.81

0.87	11.71	31.95

0.88	11.77	31.92

0.89	11.79	31.97

0.9	11.77	31.93

0.91	11.76	31.99

0.92	11.78	32.08

0.93	11.79	32.07

0.94	11.81	32.06

0.95	11.86	32.07

0.97	11.81	32.03

0.99	11.84	32.01

1	11.81	33.85

1.12	11.89	34.05

1.15	12.08	34.91

1.16	12.34	35.11

1.17	12.78	35.19

1.19	12.84	35.27

1.2	12.81	35.61

1.22	12.8	35.97

1.25	12.79	36.02

1.28	12.82	37.53

1.29	12.81	37.2

1.3	14.37	38.72

1.31	14.38	38.71

1.32	12.46	38.82

1.32	14.34	38.75

1.33	12.45	38.99

1.33	12.47	39.19

1.33	14.38	39.18

1.34	12.44	39.94

1.34	13.51	40.12

1.34	13.52	40.15

1.34	14.39	40.14

1.35	13.5	40.65

1.35	13.52	40.64

1.35	14.03	40.66

1.36	12.48	41.06

1.36	13.51	41.18

1.36	14.08	41.16

1.37	14.07	42.24

1.37	14.05	42.26

1.37	14.62	42.25

1.38	14.09	43.11

1.38	14.61	43.19

1.39	14.6	43.68

1.4	14.61	44.99

1.41	14.63	45.23

1.41	15.16	45.2

1.41	15.47	45.2

1.42	15.18	44.82

1.42	15.17	44.83

1.42	15.48	44.81

1.43	15.19	44.52

1.43	15.49	44.42

1.43	15.48	44.41

1.43	15.66	44.56

1.44	15.17	44.24

1.44	15.67	44.22

1.45	15.67	44.12

1.45	16.19	44.14

1.46	16.18	44.15

1.46	16.16	44.12

1.47	16.18	44.05

1.47	16.52	44.04

1.48	16.53	44.05

1.48	16.53	44.03

1.49	16.97	44.04

1.49	16.96	44.06

1.5	16.97	44.06

1.5	16.98	44.04

1.5	17.31	44.07

1.51	17.32	43.65

1.52	17.33	43.65

1.53	17.33	42.32

1.53	17.35	42.38

1.54	17.63	41.72

1.55	17.66	41.46

1.55	17.67	41.15

1.56	17.65	41.05

1.57	17.64	40.65

1.6	18.07	40.38

1.6	18.06	40.36

1.61	18.06	40.16

1.61	18.07	39.95

1.62	18.08	39.39

1.62	22.92	39.34

1.63	18.53	39.14

1.63	22.93	39.12

1.64	18.52	38.48

1.64	18.54	38.42

1.64	22.94	38.42

1.64	22.94	38.43

1.65	18.53	37.71

1.65	18.52	37.11

1.65	22.95	37.74

1.65	24.65	37.62

1.65	24.68	37.64

1.66	24.67	37.13

1.66	24.67	37.15

1.66	25.36	37.23

1.67	24.66	36.62

1.67	25.37	36.55

1.68	25.37	35.21

1.68	25.35	35.24

1.69	25.38	34.23

];

X = DATA(:, 1);

Y = DATA(:, 2);

Z = DATA(:, 3);

n = length(X);

fprintf('Sample size: %d\n', n);

fprintf('Solid density range: %.2f - %.2f\n', min(X), max(X));

fprintf('Dot gain range: %.2f - %.2f\n', min(Y), max(Y));

fprintf('Contrast range: %.2f - %.2f\n\n', min(Z), max(Z));

% ==================== 1.5 Autocorrelation Analysis ====================

fprintf('\n=== Autocorrelation Analysis ===\n');

% Manual autocorrelation calculation

max_lag = 20;

Z_mean = mean(Z);

Z_var = var(Z);

acf_manual = zeros(max_lag + 1, 1);

for lag = 0:max_lag

if lag == 0

acf_manual(lag+1) = 1;

else

sum_cov = 0;

for i = 1:(n-lag)

sum_cov = sum_cov + (Z(i) - Z_mean) * (Z(i+lag) - Z_mean);

end

acf_manual(lag+1) = sum_cov / ((n-lag) * Z_var);

end

end

lags_manual = (0:max_lag)';

conf = 1.96 / sqrt(n);

figure('Position', [100, 100, 800, 500]);

stem(lags_manual, acf_manual, 'filled', 'LineWidth', 1.5);

hold on;

plot([0, max_lag], [conf, conf], 'r--', 'LineWidth', 1);

plot([0, max_lag], [-conf, -conf], 'r--', 'LineWidth', 1);

xlabel('Lag'); ylabel('Autocorrelation Coefficient');

title('Autocorrelation Function of Magenta Ink Contrast Values');

grid on;

saveas(gcf, 'Magenta_Ink_Autocorrelation.png');

fprintf('Lag-1 autocorrelation coefficient: %.4f\n', acf_manual(2));

% ==================== 2. Full-Data Analysis (Baseline) ====================

fprintf('=== Full-Data Baseline Analysis ===\n');

% 2.1 Quadratic regression full-data fitting

A_full = [ones(size(X)), X, Y, X.^2, Y.^2, X.*Y];

coeff_full = A_full \ Z;

Z_pred_quad_full = A_full * coeff_full;

R2_quad_full = 1 - sum((Z - Z_pred_quad_full).^2) / sum((Z - mean(Z)).^2);

B0 = coeff_full(1); B1 = coeff_full(2); B2 = coeff_full(3);

B3 = coeff_full(4); B4 = coeff_full(5); B5 = coeff_full(6);

M = [2*B3, B5; B5, 2*B4];

RHS = [-B1; -B2];

if abs(det(M)) > 1e-10

XY_opt_quad = M \ RHS;

X_opt_quad = XY_opt_quad(1);

Y_opt_quad = XY_opt_quad(2);

else

X_search = linspace(min(X), max(X), 100);

Y_search = linspace(min(Y), max(Y), 100);

max_Z = -inf;

for i = 1:length(X_search)

for j = 1:length(Y_search)

Z_val = B0 + B1*X_search(i) + B2*Y_search(j) + ...

B3*X_search(i)^2 + B4*Y_search(j)^2 + ...

B5*X_search(i)*Y_search(j);

if Z_val > max_Z

max_Z = Z_val;

X_opt_quad = X_search(i);

Y_opt_quad = Y_search(j);

end

end

end

end

Z_opt_quad = B0 + B1*X_opt_quad + B2*Y_opt_quad + B3*X_opt_quad^2 + ...

B4*Y_opt_quad^2 + B5*X_opt_quad*Y_opt_quad;

fprintf('\nQuadratic Regression (full data):\n');

fprintf(' R² = %.4f\n', R2_quad_full);

fprintf(' Optimal solid density X = %.4f\n', X_opt_quad);

fprintf(' Optimal dot gain Y = %.4f\n', Y_opt_quad);

fprintf(' Predicted maximum contrast Z = %.4f\n', Z_opt_quad);

% 2.2 Lasso full-data fitting

fprintf('\nLasso Regression (full data):\n');

A_lasso_full = [X, Y, X.^2, Y.^2, X.*Y];

[B_lasso, info_lasso] = lasso(A_lasso_full, Z, 'CV', 5);

idx_lasso = info_lasso.IndexMinMSE;

intercept_lasso = info_lasso.Intercept(idx_lasso);

coef_lasso = B_lasso(:, idx_lasso);

Z_pred_lasso_full = intercept_lasso + A_lasso_full * coef_lasso;

R2_lasso_full = 1 - sum((Z - Z_pred_lasso_full).^2) / sum((Z - mean(Z)).^2);

% Grid search for Lasso optimal point

X_search = linspace(1.3, 1.7, 100);

Y_search = linspace(14, 26, 100);

max_Z_lasso = -inf;

best_X_lasso_full = NaN;

best_Y_lasso_full = NaN;

for i = 1:length(X_search)

for j = 1:length(Y_search)

Z_val = intercept_lasso + coef_lasso(1)*X_search(i) + coef_lasso(2)*Y_search(j) + ...

coef_lasso(3)*X_search(i)^2 + coef_lasso(4)*Y_search(j)^2 + ...

coef_lasso(5)*X_search(i)*Y_search(j);

if Z_val > max_Z_lasso

max_Z_lasso = Z_val;

best_X_lasso_full = X_search(i);

best_Y_lasso_full = Y_search(j);

end

end

end

fprintf(' R² = %.4f\n', R2_lasso_full);

fprintf(' Optimal solid density X = %.4f\n', best_X_lasso_full);

fprintf(' Optimal dot gain Y = %.4f\n', best_Y_lasso_full);

fprintf(' Predicted maximum contrast Z = %.4f\n', max_Z_lasso);

% 2.3 Random forest full-data fitting

fprintf('\nRandom Forest (full data):\n');

rf_full = TreeBagger(50, [X, Y], Z, 'Method', 'regression');

Z_pred_rf_full = predict(rf_full, [X, Y]);

R2_rf_full = 1 - sum((Z - Z_pred_rf_full).^2) / sum((Z - mean(Z)).^2);

% Grid search for RF optimal point

X_search = linspace(1.3, 1.7, 100);

Y_search = linspace(14, 26, 100);

max_Z_rf = -inf;

best_X_rf_full = NaN;

best_Y_rf_full = NaN;

for i = 1:length(X_search)

for j = 1:length(Y_search)

Z_val = predict(rf_full, [X_search(i), Y_search(j)]);

if Z_val > max_Z_rf && ~isnan(Z_val)

max_Z_rf = Z_val;

best_X_rf_full = X_search(i);

best_Y_rf_full = Y_search(j);

end

end

end

fprintf(' R² = %.4f\n', R2_rf_full);

fprintf(' Optimal solid density X = %.4f\n', best_X_rf_full);

fprintf(' Optimal dot gain Y = %.4f\n', best_Y_rf_full);

fprintf(' Predicted maximum contrast Z = %.4f\n', max_Z_rf);

% 2.4 Neural network full-data fitting

fprintf('\nNeural Network (full data):\n');

X_min = min(X); X_max = max(X);

Y_min = min(Y); Y_max = max(Y);

Z_min = min(Z); Z_max = max(Z);

X_norm = (X - X_min) / (X_max - X_min);

Y_norm = (Y - Y_min) / (Y_max - Y_min);

Z_norm = (Z - Z_min) / (Z_max - Z_min);

rng(42);

net_full = feedforwardnet(5);

net_full.trainFcn = 'trainlm';

net_full.trainParam.epochs = 300;

net_full.trainParam.showWindow = false;

inputs_full = [X_norm'; Y_norm'];

targets_full = Z_norm';

[net_full, ~] = train(net_full, inputs_full, targets_full);

Z_pred_norm = net_full(inputs_full);

Z_pred_nn_full = Z_pred_norm' * (Z_max - Z_min) + Z_min;

R2_nn_full = 1 - sum((Z - Z_pred_nn_full).^2) / sum((Z - mean(Z)).^2);

X_search = linspace(1.3, 1.7, 100);

Y_search = linspace(14, 26, 100);

max_Z_nn = -inf;

best_X_nn_full = NaN;

best_Y_nn_full = NaN;

for i = 1:length(X_search)

for j = 1:length(Y_search)

X_norm_val = (X_search(i) - X_min) / (X_max - X_min);

Y_norm_val = (Y_search(j) - Y_min) / (Y_max - Y_min);

Z_pred_val = net_full([X_norm_val; Y_norm_val]) * (Z_max - Z_min) + Z_min;

if Z_pred_val > max_Z_nn

max_Z_nn = Z_pred_val;

best_X_nn_full = X_search(i);

best_Y_nn_full = Y_search(j);

end

end

end

fprintf(' R² = %.4f\n', R2_nn_full);

fprintf(' Optimal solid density X = %.4f\n', best_X_nn_full);

fprintf(' Optimal dot gain Y = %.4f\n', best_Y_nn_full);

fprintf(' Predicted maximum contrast Z = %.4f\n', max_Z_nn);

% ==================== 3. Random 5-Fold Cross-Validation ====================

fprintf('\n\n=== Random 5-Fold Cross-Validation Method Comparison ===\n');

K = 5;

rng(42);

indices = crossvalind('Kfold', n, K);

% Storage for 4 methods' results (random CV)

quadratic_R2 = zeros(K, 1);

quadratic_RMSE = zeros(K, 1);

lasso_R2 = zeros(K, 1);

lasso_RMSE = zeros(K, 1);

rf_R2 = zeros(K, 1);

rf_RMSE = zeros(K, 1);

nn_R2 = zeros(K, 1);

nn_RMSE = zeros(K, 1);

best_X_quadratic = zeros(K, 1);

best_X_lasso = zeros(K, 1);

best_X_rf = zeros(K, 1);

best_X_nn = zeros(K, 1);

best_Y_quadratic = zeros(K, 1);

best_Y_lasso = zeros(K, 1);

best_Y_rf = zeros(K, 1);

best_Y_nn = zeros(K, 1);

for fold = 1:K

fprintf('\n--- Fold %d Cross-Validation ---\n', fold);

test_idx = (indices == fold);

train_idx = ~test_idx;

X_train = X(train_idx);

Y_train = Y(train_idx);

Z_train = Z(train_idx);

X_test = X(test_idx);

Y_test = Y(test_idx);

Z_test = Z(test_idx);

SS_tot = sum((Z_test - mean(Z_test)).^2);

% ======= Method 1: Quadratic Regression =======

A_train = [ones(size(X_train)), X_train, Y_train, ...

X_train.^2, Y_train.^2, X_train.*Y_train];

coefficients = A_train \ Z_train;

A_test = [ones(size(X_test)), X_test, Y_test, ...

X_test.^2, Y_test.^2, X_test.*Y_test];

Z_pred_quad = A_test * coefficients;

SS_res = sum((Z_test - Z_pred_quad).^2);

quadratic_R2(fold) = 1 - SS_res/SS_tot;

quadratic_RMSE(fold) = sqrt(mean((Z_test - Z_pred_quad).^2));

B0c = coefficients(1); B1c = coefficients(2); B2c = coefficients(3);

B3c = coefficients(4); B4c = coefficients(5); B5c = coefficients(6);

M = [2*B3c, B5c; B5c, 2*B4c];

RHS = [-B1c; -B2c];

if abs(det(M)) > 1e-10

XY_opt = M \ RHS;

best_X_quadratic(fold) = XY_opt(1);

best_Y_quadratic(fold) = XY_opt(2);

else

X_search_local = linspace(min(X_train), max(X_train), 100);

Y_search_local = linspace(min(Y_train), max(Y_train), 100);

max_Z_val = -inf;

for i = 1:length(X_search_local)

for j = 1:length(Y_search_local)

Z_val = B0c + B1c*X_search_local(i) + B2c*Y_search_local(j) + ...

B3c*X_search_local(i)^2 + B4c*Y_search_local(j)^2 + ...

B5c*X_search_local(i)*Y_search_local(j);

if Z_val > max_Z_val

max_Z_val = Z_val;

best_X_quadratic(fold) = X_search_local(i);

best_Y_quadratic(fold) = Y_search_local(j);

end

end

end

end

% ======= Method 2: Lasso Regression =======

A_train_lasso = [X_train, Y_train, X_train.^2, Y_train.^2, X_train.*Y_train];

A_test_lasso = [X_test, Y_test, X_test.^2, Y_test.^2, X_test.*Y_test];

[B, info] = lasso(A_train_lasso, Z_train, 'CV', 5);

idx = info.IndexMinMSE;

intercept = info.Intercept(idx);

coef = B(:, idx);

Z_pred_lasso = intercept + A_test_lasso * coef;

SS_res = sum((Z_test - Z_pred_lasso).^2);

lasso_R2(fold) = 1 - SS_res/SS_tot;

lasso_RMSE(fold) = sqrt(mean((Z_test - Z_pred_lasso).^2));

X_search_local = linspace(min(X_train), max(X_train), 100);

Y_search_local = linspace(min(Y_train), max(Y_train), 100);

max_Z_val = -inf;

for i = 1:length(X_search_local)

for j = 1:length(Y_search_local)

Z_val = intercept + coef(1)*X_search_local(i) + coef(2)*Y_search_local(j) + ...

coef(3)*X_search_local(i)^2 + coef(4)*Y_search_local(j)^2 + ...

coef(5)*X_search_local(i)*Y_search_local(j);

if Z_val > max_Z_val

max_Z_val = Z_val;

best_X_lasso(fold) = X_search_local(i);

best_Y_lasso(fold) = Y_search_local(j);

end

end

end

% ======= Method 3: Random Forest =======

rf_model = TreeBagger(50, [X_train, Y_train], Z_train, 'Method', 'regression');

Z_pred_rf = predict(rf_model, [X_test, Y_test]);

SS_res = sum((Z_test - Z_pred_rf).^2);

rf_R2(fold) = 1 - SS_res/SS_tot;

rf_RMSE(fold) = sqrt(mean((Z_test - Z_pred_rf).^2));

X_search_local = linspace(min(X_train), max(X_train), 100);

Y_search_local = linspace(min(Y_train), max(Y_train), 100);

max_Z_val = -inf;

for i = 1:length(X_search_local)

for j = 1:length(Y_search_local)

Z_val = predict(rf_model, [X_search_local(i), Y_search_local(j)]);

if Z_val > max_Z_val && ~isnan(Z_val)

max_Z_val = Z_val;

best_X_rf(fold) = X_search_local(i);

best_Y_rf(fold) = Y_search_local(j);

end

end

end

% ======= Method 4: Neural Network =======

X_min_train = min(X_train); X_max_train = max(X_train);

Y_min_train = min(Y_train); Y_max_train = max(Y_train);

Z_min_train = min(Z_train); Z_max_train = max(Z_train);

X_norm_train = (X_train - X_min_train) / (X_max_train - X_min_train);

Y_norm_train = (Y_train - Y_min_train) / (Y_max_train - Y_min_train);

Z_norm_train = (Z_train - Z_min_train) / (Z_max_train - Z_min_train);

rng(fold * 100);

net = feedforwardnet(5);

net.trainFcn = 'trainlm';

net.trainParam.epochs = 200;

net.trainParam.showWindow = false;

inputs_train = [X_norm_train'; Y_norm_train'];

targets_train = Z_norm_train';

[net_trained, ~] = train(net, inputs_train, targets_train);

X_norm_test = (X_test - X_min_train) / (X_max_train - X_min_train);

Y_norm_test = (Y_test - Y_min_train) / (Y_max_train - Y_min_train);

inputs_test = [X_norm_test'; Y_norm_test'];

Z_norm_pred = net_trained(inputs_test);

Z_pred_nn = Z_norm_pred' * (Z_max_train - Z_min_train) + Z_min_train;

SS_res = sum((Z_test - Z_pred_nn).^2);

nn_R2(fold) = 1 - SS_res/SS_tot;

nn_RMSE(fold) = sqrt(mean((Z_test - Z_pred_nn).^2));

X_search_local = linspace(min(X_train), max(X_train), 100);

Y_search_local = linspace(min(Y_train), max(Y_train), 100);

max_Z_val = -inf;

for i = 1:length(X_search_local)

for j = 1:length(Y_search_local)

X_norm_val = (X_search_local(i) - X_min_train) / (X_max_train - X_min_train);

Y_norm_val = (Y_search_local(j) - Y_min_train) / (Y_max_train - Y_min_train);

Z_pred_val = net_trained([X_norm_val; Y_norm_val]) * ...

(Z_max_train - Z_min_train) + Z_min_train;

if Z_pred_val > max_Z_val

max_Z_val = Z_pred_val;

best_X_nn(fold) = X_search_local(i);

best_Y_nn(fold) = Y_search_local(j);

end

end

end

fprintf('QR: R²=%.4f, RMSE=%.4f, Optimal X=%.4f\n', ...

quadratic_R2(fold), quadratic_RMSE(fold), best_X_quadratic(fold));

fprintf('Lasso: R²=%.4f, RMSE=%.4f, Optimal X=%.4f\n', ...

lasso_R2(fold), lasso_RMSE(fold), best_X_lasso(fold));

fprintf('RF: R²=%.4f, RMSE=%.4f, Optimal X=%.4f\n', ...

rf_R2(fold), rf_RMSE(fold), best_X_rf(fold));

fprintf('NN: R²=%.4f, RMSE=%.4f, Optimal X=%.4f\n', ...

nn_R2(fold), nn_RMSE(fold), best_X_nn(fold));

end

% ==================== 3.5 Temporal Block Cross-Validation ====================

fprintf('\n\n=== Temporal Block Cross-Validation (Temporal Block CV) ===\n');

fprintf('Description: Data is divided into 5 consecutive blocks in collection order, each block as one fold\n');

fprintf('Purpose: Control the impact of time-series autocorrelation on performance estimation\n');

block_size = floor(n / K);

indices_block = zeros(n, 1);

for fold = 1:K

start_idx = (fold-1) * block_size + 1;

end_idx = min(fold * block_size, n);

indices_block(start_idx:end_idx) = fold;

end

if end_idx < n

indices_block(end_idx+1:end) = K;

end

fprintf('Sample ranges per fold (in collection order):\n');

for fold = 1:K

idx_range = find(indices_block == fold);

fprintf(' Fold %d: Samples %d - %d (%d samples)\n', fold, min(idx_range), max(idx_range), length(idx_range));

end

% Storage for temporal block CV results

quadratic_R2_block = zeros(K, 1);

quadratic_RMSE_block = zeros(K, 1);

lasso_R2_block = zeros(K, 1);

lasso_RMSE_block = zeros(K, 1);

rf_R2_block = zeros(K, 1);

rf_RMSE_block = zeros(K, 1);

nn_R2_block = zeros(K, 1);

nn_RMSE_block = zeros(K, 1);

best_X_quadratic_block = zeros(K, 1);

best_X_lasso_block = zeros(K, 1);

best_X_rf_block = zeros(K, 1);

best_X_nn_block = zeros(K, 1);

best_Y_quadratic_block = zeros(K, 1);

best_Y_lasso_block = zeros(K, 1);

best_Y_rf_block = zeros(K, 1);

best_Y_nn_block = zeros(K, 1);

for fold = 1:K

fprintf('\n--- Fold %d Temporal Block Cross-Validation ---\n', fold);

test_idx = (indices_block == fold);

train_idx = ~test_idx;

X_train = X(train_idx);

Y_train = Y(train_idx);

Z_train = Z(train_idx);

X_test = X(test_idx);

Y_test = Y(test_idx);

Z_test = Z(test_idx);

SS_tot = sum((Z_test - mean(Z_test)).^2);

% ======= Method 1: Quadratic Regression =======

A_train = [ones(size(X_train)), X_train, Y_train, ...

X_train.^2, Y_train.^2, X_train.*Y_train];

coefficients = A_train \ Z_train;

A_test = [ones(size(X_test)), X_test, Y_test, ...

X_test.^2, Y_test.^2, X_test.*Y_test];

Z_pred_quad = A_test * coefficients;

SS_res = sum((Z_test - Z_pred_quad).^2);

quadratic_R2_block(fold) = 1 - SS_res/SS_tot;

quadratic_RMSE_block(fold) = sqrt(mean((Z_test - Z_pred_quad).^2));

B0c = coefficients(1); B1c = coefficients(2); B2c = coefficients(3);

B3c = coefficients(4); B4c = coefficients(5); B5c = coefficients(6);

M = [2*B3c, B5c; B5c, 2*B4c];

RHS = [-B1c; -B2c];

if abs(det(M)) > 1e-10

XY_opt = M \ RHS;

best_X_quadratic_block(fold) = XY_opt(1);

best_Y_quadratic_block(fold) = XY_opt(2);

else

X_search_local = linspace(min(X_train), max(X_train), 100);

Y_search_local = linspace(min(Y_train), max(Y_train), 100);

max_Z_val = -inf;

for i = 1:length(X_search_local)

for j = 1:length(Y_search_local)

Z_val = B0c + B1c*X_search_local(i) + B2c*Y_search_local(j) + ...

B3c*X_search_local(i)^2 + B4c*Y_search_local(j)^2 + ...

B5c*X_search_local(i)*Y_search_local(j);

if Z_val > max_Z_val

max_Z_val = Z_val;

best_X_quadratic_block(fold) = X_search_local(i);

best_Y_quadratic_block(fold) = Y_search_local(j);

end

end

end

end

% ======= Method 2: Lasso Regression =======

A_train_lasso = [X_train, Y_train, X_train.^2, Y_train.^2, X_train.*Y_train];

A_test_lasso = [X_test, Y_test, X_test.^2, Y_test.^2, X_test.*Y_test];

[B, info] = lasso(A_train_lasso, Z_train, 'CV', 5);

idx = info.IndexMinMSE;

intercept = info.Intercept(idx);

coef = B(:, idx);

Z_pred_lasso = intercept + A_test_lasso * coef;

SS_res = sum((Z_test - Z_pred_lasso).^2);

lasso_R2_block(fold) = 1 - SS_res/SS_tot;

lasso_RMSE_block(fold) = sqrt(mean((Z_test - Z_pred_lasso).^2));

X_search_local = linspace(min(X_train), max(X_train), 100);

Y_search_local = linspace(min(Y_train), max(Y_train), 100);

max_Z_val = -inf;

for i = 1:length(X_search_local)

for j = 1:length(Y_search_local)

Z_val = intercept + coef(1)*X_search_local(i) + coef(2)*Y_search_local(j) + ...

coef(3)*X_search_local(i)^2 + coef(4)*Y_search_local(j)^2 + ...

coef(5)*X_search_local(i)*Y_search_local(j);

if Z_val > max_Z_val

max_Z_val = Z_val;

best_X_lasso_block(fold) = X_search_local(i);

best_Y_lasso_block(fold) = Y_search_local(j);

end

end

end

% ======= Method 3: Random Forest =======

rf_model = TreeBagger(50, [X_train, Y_train], Z_train, 'Method', 'regression');

Z_pred_rf = predict(rf_model, [X_test, Y_test]);

SS_res = sum((Z_test - Z_pred_rf).^2);

rf_R2_block(fold) = 1 - SS_res/SS_tot;

rf_RMSE_block(fold) = sqrt(mean((Z_test - Z_pred_rf).^2));

X_search_local = linspace(min(X_train), max(X_train), 100);

Y_search_local = linspace(min(Y_train), max(Y_train), 100);

max_Z_val = -inf;

for i = 1:length(X_search_local)

for j = 1:length(Y_search_local)

Z_val = predict(rf_model, [X_search_local(i), Y_search_local(j)]);

if Z_val > max_Z_val && ~isnan(Z_val)

max_Z_val = Z_val;

best_X_rf_block(fold) = X_search_local(i);

best_Y_rf_block(fold) = Y_search_local(j);

end

end

end

% ======= Method 4: Neural Network =======

X_min_train = min(X_train); X_max_train = max(X_train);

Y_min_train = min(Y_train); Y_max_train = max(Y_train);

Z_min_train = min(Z_train); Z_max_train = max(Z_train);

X_norm_train = (X_train - X_min_train) / (X_max_train - X_min_train);

Y_norm_train = (Y_train - Y_min_train) / (Y_max_train - Y_min_train);

Z_norm_train = (Z_train - Z_min_train) / (Z_max_train - Z_min_train);

rng(fold * 100 + 42);

net = feedforwardnet(5);

net.trainFcn = 'trainlm';

net.trainParam.epochs = 200;

net.trainParam.showWindow = false;

inputs_train = [X_norm_train'; Y_norm_train'];

targets_train = Z_norm_train';

[net_trained, ~] = train(net, inputs_train, targets_train);

X_norm_test = (X_test - X_min_train) / (X_max_train - X_min_train);

Y_norm_test = (Y_test - Y_min_train) / (Y_max_train - Y_min_train);

inputs_test = [X_norm_test'; Y_norm_test'];

Z_norm_pred = net_trained(inputs_test);

Z_pred_nn = Z_norm_pred' * (Z_max_train - Z_min_train) + Z_min_train;

SS_res = sum((Z_test - Z_pred_nn).^2);

nn_R2_block(fold) = 1 - SS_res/SS_tot;

nn_RMSE_block(fold) = sqrt(mean((Z_test - Z_pred_nn).^2));

X_search_local = linspace(min(X_train), max(X_train), 100);

Y_search_local = linspace(min(Y_train), max(Y_train), 100);

max_Z_val = -inf;

for i = 1:length(X_search_local)

for j = 1:length(Y_search_local)

X_norm_val = (X_search_local(i) - X_min_train) / (X_max_train - X_min_train);

Y_norm_val = (Y_search_local(j) - Y_min_train) / (Y_max_train - Y_min_train);

Z_pred_val = net_trained([X_norm_val; Y_norm_val]) * ...

(Z_max_train - Z_min_train) + Z_min_train;

if Z_pred_val > max_Z_val

max_Z_val = Z_pred_val;

best_X_nn_block(fold) = X_search_local(i);

best_Y_nn_block(fold) = Y_search_local(j);

end

end

end

fprintf('QR: R²=%.4f, RMSE=%.4f, Optimal X=%.4f\n', ...

quadratic_R2_block(fold), quadratic_RMSE_block(fold), best_X_quadratic_block(fold));

fprintf('Lasso: R²=%.4f, RMSE=%.4f, Optimal X=%.4f\n', ...

lasso_R2_block(fold), lasso_RMSE_block(fold), best_X_lasso_block(fold));

fprintf('RF: R²=%.4f, RMSE=%.4f, Optimal X=%.4f\n', ...

rf_R2_block(fold), rf_RMSE_block(fold), best_X_rf_block(fold));

fprintf('NN: R²=%.4f, RMSE=%.4f, Optimal X=%.4f\n', ...

nn_R2_block(fold), nn_RMSE_block(fold), best_X_nn_block(fold));

end

% ==================== 3.6 Spatial Block Cross-Validation ====================

fprintf('\n\n=== Spatial Block Cross-Validation (Spatial Block CV) ===\n');

fprintf('Description: Data is divided into 5 consecutive spatial blocks by the value range of solid density X\n');

fprintf('Purpose: Control the impact of spatial proximity on performance estimation\n');

% Sort by X value and partition into blocks

[X_sorted, sort_idx_X] = sort(X);

block_size = floor(n / K);

indices_spatial = zeros(n, 1);

for fold = 1:K

start_idx = (fold-1) * block_size + 1;

end_idx = min(fold * block_size, n);

for i = start_idx:end_idx

indices_spatial(sort_idx_X(i)) = fold;

end

end

if end_idx < n

for i = end_idx+1:n

indices_spatial(sort_idx_X(i)) = K;

end

end

fprintf('Solid density X ranges per fold (partitioned by value range):\n');

for fold = 1:K

idx_range = find(indices_spatial == fold);

x_min = min(X(idx_range));

x_max = max(X(idx_range));

fprintf(' Fold %d: X ∈ [%.4f, %.4f] (%d samples)\n', fold, x_min, x_max, length(idx_range));

end

% Storage for spatial block CV results

quadratic_R2_spatial = zeros(K, 1);

quadratic_RMSE_spatial = zeros(K, 1);

lasso_R2_spatial = zeros(K, 1);

lasso_RMSE_spatial = zeros(K, 1);

rf_R2_spatial = zeros(K, 1);

rf_RMSE_spatial = zeros(K, 1);

nn_R2_spatial = zeros(K, 1);

nn_RMSE_spatial = zeros(K, 1);

best_X_quadratic_spatial = zeros(K, 1);

best_X_lasso_spatial = zeros(K, 1);

best_X_rf_spatial = zeros(K, 1);

best_X_nn_spatial = zeros(K, 1);

best_Y_quadratic_spatial = zeros(K, 1);

best_Y_lasso_spatial = zeros(K, 1);

best_Y_rf_spatial = zeros(K, 1);

best_Y_nn_spatial = zeros(K, 1);

for fold = 1:K

fprintf('\n--- Fold %d Spatial Block Cross-Validation ---\n', fold);

test_idx = (indices_spatial == fold);

train_idx = ~test_idx;

X_train = X(train_idx);

Y_train = Y(train_idx);

Z_train = Z(train_idx);

X_test = X(test_idx);

Y_test = Y(test_idx);

Z_test = Z(test_idx);

SS_tot = sum((Z_test - mean(Z_test)).^2);

% ======= Method 1: Quadratic Regression =======

A_train = [ones(size(X_train)), X_train, Y_train, ...

X_train.^2, Y_train.^2, X_train.*Y_train];

coefficients = A_train \ Z_train;

A_test = [ones(size(X_test)), X_test, Y_test, ...

X_test.^2, Y_test.^2, X_test.*Y_test];

Z_pred_quad = A_test * coefficients;

SS_res = sum((Z_test - Z_pred_quad).^2);

quadratic_R2_spatial(fold) = 1 - SS_res/SS_tot;

quadratic_RMSE_spatial(fold) = sqrt(mean((Z_test - Z_pred_quad).^2));

B0c = coefficients(1); B1c = coefficients(2); B2c = coefficients(3);

B3c = coefficients(4); B4c = coefficients(5); B5c = coefficients(6);

M = [2*B3c, B5c; B5c, 2*B4c];

RHS = [-B1c; -B2c];

if abs(det(M)) > 1e-10

XY_opt = M \ RHS;

best_X_quadratic_spatial(fold) = XY_opt(1);

best_Y_quadratic_spatial(fold) = XY_opt(2);

else

X_search_local = linspace(min(X_train), max(X_train), 100);

Y_search_local = linspace(min(Y_train), max(Y_train), 100);

max_Z_val = -inf;

for i = 1:length(X_search_local)

for j = 1:length(Y_search_local)

Z_val = B0c + B1c*X_search_local(i) + B2c*Y_search_local(j) + ...

B3c*X_search_local(i)^2 + B4c*Y_search_local(j)^2 + ...

B5c*X_search_local(i)*Y_search_local(j);

if Z_val > max_Z_val

max_Z_val = Z_val;

best_X_quadratic_spatial(fold) = X_search_local(i);

best_Y_quadratic_spatial(fold) = Y_search_local(j);

end

end

end

end

% ======= Method 2: Lasso Regression =======

A_train_lasso = [X_train, Y_train, X_train.^2, Y_train.^2, X_train.*Y_train];

A_test_lasso = [X_test, Y_test, X_test.^2, Y_test.^2, X_test.*Y_test];

[B, info] = lasso(A_train_lasso, Z_train, 'CV', 5);

idx = info.IndexMinMSE;

intercept = info.Intercept(idx);

coef = B(:, idx);

Z_pred_lasso = intercept + A_test_lasso * coef;

SS_res = sum((Z_test - Z_pred_lasso).^2);

lasso_R2_spatial(fold) = 1 - SS_res/SS_tot;

lasso_RMSE_spatial(fold) = sqrt(mean((Z_test - Z_pred_lasso).^2));

X_search_local = linspace(min(X_train), max(X_train), 100);

Y_search_local = linspace(min(Y_train), max(Y_train), 100);

max_Z_val = -inf;

for i = 1:length(X_search_local)

for j = 1:length(Y_search_local)

Z_val = intercept + coef(1)*X_search_local(i) + coef(2)*Y_search_local(j) + ...

coef(3)*X_search_local(i)^2 + coef(4)*Y_search_local(j)^2 + ...

coef(5)*X_search_local(i)*Y_search_local(j);

if Z_val > max_Z_val

max_Z_val = Z_val;

best_X_lasso_spatial(fold) = X_search_local(i);

best_Y_lasso_spatial(fold) = Y_search_local(j);

end

end

end

% ======= Method 3: Random Forest =======

rf_model = TreeBagger(50, [X_train, Y_train], Z_train, 'Method', 'regression');

Z_pred_rf = predict(rf_model, [X_test, Y_test]);

SS_res = sum((Z_test - Z_pred_rf).^2);

rf_R2_spatial(fold) = 1 - SS_res/SS_tot;

rf_RMSE_spatial(fold) = sqrt(mean((Z_test - Z_pred_rf).^2));

X_search_local = linspace(min(X_train), max(X_train), 100);

Y_search_local = linspace(min(Y_train), max(Y_train), 100);

max_Z_val = -inf;

for i = 1:length(X_search_local)

for j = 1:length(Y_search_local)

Z_val = predict(rf_model, [X_search_local(i), Y_search_local(j)]);

if Z_val > max_Z_val && ~isnan(Z_val)

max_Z_val = Z_val;

best_X_rf_spatial(fold) = X_search_local(i);

best_Y_rf_spatial(fold) = Y_search_local(j);

end

end

end

% ======= Method 4: Neural Network =======

X_min_train = min(X_train); X_max_train = max(X_train);

Y_min_train = min(Y_train); Y_max_train = max(Y_train);

Z_min_train = min(Z_train); Z_max_train = max(Z_train);

X_norm_train = (X_train - X_min_train) / (X_max_train - X_min_train);

Y_norm_train = (Y_train - Y_min_train) / (Y_max_train - Y_min_train);

Z_norm_train = (Z_train - Z_min_train) / (Z_max_train - Z_min_train);

rng(fold * 100 + 84);

net = feedforwardnet(5);

net.trainFcn = 'trainlm';

net.trainParam.epochs = 200;

net.trainParam.showWindow = false;

inputs_train = [X_norm_train'; Y_norm_train'];

targets_train = Z_norm_train';

[net_trained, ~] = train(net, inputs_train, targets_train);

X_norm_test = (X_test - X_min_train) / (X_max_train - X_min_train);

Y_norm_test = (Y_test - Y_min_train) / (Y_max_train - Y_min_train);

inputs_test = [X_norm_test'; Y_norm_test'];

Z_norm_pred = net_trained(inputs_test);

Z_pred_nn = Z_norm_pred' * (Z_max_train - Z_min_train) + Z_min_train;

SS_res = sum((Z_test - Z_pred_nn).^2);

nn_R2_spatial(fold) = 1 - SS_res/SS_tot;

nn_RMSE_spatial(fold) = sqrt(mean((Z_test - Z_pred_nn).^2));

X_search_local = linspace(min(X_train), max(X_train), 100);

Y_search_local = linspace(min(Y_train), max(Y_train), 100);

max_Z_val = -inf;

for i = 1:length(X_search_local)

for j = 1:length(Y_search_local)

X_norm_val = (X_search_local(i) - X_min_train) / (X_max_train - X_min_train);

Y_norm_val = (Y_search_local(j) - Y_min_train) / (Y_max_train - Y_min_train);

Z_pred_val = net_trained([X_norm_val; Y_norm_val]) * ...

(Z_max_train - Z_min_train) + Z_min_train;

if Z_pred_val > max_Z_val

max_Z_val = Z_pred_val;

best_X_nn_spatial(fold) = X_search_local(i);

best_Y_nn_spatial(fold) = Y_search_local(j);

end

end

end

fprintf('QR: R²=%.4f, RMSE=%.4f, Optimal X=%.4f\n', ...

quadratic_R2_spatial(fold), quadratic_RMSE_spatial(fold), best_X_quadratic_spatial(fold));

fprintf('Lasso: R²=%.4f, RMSE=%.4f, Optimal X=%.4f\n', ...

lasso_R2_spatial(fold), lasso_RMSE_spatial(fold), best_X_lasso_spatial(fold));

fprintf('RF: R²=%.4f, RMSE=%.4f, Optimal X=%.4f\n', ...

rf_R2_spatial(fold), rf_RMSE_spatial(fold), best_X_rf_spatial(fold));

fprintf('NN: R²=%.4f, RMSE=%.4f, Optimal X=%.4f\n', ...

nn_R2_spatial(fold), nn_RMSE_spatial(fold), best_X_nn_spatial(fold));

end

% ==================== 4. Statistical Analysis of Results ====================

fprintf('\n\n=== Statistical Analysis of Four Methods (Random CV) ===\n');

fprintf('\n1. Fitting Performance Comparison (5-fold CV):\n');

fprintf('--------------------------------------------------------------------\n');

fprintf('Method	Mean R²	R² Std	Mean RMSE	RMSE Std\n');

fprintf('--------------------------------------------------------------------\n');

fprintf('Quadratic	%.4f	%.4f	%.4f	%.4f\n', ...

mean(quadratic_R2), std(quadratic_R2), mean(quadratic_RMSE), std(quadratic_RMSE));

fprintf('Lasso	%.4f	%.4f	%.4f	%.4f\n', ...

mean(lasso_R2), std(lasso_R2), mean(lasso_RMSE), std(lasso_RMSE));

fprintf('Random Forest	%.4f	%.4f	%.4f	%.4f\n', ...

mean(rf_R2), std(rf_R2), mean(rf_RMSE), std(rf_RMSE));

fprintf('Neural Network	%.4f	%.4f	%.4f	%.4f\n', ...

mean(nn_R2), std(nn_R2), mean(nn_RMSE), std(nn_RMSE));

fprintf('--------------------------------------------------------------------\n');

fprintf('\n2. Optimal Solid Density Stability Comparison (Random CV):\n');

fprintf('Quadratic optimal X: %.4f ± %.4f (CV=%.2f%%)\n', mean(best_X_quadratic), std(best_X_quadratic), std(best_X_quadratic)/mean(best_X_quadratic)*100);

fprintf('Lasso optimal X: %.4f ± %.4f (CV=%.2f%%)\n', mean(best_X_lasso), std(best_X_lasso), std(best_X_lasso)/mean(best_X_lasso)*100);

fprintf('Random Forest optimal X: %.4f ± %.4f (CV=%.2f%%)\n', mean(best_X_rf), std(best_X_rf), std(best_X_rf)/mean(best_X_rf)*100);

fprintf('Neural Network optimal X: %.4f ± %.4f (CV=%.2f%%)\n', mean(best_X_nn), std(best_X_nn), std(best_X_nn)/mean(best_X_nn)*100);

% ==================== 4.5 Three CV Strategy Results Comparison ====================

fprintf('\n\n=== Comparison of Three Cross-Validation Strategies ===\n');

fprintf('Description:\n');

fprintf(' - Random CV: Randomly shuffled folds (baseline)\n');

fprintf(' - Temporal Block CV: Consecutive order folds (controls time dependence)\n');

fprintf(' - Spatial Block CV: X-value range folds (controls spatial dependence)\n');

fprintf('====================================================================\n');

fprintf('Model	| Metric | Random CV	| Temporal Block	| Spatial Block	\n');

fprintf('====================================================================\n');

print_compare3 = @(name, metric, val_random, val_block, val_spatial) ...

fprintf('%s	| %s	| %.4f ± %.4f | %.4f ± %.4f | %.4f ± %.4f\n', ...

name, metric, mean(val_random), std(val_random), mean(val_block), std(val_block), mean(val_spatial), std(val_spatial));

print_compare3('QR', 'R²', quadratic_R2, quadratic_R2_block, quadratic_R2_spatial);

print_compare3('Lasso', 'R²', lasso_R2, lasso_R2_block, lasso_R2_spatial);

print_compare3('RF', 'R²', rf_R2, rf_R2_block, rf_R2_spatial);

print_compare3('NN', 'R²', nn_R2, nn_R2_block, nn_R2_spatial);

print_compare3('QR', 'RMSE', quadratic_RMSE, quadratic_RMSE_block, quadratic_RMSE_spatial);

print_compare3('Lasso', 'RMSE', lasso_RMSE, lasso_RMSE_block, lasso_RMSE_spatial);

print_compare3('RF', 'RMSE', rf_RMSE, rf_RMSE_block, rf_RMSE_spatial);

print_compare3('NN', 'RMSE', nn_RMSE, nn_RMSE_block, nn_RMSE_spatial);

fprintf('====================================================================\n');

fprintf('\nModel ranking by strategy (by R²):\n');

fprintf(' Random CV: RF=%.4f, NN=%.4f, QR=%.4f, Lasso=%.4f\n', ...

mean(rf_R2), mean(nn_R2), mean(quadratic_R2), mean(lasso_R2));

fprintf(' Temporal CV: RF=%.4f, NN=%.4f, QR=%.4f, Lasso=%.4f\n', ...

mean(rf_R2_block), mean(nn_R2_block), mean(quadratic_R2_block), mean(lasso_R2_block));

fprintf(' Spatial CV: RF=%.4f, NN=%.4f, QR=%.4f, Lasso=%.4f\n', ...

mean(rf_R2_spatial), mean(nn_R2_spatial), mean(quadratic_R2_spatial), mean(lasso_R2_spatial));

% Generate bar chart comparing three CV strategies

figure('Position', [100, 100, 900, 600]);

bar_data = [mean(quadratic_R2), mean(lasso_R2), mean(rf_R2), mean(nn_R2);

mean(quadratic_R2_block), mean(lasso_R2_block), mean(rf_R2_block), mean(nn_R2_block);

mean(quadratic_R2_spatial), mean(lasso_R2_spatial), mean(rf_R2_spatial), mean(nn_R2_spatial)];

bar(bar_data);

set(gca, 'XTickLabel', {'QR', 'Lasso', 'RF', 'NN'});

legend('Random CV', 'Temporal Block CV', 'Spatial Block CV', 'Location', 'best');

ylabel('Mean R²');

title('Magenta Ink: R² Comparison Across Three CV Strategies');

grid on;

saveas(gcf, 'Magenta_Ink_CV_Comparison_Three.png');

fprintf('\nThree-strategy comparison figure saved: Magenta_Ink_CV_Comparison_Three.png\n');

% ==================== 5. Statistical Significance Test (Paired t-test) ====================

fprintf('\n=== Statistical Significance Test (Paired t-test) - Based on Random CV ===\n');

diff_R2_NN_QR = nn_R2 - quadratic_R2;

diff_R2_RF_QR = rf_R2 - quadratic_R2;

diff_R2_RF_NN = rf_R2 - nn_R2;

diff_R2_Lasso_QR = lasso_R2 - quadratic_R2;

[~, p_NN_QR] = ttest(diff_R2_NN_QR);

fprintf('\nNN vs QR:\n');

fprintf(' Mean R² difference: +%.4f\n', mean(diff_R2_NN_QR));

fprintf(' p = %.4f', p_NN_QR);

if p_NN_QR < 0.05

fprintf(' (Significant difference)\n');

else

fprintf(' (No significant difference)\n');

end

[~, p_RF_QR] = ttest(diff_R2_RF_QR);

fprintf('\nRF vs QR:\n');

fprintf(' Mean R² difference: +%.4f\n', mean(diff_R2_RF_QR));

fprintf(' p = %.4f', p_RF_QR);

if p_RF_QR < 0.05

fprintf(' (Significant difference)\n');

else

fprintf(' (No significant difference)\n');

end

[~, p_RF_NN] = ttest(diff_R2_RF_NN);

fprintf('\nRF vs NN:\n');

fprintf(' Mean R² difference: +%.4f\n', mean(diff_R2_RF_NN));

fprintf(' p = %.4f', p_RF_NN);

if p_RF_NN < 0.05

fprintf(' (Significant difference)\n');

else

fprintf(' (No significant difference)\n');

end

fprintf('\nLasso vs QR:\n');

fprintf(' Mean R² difference: +%.4f (No significant difference, p > 0.05)\n', mean(diff_R2_Lasso_QR));

% ==================== 6. Response Surface Analysis ====================

fprintf('\n=== Generating Response Surfaces ===\n');

% Grid points

xg = linspace(1.3, 1.7, 100);

yg = linspace(14, 26, 100);

[Xg, Yg] = meshgrid(xg, yg);

% Calculate actual data range

in_range = (Xg >= min(X)) & (Xg <= max(X)) & ...

(Yg >= min(Y)) & (Yg <= max(Y));

% 6.1 QR response surface

Zg_qr = B0 + B1*Xg + B2*Yg + B3*Xg.^2 + B4*Yg.^2 + B5*Xg.*Yg;

Zg_qr(Zg_qr < 0) = NaN;

Zg_qr(~in_range) = NaN;

% 6.2 Lasso response surface

Zg_lasso = intercept_lasso + coef_lasso(1)*Xg + coef_lasso(2)*Yg + ...

coef_lasso(3)*Xg.^2 + coef_lasso(4)*Yg.^2 + coef_lasso(5)*Xg.*Yg;

Zg_lasso(Zg_lasso < 0) = NaN;

Zg_lasso(~in_range) = NaN;

% 6.3 RF response surface

Zg_rf = predict(rf_full, [Xg(:), Yg(:)]);

Zg_rf = reshape(Zg_rf, size(Xg));

Zg_rf(Zg_rf < 0) = NaN;

Zg_rf(~in_range) = NaN;

% 6.4 NN response surface

Xg_norm = (Xg - X_min) / (X_max - X_min);

Yg_norm = (Yg - Y_min) / (Y_max - Y_min);

Zg_nn = net_full([Xg_norm(:)'; Yg_norm(:)'])';

Zg_nn = reshape(Zg_nn, size(Xg)) * (Z_max - Z_min) + Z_min;

Zg_nn(Zg_nn < 0) = NaN;

Zg_nn(~in_range) = NaN;

% Generate 2×2 comparison figure

figure('Position', [100, 100, 1200, 1000], 'Name', 'Magenta Ink: Four Model Response Surface Comparison');

subplot(2,2,1);

surf(Xg, Yg, Zg_qr, 'EdgeColor', 'none', 'FaceAlpha', 0.8);

hold on;

scatter3(X, Y, Z, 20, 'k', 'filled', 'MarkerFaceAlpha', 0.3);

title(sprintf('(a) Quadratic Regression QR (R²=%.4f)', R2_quad_full));

xlabel('Solid Density X'); ylabel('Dot Gain Y'); zlabel('Contrast Z');

colormap(jet); colorbar; view(45, 30); grid on;

zlim([min(Z), max(Z)]);

subplot(2,2,2);

surf(Xg, Yg, Zg_lasso, 'EdgeColor', 'none', 'FaceAlpha', 0.8);

hold on;

scatter3(X, Y, Z, 20, 'k', 'filled', 'MarkerFaceAlpha', 0.3);

title(sprintf('(b) Lasso Regression (R²=%.4f)', R2_lasso_full));

xlabel('Solid Density X'); ylabel('Dot Gain Y'); zlabel('Contrast Z');

colormap(jet); colorbar; view(45, 30); grid on;

zlim([min(Z), max(Z)]);

subplot(2,2,3);

surf(Xg, Yg, Zg_rf, 'EdgeColor', 'none', 'FaceAlpha', 0.8);

hold on;

scatter3(X, Y, Z, 20, 'k', 'filled', 'MarkerFaceAlpha', 0.3);

title(sprintf('(c) Random Forest RF (R²=%.4f)', R2_rf_full));

xlabel('Solid Density X'); ylabel('Dot Gain Y'); zlabel('Contrast Z');

colormap(jet); colorbar; view(45, 30); grid on;

zlim([min(Z), max(Z)]);

subplot(2,2,4);

surf(Xg, Yg, Zg_nn, 'EdgeColor', 'none', 'FaceAlpha', 0.8);

hold on;

scatter3(X, Y, Z, 20, 'k', 'filled', 'MarkerFaceAlpha', 0.3);

title(sprintf('(d) Neural Network MLP (R²=%.4f)', R2_nn_full));

xlabel('Solid Density X'); ylabel('Dot Gain Y'); zlabel('Contrast Z');

colormap(jet); colorbar; view(45, 30); grid on;

zlim([min(Z), max(Z)]);

sgtitle('Magenta Ink: Four Model Response Surface Comparison');

saveas(gcf, 'Magenta_Ink_ResponseSurfaces.png');

fprintf(' Response surface figure saved: Magenta_Ink_ResponseSurfaces.png\n');

% ==================== 7. Cross-Validation Analysis ====================

fprintf('\n=== Optimal Point Cross-Validation Analysis ===\n');

Z_nn_at_qr = interp2(Xg, Yg, Zg_nn, X_opt_quad, Y_opt_quad);

Z_rf_at_qr = interp2(Xg, Yg, Zg_rf, X_opt_quad, Y_opt_quad);

Z_lasso_at_qr = interp2(Xg, Yg, Zg_lasso, X_opt_quad, Y_opt_quad);

Z_qr_at_nn = interp2(Xg, Yg, Zg_qr, best_X_nn_full, best_Y_nn_full);

Z_rf_at_nn = interp2(Xg, Yg, Zg_rf, best_X_nn_full, best_Y_nn_full);

Z_lasso_at_nn = interp2(Xg, Yg, Zg_lasso, best_X_nn_full, best_Y_nn_full);

Z_qr_at_rf = interp2(Xg, Yg, Zg_qr, best_X_rf_full, best_Y_rf_full);

Z_nn_at_rf = interp2(Xg, Yg, Zg_nn, best_X_rf_full, best_Y_rf_full);

Z_lasso_at_rf = interp2(Xg, Yg, Zg_lasso, best_X_rf_full, best_Y_rf_full);

fprintf('\nQR optimum (X=%.4f, Y=%.4f, Z=%.4f):\n', X_opt_quad, Y_opt_quad, Z_opt_quad);

fprintf(' Prediction on NN: Z = %.4f (Diff: %+.4f)\n', Z_nn_at_qr, Z_nn_at_qr - Z_opt_quad);

fprintf(' Prediction on RF: Z = %.4f (Diff: %+.4f)\n', Z_rf_at_qr, Z_rf_at_qr - Z_opt_quad);

fprintf(' Prediction on Lasso: Z = %.4f (Diff: %+.4f)\n', Z_lasso_at_qr, Z_lasso_at_qr - Z_opt_quad);

fprintf('\nNN optimum (X=%.4f, Y=%.4f, Z=%.4f):\n', best_X_nn_full, best_Y_nn_full, max_Z_nn);

fprintf(' Prediction on QR: Z = %.4f (Diff: %+.4f)\n', Z_qr_at_nn, Z_qr_at_nn - max_Z_nn);

fprintf(' Prediction on RF: Z = %.4f (Diff: %+.4f)\n', Z_rf_at_nn, Z_rf_at_nn - max_Z_nn);

fprintf(' Prediction on Lasso: Z = %.4f (Diff: %+.4f)\n', Z_lasso_at_nn, Z_lasso_at_nn - max_Z_nn);

fprintf('\nRF optimum (X=%.4f, Y=%.4f, Z=%.4f):\n', best_X_rf_full, best_Y_rf_full, max_Z_rf);

fprintf(' Prediction on QR: Z = %.4f (Diff: %+.4f)\n', Z_qr_at_rf, Z_qr_at_rf - max_Z_rf);

fprintf(' Prediction on NN: Z = %.4f (Diff: %+.4f)\n', Z_nn_at_rf, Z_nn_at_rf - max_Z_rf);

fprintf(' Prediction on Lasso: Z = %.4f (Diff: %+.4f)\n', Z_lasso_at_rf, Z_lasso_at_rf - max_Z_rf);

% ==================== 8. Residual Analysis ====================

fprintf('\n=== Residual Analysis ===\n');

residuals_qr = Z - Z_pred_quad_full;

residuals_lasso = Z - Z_pred_lasso_full;

residuals_rf = Z - Z_pred_rf_full;

residuals_nn = Z - Z_pred_nn_full;

figure('Position', [100, 100, 800, 800], 'Name', 'Magenta Ink Residual Analysis');

subplot(2,2,1);

scatter(Z_pred_quad_full, residuals_qr, 'b.');

hold on;

plot([min(Z_pred_quad_full), max(Z_pred_quad_full)], [0, 0], 'r--', 'LineWidth', 1);

xlabel('Predicted Value'); ylabel('Residual'); title('(a) QR Residual Plot');

grid on;

subplot(2,2,2);

scatter(Z_pred_lasso_full, residuals_lasso, 'g.');

hold on;

plot([min(Z_pred_lasso_full), max(Z_pred_lasso_full)], [0, 0], 'r--', 'LineWidth', 1);

xlabel('Predicted Value'); ylabel('Residual'); title('(b) Lasso Residual Plot');

grid on;

subplot(2,2,3);

scatter(Z_pred_rf_full, residuals_rf, 'r.');

hold on;

plot([min(Z_pred_rf_full), max(Z_pred_rf_full)], [0, 0], 'r--', 'LineWidth', 1);

xlabel('Predicted Value'); ylabel('Residual'); title('(c) RF Residual Plot');

grid on;

subplot(2,2,4);

scatter(Z_pred_nn_full, residuals_nn, 'm.');

hold on;

plot([min(Z_pred_nn_full), max(Z_pred_nn_full)], [0, 0], 'r--', 'LineWidth', 1);

xlabel('Predicted Value'); ylabel('Residual'); title('(d) NN Residual Plot');

grid on;

sgtitle('Magenta Ink: Four Model Residual Distribution Comparison');

saveas(gcf, 'Magenta_Ink_Residuals.png');

% ==================== 9. Generate Comparison Charts ====================

fprintf('\n=== Generating Comparison Charts ===\n');

% Figure 1: R² boxplot

figure('Position', [100, 100, 600, 500], 'Name', 'Magenta Ink R² Boxplot');

boxplot([quadratic_R2, lasso_R2, rf_R2, nn_R2], 'Labels', {'QR', 'Lasso', 'RF', 'NN'});

ylabel('Coefficient of Determination R²');

title('Magenta Ink: R² Distribution Comparison (Random 5-fold CV)');

grid on;

saveas(gcf, 'Magenta_Ink_R2_Boxplot.png');

% Figure 2: RMSE boxplot

figure('Position', [150, 150, 600, 500], 'Name', 'Magenta Ink RMSE Boxplot');

boxplot([quadratic_RMSE, lasso_RMSE, rf_RMSE, nn_RMSE], 'Labels', {'QR', 'Lasso', 'RF', 'NN'});

ylabel('Root Mean Square Error RMSE');

title('Magenta Ink: RMSE Distribution Comparison (Random 5-fold CV)');

grid on;

saveas(gcf, 'Magenta_Ink_RMSE_Boxplot.png');

% Figure 3: Optimal X stability line chart (Random CV)

figure('Position', [200, 200, 700, 500], 'Name', 'Magenta Ink Optimal X Stability');

hold on;

plot(1:K, best_X_quadratic, 'bo-', 'LineWidth', 2, 'MarkerSize', 8, 'DisplayName', 'QR');

plot(1:K, best_X_lasso, 'go-', 'LineWidth', 2, 'MarkerSize', 8, 'DisplayName', 'Lasso');

plot(1:K, best_X_rf, 'ro-', 'LineWidth', 2, 'MarkerSize', 8, 'DisplayName', 'RF');

plot(1:K, best_X_nn, 'mo-', 'LineWidth', 2, 'MarkerSize', 8, 'DisplayName', 'NN');

xlabel('Cross-Validation Fold'); ylabel('Optimal Solid Density X');

title('Magenta Ink: Optimal Solid Density Variation Across Folds (Random CV)');

legend('Location', 'best'); grid on;

xlim([0.5, 5.5]);

saveas(gcf, 'Magenta_Ink_Xopt_Stability.png');

% Figure 4: Mean performance bar chart

figure('Position', [250, 250, 800, 500], 'Name', 'Magenta Ink Mean Performance Comparison');

subplot(1,2,1);

mean_R2 = [mean(quadratic_R2), mean(lasso_R2), mean(rf_R2), mean(nn_R2)];

std_R2 = [std(quadratic_R2), std(lasso_R2), std(rf_R2), std(nn_R2)];

bar(1:4, mean_R2, 0.6, 'FaceColor', [0.5 0.5 0.8]);

hold on;

errorbar(1:4, mean_R2, std_R2, 'k.', 'LineWidth', 1.5, 'MarkerSize', 15);

set(gca, 'XTickLabel', {'QR', 'Lasso', 'RF', 'NN'});

ylabel('Mean R²'); title('Magenta Ink: Mean R² Comparison');

ylim([0.7, 1.0]); grid on;

subplot(1,2,2);

mean_RMSE = [mean(quadratic_RMSE), mean(lasso_RMSE), mean(rf_RMSE), mean(nn_RMSE)];

std_RMSE = [std(quadratic_RMSE), std(lasso_RMSE), std(rf_RMSE), std(nn_RMSE)];

bar(1:4, mean_RMSE, 0.6, 'FaceColor', [0.8 0.5 0.5]);

hold on;

errorbar(1:4, mean_RMSE, std_RMSE, 'k.', 'LineWidth', 1.5, 'MarkerSize', 15);

set(gca, 'XTickLabel', {'QR', 'Lasso', 'RF', 'NN'});

ylabel('Mean RMSE'); title('Magenta Ink: Mean RMSE Comparison');

grid on;

sgtitle('Magenta Ink: Four Model Mean Performance Comparison (Random 5-fold CV)');

saveas(gcf, 'Magenta_Ink_Performance_Bar.png');

fprintf('\n=== Magenta Ink Analysis Complete! ===\n');

fprintf('Generated image files:\n');

fprintf(' Magenta_Ink_Autocorrelation.png\n');

fprintf(' Magenta_Ink_CV_Comparison_Three.png <- New: Three CV strategy comparison\n');

fprintf(' Magenta_Ink_ResponseSurfaces.png\n');

fprintf(' Magenta_Ink_Residuals.png\n');

fprintf(' Magenta_Ink_R2_Boxplot.png\n');

fprintf(' Magenta_Ink_RMSE_Boxplot.png\n');

fprintf(' Magenta_Ink_Xopt_Stability.png\n');

fprintf(' Magenta_Ink_Performance_Bar.png\n');

## ## 4. Yellow Ink

% ==================== 1. Data Preparation ====================

fprintf('=== Yellow Ink Data Preparation ===\n');

% Yellow ink data - format: [solid density X, dot gain Y, contrast Z]

DATA = [

0.85	10.01	18.73

0.85	10.03	18.76

0.85	10.02	18.79

0.86	10.04	18.83

0.86	10.01	18.79

0.86	10.03	18.81

0.87	10.04	18.85

0.87	10.05	18.84

0.87	10.05	18.92

0.88	10.03	19.47

0.88	10.05	19.45

0.88	10.07	19.65

0.89	11.68	20.83

0.89	11.99	21.43

0.89	12.09	21.63

0.90	13.35	22.82

0.90	15.60	23.31

0.90	15.61	23.80

0.91	10.10	23.84

0.91	13.33	24.84

0.91	14.13	23.83

0.91	14.14	23.81

0.91	15.61	23.82

0.91	15.63	23.81

0.92	10.12	25.81

0.92	10.12	25.21

0.92	13.34	25.91

0.92	13.36	25.82

0.92	14.13	26.69

0.92	14.12	26.82

0.92	15.62	27.84

0.92	15.62	27.82

0.93	11.01	28.59

0.93	11.02	28.65

0.93	10.13	28.78

0.93	13.35	28.89

0.93	14.11	29.49

0.94	11.02	29.87

0.94	14.12	30.14

0.94	14.31	30.54

0.94	16.22	29.96

0.95	11.04	31.65

0.95	11.03	31.84

0.95	14.30	32.03

0.95	14.30	32.04

0.95	14.33	32.03

0.95	15.26	32.02

0.95	15.27	32.02

0.95	16.22	32.04

0.96	11.03	32.13

0.96	14.32	32.15

0.96	15.28	32.13

0.96	16.24	32.14

0.97	14.35	32.21

0.97	15.26	32.23

0.97	16.23	32.22

0.97	17.80	32.20

0.98	15.29	32.33

0.98	17.81	32.34

0.98	17.77	32.33

0.98	16.41	32.34

0.99	17.79	32.98

0.99	17.78	33.04

0.99	16.34	33.03

1.00	17.91	33.11

1.00	16.39	33.10

1.00	16.36	33.04

1.01	12.26	33.10

1.01	12.25	33.13

1.01	16.37	33.12

1.02	12.26	33.16

1.02	12.27	33.18

1.02	16.35	33.19

1.03	12.27	33.79

1.03	16.61	33.81

1.03	17.37	33.82

1.04	12.28	31.80

1.04	13.12	29.65

1.04	16.65	30.89

1.04	18.57	30.47

1.04	17.38	30.67

1.04	17.35	30.53

1.05	13.12	29.67

1.05	13.11	28.94

1.05	17.37	28.87

1.05	17.36	29.59

1.06	13.10	29.68

1.06	13.13	28.46

1.06	16.61	27.81

1.07	16.69	26.99

1.07	18.56	26.58

1.08	18.59	26.53

1.08	17.10	25.99

1.09	14.25	24.84

1.09	17.11	24.99

1.09	19.02	24.72

1.10	14.26	23.41

1.10	14.26	23.37

1.10	17.12	22.32

1.11	15.23	22.26

1.11	14.27	21.44

1.11	14.27	21.42

1.12	15.26	22.27

1.12	15.26	22.24

1.12	14.26	21.43

1.13	19.04	20.85

1.13	15.27	22.28

1.14	16.01	20.86

1.15	15.24	22.23

1.15	15.21	20.74

];

X = DATA(:, 1);

Y = DATA(:, 2);

Z = DATA(:, 3);

n = length(X);

fprintf('Sample size: %d\n', n);

fprintf('Solid density range: %.2f - %.2f\n', min(X), max(X));

fprintf('Dot gain range: %.2f - %.2f\n', min(Y), max(Y));

fprintf('Contrast range: %.2f - %.2f\n\n', min(Z), max(Z));

% ===== Yellow ink specific: MLP hidden layer neuron count set to 3 =====

% Based on hyperparameter search, the optimal hidden layer neuron count for yellow ink is 3

% 5-neuron training was unstable, 3-neuron is stable and has optimal performance

fprintf('=== Yellow Ink MLP Configuration ===\n');

fprintf('Using optimal hidden layer neuron count: 3 (based on hyperparameter search)\n');

fprintf('5-neuron training unstable, 3-neuron stable with optimal performance\n\n');

% ==================== 1.5 Autocorrelation Analysis ====================

fprintf('\n=== Autocorrelation Analysis ===\n');

% Manual autocorrelation calculation

max_lag = 20;

Z_mean = mean(Z);

Z_var = var(Z);

acf_manual = zeros(max_lag + 1, 1);

for lag = 0:max_lag

if lag == 0

acf_manual(lag+1) = 1;

else

sum_cov = 0;

for i = 1:(n-lag)

sum_cov = sum_cov + (Z(i) - Z_mean) * (Z(i+lag) - Z_mean);

end

acf_manual(lag+1) = sum_cov / ((n-lag) * Z_var);

end

end

lags_manual = (0:max_lag)';

conf = 1.96 / sqrt(n);

figure('Position', [100, 100, 800, 500]);

stem(lags_manual, acf_manual, 'filled', 'LineWidth', 1.5);

hold on;

plot([0, max_lag], [conf, conf], 'r--', 'LineWidth', 1);

plot([0, max_lag], [-conf, -conf], 'r--', 'LineWidth', 1);

xlabel('Lag'); ylabel('Autocorrelation Coefficient');

title('Autocorrelation Function of Yellow Ink Contrast Values');

grid on;

saveas(gcf, 'Yellow_Ink_Autocorrelation.png');

fprintf('Lag-1 autocorrelation coefficient: %.4f\n', acf_manual(2));

% ==================== 2. Full-Data Analysis (Baseline) ====================

fprintf('=== Full-Data Baseline Analysis ===\n');

% 2.1 Quadratic regression full-data fitting

A_full = [ones(size(X)), X, Y, X.^2, Y.^2, X.*Y];

coeff_full = A_full \ Z;

Z_pred_quad_full = A_full * coeff_full;

R2_quad_full = 1 - sum((Z - Z_pred_quad_full).^2) / sum((Z - mean(Z)).^2);

B0 = coeff_full(1); B1 = coeff_full(2); B2 = coeff_full(3);

B3 = coeff_full(4); B4 = coeff_full(5); B5 = coeff_full(6);

M = [2*B3, B5; B5, 2*B4];

RHS = [-B1; -B2];

if abs(det(M)) > 1e-10

XY_opt_quad = M \ RHS;

X_opt_quad = XY_opt_quad(1);

Y_opt_quad = XY_opt_quad(2);

else

X_search = linspace(min(X), max(X), 100);

Y_search = linspace(min(Y), max(Y), 100);

max_Z = -inf;

for i = 1:length(X_search)

for j = 1:length(Y_search)

Z_val = B0 + B1*X_search(i) + B2*Y_search(j) + ...

B3*X_search(i)^2 + B4*Y_search(j)^2 + ...

B5*X_search(i)*Y_search(j);

if Z_val > max_Z

max_Z = Z_val;

X_opt_quad = X_search(i);

Y_opt_quad = Y_search(j);

end

end

end

end

Z_opt_quad = B0 + B1*X_opt_quad + B2*Y_opt_quad + B3*X_opt_quad^2 + ...

B4*Y_opt_quad^2 + B5*X_opt_quad*Y_opt_quad;

fprintf('\nQuadratic Regression (full data):\n');

fprintf(' R² = %.4f\n', R2_quad_full);

fprintf(' Optimal solid density X = %.4f\n', X_opt_quad);

fprintf(' Optimal dot gain Y = %.4f\n', Y_opt_quad);

fprintf(' Predicted maximum contrast Z = %.4f\n', Z_opt_quad);

% 2.2 Lasso full-data fitting

fprintf('\nLasso Regression (full data):\n');

A_lasso_full = [X, Y, X.^2, Y.^2, X.*Y];

[B_lasso, info_lasso] = lasso(A_lasso_full, Z, 'CV', 5);

idx_lasso = info_lasso.IndexMinMSE;

intercept_lasso = info_lasso.Intercept(idx_lasso);

coef_lasso = B_lasso(:, idx_lasso);

Z_pred_lasso_full = intercept_lasso + A_lasso_full * coef_lasso;

R2_lasso_full = 1 - sum((Z - Z_pred_lasso_full).^2) / sum((Z - mean(Z)).^2);

% Grid search for Lasso optimal point (yellow ink range)

X_search = linspace(0.85, 1.15, 100);

Y_search = linspace(10, 20, 100);

max_Z_lasso = -inf;

best_X_lasso_full = NaN;

best_Y_lasso_full = NaN;

for i = 1:length(X_search)

for j = 1:length(Y_search)

Z_val = intercept_lasso + coef_lasso(1)*X_search(i) + coef_lasso(2)*Y_search(j) + ...

coef_lasso(3)*X_search(i)^2 + coef_lasso(4)*Y_search(j)^2 + ...

coef_lasso(5)*X_search(i)*Y_search(j);

if Z_val > max_Z_lasso

max_Z_lasso = Z_val;

best_X_lasso_full = X_search(i);

best_Y_lasso_full = Y_search(j);

end

end

end

fprintf(' R² = %.4f\n', R2_lasso_full);

fprintf(' Optimal solid density X = %.4f\n', best_X_lasso_full);

fprintf(' Optimal dot gain Y = %.4f\n', best_Y_lasso_full);

fprintf(' Predicted maximum contrast Z = %.4f\n', max_Z_lasso);

% 2.3 Random forest full-data fitting

fprintf('\nRandom Forest (full data):\n');

rf_full = TreeBagger(100, [X, Y], Z, 'Method', 'regression');

Z_pred_rf_full = predict(rf_full, [X, Y]);

R2_rf_full = 1 - sum((Z - Z_pred_rf_full).^2) / sum((Z - mean(Z)).^2);

% Grid search for RF optimal point

X_search = linspace(0.85, 1.15, 100);

Y_search = linspace(10, 20, 100);

max_Z_rf = -inf;

best_X_rf_full = NaN;

best_Y_rf_full = NaN;

for i = 1:length(X_search)

for j = 1:length(Y_search)

Z_val = predict(rf_full, [X_search(i), Y_search(j)]);

if Z_val > max_Z_rf && ~isnan(Z_val)

max_Z_rf = Z_val;

best_X_rf_full = X_search(i);

best_Y_rf_full = Y_search(j);

end

end

end

fprintf(' R² = %.4f\n', R2_rf_full);

fprintf(' Optimal solid density X = %.4f\n', best_X_rf_full);

fprintf(' Optimal dot gain Y = %.4f\n', best_Y_rf_full);

fprintf(' Predicted maximum contrast Z = %.4f\n', max_Z_rf);

% 2.4 Neural network full-data fitting (using 3 hidden layer neurons)

fprintf('\nNeural Network (full data):\n');

fprintf(' Using optimal hidden layer neuron count: 3\n');

X_min = min(X); X_max = max(X);

Y_min = min(Y); Y_max = max(Y);

Z_min = min(Z); Z_max = max(Z);

if X_max == X_min; X_max = X_min + eps; end

if Y_max == Y_min; Y_max = Y_min + eps; end

if Z_max == Z_min; Z_max = Z_min + eps; end

X_norm = (X - X_min) / (X_max - X_min);

Y_norm = (Y - Y_min) / (Y_max - Y_min);

Z_norm = (Z - Z_min) / (Z_max - Z_min);

rng(42);

net_full = feedforwardnet(3);

net_full.trainFcn = 'trainlm';

net_full.trainParam.epochs = 300;

net_full.trainParam.showWindow = false;

inputs_full = [X_norm'; Y_norm'];

targets_full = Z_norm';

[net_full, ~] = train(net_full, inputs_full, targets_full);

Z_pred_norm = net_full(inputs_full);

Z_pred_nn_full = Z_pred_norm' * (Z_max - Z_min) + Z_min;

R2_nn_full = 1 - sum((Z - Z_pred_nn_full).^2) / sum((Z - mean(Z)).^2);

X_search = linspace(0.85, 1.15, 100);

Y_search = linspace(10, 20, 100);

max_Z_nn = -inf;

best_X_nn_full = NaN;

best_Y_nn_full = NaN;

for i = 1:length(X_search)

for j = 1:length(Y_search)

X_norm_val = (X_search(i) - X_min) / (X_max - X_min);

Y_norm_val = (Y_search(j) - Y_min) / (Y_max - Y_min);

Z_pred_val = net_full([X_norm_val; Y_norm_val]) * (Z_max - Z_min) + Z_min;

if Z_pred_val > max_Z_nn

max_Z_nn = Z_pred_val;

best_X_nn_full = X_search(i);

best_Y_nn_full = Y_search(j);

end

end

end

fprintf(' R² = %.4f\n', R2_nn_full);

fprintf(' Optimal solid density X = %.4f\n', best_X_nn_full);

fprintf(' Optimal dot gain Y = %.4f\n', best_Y_nn_full);

fprintf(' Predicted maximum contrast Z = %.4f\n', max_Z_nn);

% ==================== 3. Random 5-Fold Cross-Validation ====================

fprintf('\n\n=== Random 5-Fold Cross-Validation Method Comparison ===\n');

K = 5;

rng(42);

indices = crossvalind('Kfold', n, K);

% Storage for 4 methods' results (random CV)

quadratic_R2 = zeros(K, 1);

quadratic_RMSE = zeros(K, 1);

lasso_R2 = zeros(K, 1);

lasso_RMSE = zeros(K, 1);

rf_R2 = zeros(K, 1);

rf_RMSE = zeros(K, 1);

nn_R2 = zeros(K, 1);

nn_RMSE = zeros(K, 1);

best_X_quadratic = zeros(K, 1);

best_X_lasso = zeros(K, 1);

best_X_rf = zeros(K, 1);

best_X_nn = zeros(K, 1);

best_Y_quadratic = zeros(K, 1);

best_Y_lasso = zeros(K, 1);

best_Y_rf = zeros(K, 1);

best_Y_nn = zeros(K, 1);

for fold = 1:K

fprintf('\n--- Fold %d Cross-Validation ---\n', fold);

test_idx = (indices == fold);

train_idx = ~test_idx;

X_train = X(train_idx);

Y_train = Y(train_idx);

Z_train = Z(train_idx);

X_test = X(test_idx);

Y_test = Y(test_idx);

Z_test = Z(test_idx);

SS_tot = sum((Z_test - mean(Z_test)).^2);

% Calculate training set convex hull (for restricting predictions)

train_unique = unique([X_train, Y_train], 'rows');

X_train_uniq = train_unique(:,1);

Y_train_uniq = train_unique(:,2);

if size(train_unique, 1) >= 3

DT_train = delaunayTriangulation(X_train_uniq, Y_train_uniq);

[K_train, ~] = convexHull(DT_train);

conv_hull_train = [X_train_uniq(K_train), Y_train_uniq(K_train)];

else

conv_hull_train = [min(X_train), min(Y_train); max(X_train), max(Y_train)];

end

% ======= Method 1: Quadratic Regression =======

A_train = [ones(size(X_train)), X_train, Y_train, ...

X_train.^2, Y_train.^2, X_train.*Y_train];

coefficients = A_train \ Z_train;

A_test = [ones(size(X_test)), X_test, Y_test, ...

X_test.^2, Y_test.^2, X_test.*Y_test];

Z_pred_quad = A_test * coefficients;

SS_res = sum((Z_test - Z_pred_quad).^2);

quadratic_R2(fold) = 1 - SS_res/SS_tot;

quadratic_RMSE(fold) = sqrt(mean((Z_test - Z_pred_quad).^2));

B0c = coefficients(1); B1c = coefficients(2); B2c = coefficients(3);

B3c = coefficients(4); B4c = coefficients(5); B5c = coefficients(6);

M = [2*B3c, B5c; B5c, 2*B4c];

RHS = [-B1c; -B2c];

if abs(det(M)) > 1e-10

XY_opt = M \ RHS;

best_X_quadratic(fold) = XY_opt(1);

best_Y_quadratic(fold) = XY_opt(2);

else

X_search_local = linspace(min(X_train), max(X_train), 100);

Y_search_local = linspace(min(Y_train), max(Y_train), 100);

max_Z_val = -inf;

for i = 1:length(X_search_local)

for j = 1:length(Y_search_local)

Z_val = B0c + B1c*X_search_local(i) + B2c*Y_search_local(j) + ...

B3c*X_search_local(i)^2 + B4c*Y_search_local(j)^2 + ...

B5c*X_search_local(i)*Y_search_local(j);

if Z_val > max_Z_val

max_Z_val = Z_val;

best_X_quadratic(fold) = X_search_local(i);

best_Y_quadratic(fold) = Y_search_local(j);

end

end

end

end

% ======= Method 2: Lasso Regression =======

A_train_lasso = [X_train, Y_train, X_train.^2, Y_train.^2, X_train.*Y_train];

A_test_lasso = [X_test, Y_test, X_test.^2, Y_test.^2, X_test.*Y_test];

[B, info] = lasso(A_train_lasso, Z_train, 'CV', 5);

idx = info.IndexMinMSE;

intercept = info.Intercept(idx);

coef = B(:, idx);

Z_pred_lasso = intercept + A_test_lasso * coef;

SS_res = sum((Z_test - Z_pred_lasso).^2);

lasso_R2(fold) = 1 - SS_res/SS_tot;

lasso_RMSE(fold) = sqrt(mean((Z_test - Z_pred_lasso).^2));

X_search_local = linspace(min(X_train), max(X_train), 100);

Y_search_local = linspace(min(Y_train), max(Y_train), 100);

max_Z_val = -inf;

for i = 1:length(X_search_local)

for j = 1:length(Y_search_local)

Z_val = intercept + coef(1)*X_search_local(i) + coef(2)*Y_search_local(j) + ...

coef(3)*X_search_local(i)^2 + coef(4)*Y_search_local(j)^2 + ...

coef(5)*X_search_local(i)*Y_search_local(j);

if Z_val > max_Z_val

max_Z_val = Z_val;

best_X_lasso(fold) = X_search_local(i);

best_Y_lasso(fold) = Y_search_local(j);

end

end

end

% ======= Method 3: Random Forest =======

rf_model = TreeBagger(100, [X_train, Y_train], Z_train, 'Method', 'regression');

Z_pred_rf = predict(rf_model, [X_test, Y_test]);

SS_res = sum((Z_test - Z_pred_rf).^2);

rf_R2(fold) = 1 - SS_res/SS_tot;

rf_RMSE(fold) = sqrt(mean((Z_test - Z_pred_rf).^2));

X_search_local = linspace(min(X_train), max(X_train), 100);

Y_search_local = linspace(min(Y_train), max(Y_train), 100);

max_Z_val = -inf;

for i = 1:length(X_search_local)

for j = 1:length(Y_search_local)

Z_val = predict(rf_model, [X_search_local(i), Y_search_local(j)]);

if Z_val > max_Z_val && ~isnan(Z_val)

max_Z_val = Z_val;

best_X_rf(fold) = X_search_local(i);

best_Y_rf(fold) = Y_search_local(j);

end

end

end

% ======= Method 4: Neural Network (using 3 hidden layer neurons) =======

X_min_train = min(X_train); X_max_train = max(X_train);

Y_min_train = min(Y_train); Y_max_train = max(Y_train);

Z_min_train = min(Z_train); Z_max_train = max(Z_train);

if X_max_train == X_min_train; X_max_train = X_min_train + eps; end

if Y_max_train == Y_min_train; Y_max_train = Y_min_train + eps; end

if Z_max_train == Z_min_train; Z_max_train = Z_min_train + eps; end

X_norm_train = (X_train - X_min_train) / (X_max_train - X_min_train);

Y_norm_train = (Y_train - Y_min_train) / (Y_max_train - Y_min_train);

Z_norm_train = (Z_train - Z_min_train) / (Z_max_train - Z_min_train);

rng(fold * 100);

net = feedforwardnet(3);

net.trainFcn = 'trainlm';

net.trainParam.epochs = 200;

net.trainParam.showWindow = false;

inputs_train = [X_norm_train'; Y_norm_train'];

targets_train = Z_norm_train';

[net_trained, ~] = train(net, inputs_train, targets_train);

X_norm_test = (X_test - X_min_train) / (X_max_train - X_min_train);

Y_norm_test = (Y_test - Y_min_train) / (Y_max_train - Y_min_train);

inputs_test = [X_norm_test'; Y_norm_test'];

Z_norm_pred = net_trained(inputs_test);

Z_pred_nn = Z_norm_pred' * (Z_max_train - Z_min_train) + Z_min_train;

SS_res = sum((Z_test - Z_pred_nn).^2);

nn_R2(fold) = 1 - SS_res/SS_tot;

nn_RMSE(fold) = sqrt(mean((Z_test - Z_pred_nn).^2));

X_search_local = linspace(min(X_train), max(X_train), 100);

Y_search_local = linspace(min(Y_train), max(Y_train), 100);

max_Z_val = -inf;

for i = 1:length(X_search_local)

for j = 1:length(Y_search_local)

% Check if within convex hull

if size(conv_hull_train, 1) >= 3

if ~inpolygon(X_search_local(i), Y_search_local(j), conv_hull_train(:,1), conv_hull_train(:,2))

continue;

end

end

X_norm_val = (X_search_local(i) - X_min_train) / (X_max_train - X_min_train);

Y_norm_val = (Y_search_local(j) - Y_min_train) / (Y_max_train - Y_min_train);

Z_pred_val = net_trained([X_norm_val; Y_norm_val]) * ...

(Z_max_train - Z_min_train) + Z_min_train;

if Z_pred_val > max_Z_val

max_Z_val = Z_pred_val;

best_X_nn(fold) = X_search_local(i);

best_Y_nn(fold) = Y_search_local(j);

end

end

end

fprintf('QR: R²=%.4f, RMSE=%.4f, Optimal X=%.4f\n', ...

quadratic_R2(fold), quadratic_RMSE(fold), best_X_quadratic(fold));

fprintf('Lasso: R²=%.4f, RMSE=%.4f, Optimal X=%.4f\n', ...

lasso_R2(fold), lasso_RMSE(fold), best_X_lasso(fold));

fprintf('RF: R²=%.4f, RMSE=%.4f, Optimal X=%.4f\n', ...

rf_R2(fold), rf_RMSE(fold), best_X_rf(fold));

fprintf('NN: R²=%.4f, RMSE=%.4f, Optimal X=%.4f\n', ...

nn_R2(fold), nn_RMSE(fold), best_X_nn(fold));

end

% ==================== 3.5 Temporal Block Cross-Validation ====================

fprintf('\n\n=== Temporal Block Cross-Validation (Temporal Block CV) ===\n');

fprintf('Description: Data is divided into 5 consecutive blocks in collection order, each block as one fold\n');

fprintf('Purpose: Control the impact of time-series autocorrelation on performance estimation\n');

block_size = floor(n / K);

indices_block = zeros(n, 1);

for fold = 1:K

start_idx = (fold-1) * block_size + 1;

end_idx = min(fold * block_size, n);

indices_block(start_idx:end_idx) = fold;

end

if end_idx < n

indices_block(end_idx+1:end) = K;

end

fprintf('Sample ranges per fold (in collection order):\n');

for fold = 1:K

idx_range = find(indices_block == fold);

fprintf(' Fold %d: Samples %d - %d (%d samples)\n', fold, min(idx_range), max(idx_range), length(idx_range));

end

% Storage for temporal block CV results

quadratic_R2_block = zeros(K, 1);

quadratic_RMSE_block = zeros(K, 1);

lasso_R2_block = zeros(K, 1);

lasso_RMSE_block = zeros(K, 1);

rf_R2_block = zeros(K, 1);

rf_RMSE_block = zeros(K, 1);

nn_R2_block = zeros(K, 1);

nn_RMSE_block = zeros(K, 1);

best_X_quadratic_block = zeros(K, 1);

best_X_lasso_block = zeros(K, 1);

best_X_rf_block = zeros(K, 1);

best_X_nn_block = zeros(K, 1);

best_Y_quadratic_block = zeros(K, 1);

best_Y_lasso_block = zeros(K, 1);

best_Y_rf_block = zeros(K, 1);

best_Y_nn_block = zeros(K, 1);

for fold = 1:K

fprintf('\n--- Fold %d Temporal Block Cross-Validation ---\n', fold);

test_idx = (indices_block == fold);

train_idx = ~test_idx;

X_train = X(train_idx);

Y_train = Y(train_idx);

Z_train = Z(train_idx);

X_test = X(test_idx);

Y_test = Y(test_idx);

Z_test = Z(test_idx);

SS_tot = sum((Z_test - mean(Z_test)).^2);

% Calculate training set convex hull

train_unique = unique([X_train, Y_train], 'rows');

X_train_uniq = train_unique(:,1);

Y_train_uniq = train_unique(:,2);

if size(train_unique, 1) >= 3

DT_train = delaunayTriangulation(X_train_uniq, Y_train_uniq);

[K_train, ~] = convexHull(DT_train);

conv_hull_train = [X_train_uniq(K_train), Y_train_uniq(K_train)];

else

conv_hull_train = [min(X_train), min(Y_train); max(X_train), max(Y_train)];

end

% ======= Method 1: Quadratic Regression =======

A_train = [ones(size(X_train)), X_train, Y_train, ...

X_train.^2, Y_train.^2, X_train.*Y_train];

coefficients = A_train \ Z_train;

A_test = [ones(size(X_test)), X_test, Y_test, ...

X_test.^2, Y_test.^2, X_test.*Y_test];

Z_pred_quad = A_test * coefficients;

SS_res = sum((Z_test - Z_pred_quad).^2);

quadratic_R2_block(fold) = 1 - SS_res/SS_tot;

quadratic_RMSE_block(fold) = sqrt(mean((Z_test - Z_pred_quad).^2));

B0c = coefficients(1); B1c = coefficients(2); B2c = coefficients(3);

B3c = coefficients(4); B4c = coefficients(5); B5c = coefficients(6);

M = [2*B3c, B5c; B5c, 2*B4c];

RHS = [-B1c; -B2c];

if abs(det(M)) > 1e-10

XY_opt = M \ RHS;

best_X_quadratic_block(fold) = XY_opt(1);

best_Y_quadratic_block(fold) = XY_opt(2);

else

X_search_local = linspace(min(X_train), max(X_train), 100);

Y_search_local = linspace(min(Y_train), max(Y_train), 100);

max_Z_val = -inf;

for i = 1:length(X_search_local)

for j = 1:length(Y_search_local)

Z_val = B0c + B1c*X_search_local(i) + B2c*Y_search_local(j) + ...

B3c*X_search_local(i)^2 + B4c*Y_search_local(j)^2 + ...

B5c*X_search_local(i)*Y_search_local(j);

if Z_val > max_Z_val

max_Z_val = Z_val;

best_X_quadratic_block(fold) = X_search_local(i);

best_Y_quadratic_block(fold) = Y_search_local(j);

end

end

end

end

% ======= Method 2: Lasso Regression =======

A_train_lasso = [X_train, Y_train, X_train.^2, Y_train.^2, X_train.*Y_train];

A_test_lasso = [X_test, Y_test, X_test.^2, Y_test.^2, X_test.*Y_test];

[B, info] = lasso(A_train_lasso, Z_train, 'CV', 5);

idx = info.IndexMinMSE;

intercept = info.Intercept(idx);

coef = B(:, idx);

Z_pred_lasso = intercept + A_test_lasso * coef;

SS_res = sum((Z_test - Z_pred_lasso).^2);

lasso_R2_block(fold) = 1 - SS_res/SS_tot;

lasso_RMSE_block(fold) = sqrt(mean((Z_test - Z_pred_lasso).^2));

X_search_local = linspace(min(X_train), max(X_train), 100);

Y_search_local = linspace(min(Y_train), max(Y_train), 100);

max_Z_val = -inf;

for i = 1:length(X_search_local)

for j = 1:length(Y_search_local)

Z_val = intercept + coef(1)*X_search_local(i) + coef(2)*Y_search_local(j) + ...

coef(3)*X_search_local(i)^2 + coef(4)*Y_search_local(j)^2 + ...

coef(5)*X_search_local(i)*Y_search_local(j);

if Z_val > max_Z_val

max_Z_val = Z_val;

best_X_lasso_block(fold) = X_search_local(i);

best_Y_lasso_block(fold) = Y_search_local(j);

end

end

end

% ======= Method 3: Random Forest =======

rf_model = TreeBagger(100, [X_train, Y_train], Z_train, 'Method', 'regression');

Z_pred_rf = predict(rf_model, [X_test, Y_test]);

SS_res = sum((Z_test - Z_pred_rf).^2);

rf_R2_block(fold) = 1 - SS_res/SS_tot;

rf_RMSE_block(fold) = sqrt(mean((Z_test - Z_pred_rf).^2));

X_search_local = linspace(min(X_train), max(X_train), 100);

Y_search_local = linspace(min(Y_train), max(Y_train), 100);

max_Z_val = -inf;

for i = 1:length(X_search_local)

for j = 1:length(Y_search_local)

Z_val = predict(rf_model, [X_search_local(i), Y_search_local(j)]);

if Z_val > max_Z_val && ~isnan(Z_val)

max_Z_val = Z_val;

best_X_rf_block(fold) = X_search_local(i);

best_Y_rf_block(fold) = Y_search_local(j);

end

end

end

% ======= Method 4: Neural Network (using 3 hidden layer neurons) =======

X_min_train = min(X_train); X_max_train = max(X_train);

Y_min_train = min(Y_train); Y_max_train = max(Y_train);

Z_min_train = min(Z_train); Z_max_train = max(Z_train);

if X_max_train == X_min_train; X_max_train = X_min_train + eps; end

if Y_max_train == Y_min_train; Y_max_train = Y_min_train + eps; end

if Z_max_train == Z_min_train; Z_max_train = Z_min_train + eps; end

X_norm_train = (X_train - X_min_train) / (X_max_train - X_min_train);

Y_norm_train = (Y_train - Y_min_train) / (Y_max_train - Y_min_train);

Z_norm_train = (Z_train - Z_min_train) / (Z_max_train - Z_min_train);

rng(fold * 100 + 42);

net = feedforwardnet(3);

net.trainFcn = 'trainlm';

net.trainParam.epochs = 200;

net.trainParam.showWindow = false;

inputs_train = [X_norm_train'; Y_norm_train'];

targets_train = Z_norm_train';

[net_trained, ~] = train(net, inputs_train, targets_train);

X_norm_test = (X_test - X_min_train) / (X_max_train - X_min_train);

Y_norm_test = (Y_test - Y_min_train) / (Y_max_train - Y_min_train);

inputs_test = [X_norm_test'; Y_norm_test'];

Z_norm_pred = net_trained(inputs_test);

Z_pred_nn = Z_norm_pred' * (Z_max_train - Z_min_train) + Z_min_train;

SS_res = sum((Z_test - Z_pred_nn).^2);

nn_R2_block(fold) = 1 - SS_res/SS_tot;

nn_RMSE_block(fold) = sqrt(mean((Z_test - Z_pred_nn).^2));

X_search_local = linspace(min(X_train), max(X_train), 100);

Y_search_local = linspace(min(Y_train), max(Y_train), 100);

max_Z_val = -inf;

for i = 1:length(X_search_local)

for j = 1:length(Y_search_local)

if size(conv_hull_train, 1) >= 3

if ~inpolygon(X_search_local(i), Y_search_local(j), conv_hull_train(:,1), conv_hull_train(:,2))

continue;

end

end

X_norm_val = (X_search_local(i) - X_min_train) / (X_max_train - X_min_train);

Y_norm_val = (Y_search_local(j) - Y_min_train) / (Y_max_train - Y_min_train);

Z_pred_val = net_trained([X_norm_val; Y_norm_val]) * ...

(Z_max_train - Z_min_train) + Z_min_train;

if Z_pred_val > max_Z_val

max_Z_val = Z_pred_val;

best_X_nn_block(fold) = X_search_local(i);

best_Y_nn_block(fold) = Y_search_local(j);

end

end

end

fprintf('QR: R²=%.4f, RMSE=%.4f, Optimal X=%.4f\n', ...

quadratic_R2_block(fold), quadratic_RMSE_block(fold), best_X_quadratic_block(fold));

fprintf('Lasso: R²=%.4f, RMSE=%.4f, Optimal X=%.4f\n', ...

lasso_R2_block(fold), lasso_RMSE_block(fold), best_X_lasso_block(fold));

fprintf('RF: R²=%.4f, RMSE=%.4f, Optimal X=%.4f\n', ...

rf_R2_block(fold), rf_RMSE_block(fold), best_X_rf_block(fold));

fprintf('NN: R²=%.4f, RMSE=%.4f, Optimal X=%.4f\n', ...

nn_R2_block(fold), nn_RMSE_block(fold), best_X_nn_block(fold));

end

% ==================== 3.6 Spatial Block Cross-Validation ====================

fprintf('\n\n=== Spatial Block Cross-Validation (Spatial Block CV) ===\n');

fprintf('Description: Data is divided into 5 consecutive spatial blocks by the value range of solid density X\n');

fprintf('Purpose: Control the impact of spatial proximity on performance estimation\n');

% Sort by X value and partition into blocks

[X_sorted, sort_idx_X] = sort(X);

block_size = floor(n / K);

indices_spatial = zeros(n, 1);

for fold = 1:K

start_idx = (fold-1) * block_size + 1;

end_idx = min(fold * block_size, n);

for i = start_idx:end_idx

indices_spatial(sort_idx_X(i)) = fold;

end

end

if end_idx < n

for i = end_idx+1:n

indices_spatial(sort_idx_X(i)) = K;

end

end

fprintf('Solid density X ranges per fold (partitioned by value range):\n');

for fold = 1:K

idx_range = find(indices_spatial == fold);

x_min = min(X(idx_range));

x_max = max(X(idx_range));

fprintf(' Fold %d: X ∈ [%.4f, %.4f] (%d samples)\n', fold, x_min, x_max, length(idx_range));

end

% Storage for spatial block CV results

quadratic_R2_spatial = zeros(K, 1);

quadratic_RMSE_spatial = zeros(K, 1);

lasso_R2_spatial = zeros(K, 1);

lasso_RMSE_spatial = zeros(K, 1);

rf_R2_spatial = zeros(K, 1);

rf_RMSE_spatial = zeros(K, 1);

nn_R2_spatial = zeros(K, 1);

nn_RMSE_spatial = zeros(K, 1);

best_X_quadratic_spatial = zeros(K, 1);

best_X_lasso_spatial = zeros(K, 1);

best_X_rf_spatial = zeros(K, 1);

best_X_nn_spatial = zeros(K, 1);

best_Y_quadratic_spatial = zeros(K, 1);

best_Y_lasso_spatial = zeros(K, 1);

best_Y_rf_spatial = zeros(K, 1);

best_Y_nn_spatial = zeros(K, 1);

for fold = 1:K

fprintf('\n--- Fold %d Spatial Block Cross-Validation ---\n', fold);

test_idx = (indices_spatial == fold);

train_idx = ~test_idx;

X_train = X(train_idx);

Y_train = Y(train_idx);

Z_train = Z(train_idx);

X_test = X(test_idx);

Y_test = Y(test_idx);

Z_test = Z(test_idx);

SS_tot = sum((Z_test - mean(Z_test)).^2);

% Calculate training set convex hull

train_unique = unique([X_train, Y_train], 'rows');

X_train_uniq = train_unique(:,1);

Y_train_uniq = train_unique(:,2);

if size(train_unique, 1) >= 3

DT_train = delaunayTriangulation(X_train_uniq, Y_train_uniq);

[K_train, ~] = convexHull(DT_train);

conv_hull_train = [X_train_uniq(K_train), Y_train_uniq(K_train)];

else

conv_hull_train = [min(X_train), min(Y_train); max(X_train), max(Y_train)];

end

% ======= Method 1: Quadratic Regression =======

A_train = [ones(size(X_train)), X_train, Y_train, ...

X_train.^2, Y_train.^2, X_train.*Y_train];

coefficients = A_train \ Z_train;

A_test = [ones(size(X_test)), X_test, Y_test, ...

X_test.^2, Y_test.^2, X_test.*Y_test];

Z_pred_quad = A_test * coefficients;

SS_res = sum((Z_test - Z_pred_quad).^2);

quadratic_R2_spatial(fold) = 1 - SS_res/SS_tot;

quadratic_RMSE_spatial(fold) = sqrt(mean((Z_test - Z_pred_quad).^2));

B0c = coefficients(1); B1c = coefficients(2); B2c = coefficients(3);

B3c = coefficients(4); B4c = coefficients(5); B5c = coefficients(6);

M = [2*B3c, B5c; B5c, 2*B4c];

RHS = [-B1c; -B2c];

if abs(det(M)) > 1e-10

XY_opt = M \ RHS;

best_X_quadratic_spatial(fold) = XY_opt(1);

best_Y_quadratic_spatial(fold) = XY_opt(2);

else

X_search_local = linspace(min(X_train), max(X_train), 100);

Y_search_local = linspace(min(Y_train), max(Y_train), 100);

max_Z_val = -inf;

for i = 1:length(X_search_local)

for j = 1:length(Y_search_local)

Z_val = B0c + B1c*X_search_local(i) + B2c*Y_search_local(j) + ...

B3c*X_search_local(i)^2 + B4c*Y_search_local(j)^2 + ...

B5c*X_search_local(i)*Y_search_local(j);

if Z_val > max_Z_val

max_Z_val = Z_val;

best_X_quadratic_spatial(fold) = X_search_local(i);

best_Y_quadratic_spatial(fold) = Y_search_local(j);

end

end

end

end

% ======= Method 2: Lasso Regression =======

A_train_lasso = [X_train, Y_train, X_train.^2, Y_train.^2, X_train.*Y_train];

A_test_lasso = [X_test, Y_test, X_test.^2, Y_test.^2, X_test.*Y_test];

[B, info] = lasso(A_train_lasso, Z_train, 'CV', 5);

idx = info.IndexMinMSE;

intercept = info.Intercept(idx);

coef = B(:, idx);

Z_pred_lasso = intercept + A_test_lasso * coef;

SS_res = sum((Z_test - Z_pred_lasso).^2);

lasso_R2_spatial(fold) = 1 - SS_res/SS_tot;

lasso_RMSE_spatial(fold) = sqrt(mean((Z_test - Z_pred_lasso).^2));

X_search_local = linspace(min(X_train), max(X_train), 100);

Y_search_local = linspace(min(Y_train), max(Y_train), 100);

max_Z_val = -inf;

for i = 1:length(X_search_local)

for j = 1:length(Y_search_local)

Z_val = intercept + coef(1)*X_search_local(i) + coef(2)*Y_search_local(j) + ...

coef(3)*X_search_local(i)^2 + coef(4)*Y_search_local(j)^2 + ...

coef(5)*X_search_local(i)*Y_search_local(j);

if Z_val > max_Z_val

max_Z_val = Z_val;

best_X_lasso_spatial(fold) = X_search_local(i);

best_Y_lasso_spatial(fold) = Y_search_local(j);

end

end

end

% ======= Method 3: Random Forest =======

rf_model = TreeBagger(100, [X_train, Y_train], Z_train, 'Method', 'regression');

Z_pred_rf = predict(rf_model, [X_test, Y_test]);

SS_res = sum((Z_test - Z_pred_rf).^2);

rf_R2_spatial(fold) = 1 - SS_res/SS_tot;

rf_RMSE_spatial(fold) = sqrt(mean((Z_test - Z_pred_rf).^2));

X_search_local = linspace(min(X_train), max(X_train), 100);

Y_search_local = linspace(min(Y_train), max(Y_train), 100);

max_Z_val = -inf;

for i = 1:length(X_search_local)

for j = 1:length(Y_search_local)

Z_val = predict(rf_model, [X_search_local(i), Y_search_local(j)]);

if Z_val > max_Z_val && ~isnan(Z_val)

max_Z_val = Z_val;

best_X_rf_spatial(fold) = X_search_local(i);

best_Y_rf_spatial(fold) = Y_search_local(j);

end

end

end

% ======= Method 4: Neural Network (using 3 hidden layer neurons) =======

X_min_train = min(X_train); X_max_train = max(X_train);

Y_min_train = min(Y_train); Y_max_train = max(Y_train);

Z_min_train = min(Z_train); Z_max_train = max(Z_train);

if X_max_train == X_min_train; X_max_train = X_min_train + eps; end

if Y_max_train == Y_min_train; Y_max_train = Y_min_train + eps; end

if Z_max_train == Z_min_train; Z_max_train = Z_min_train + eps; end

X_norm_train = (X_train - X_min_train) / (X_max_train - X_min_train);

Y_norm_train = (Y_train - Y_min_train) / (Y_max_train - Y_min_train);

Z_norm_train = (Z_train - Z_min_train) / (Z_max_train - Z_min_train);

rng(fold * 100 + 84);

net = feedforwardnet(3);

net.trainFcn = 'trainlm';

net.trainParam.epochs = 200;

net.trainParam.showWindow = false;

inputs_train = [X_norm_train'; Y_norm_train'];

targets_train = Z_norm_train';

[net_trained, ~] = train(net, inputs_train, targets_train);

X_norm_test = (X_test - X_min_train) / (X_max_train - X_min_train);

Y_norm_test = (Y_test - Y_min_train) / (Y_max_train - Y_min_train);

inputs_test = [X_norm_test'; Y_norm_test'];

Z_norm_pred = net_trained(inputs_test);

Z_pred_nn = Z_norm_pred' * (Z_max_train - Z_min_train) + Z_min_train;

SS_res = sum((Z_test - Z_pred_nn).^2);

nn_R2_spatial(fold) = 1 - SS_res/SS_tot;

nn_RMSE_spatial(fold) = sqrt(mean((Z_test - Z_pred_nn).^2));

X_search_local = linspace(min(X_train), max(X_train), 100);

Y_search_local = linspace(min(Y_train), max(Y_train), 100);

max_Z_val = -inf;

for i = 1:length(X_search_local)

for j = 1:length(Y_search_local)

if size(conv_hull_train, 1) >= 3

if ~inpolygon(X_search_local(i), Y_search_local(j), conv_hull_train(:,1), conv_hull_train(:,2))

continue;

end

end

X_norm_val = (X_search_local(i) - X_min_train) / (X_max_train - X_min_train);

Y_norm_val = (Y_search_local(j) - Y_min_train) / (Y_max_train - Y_min_train);

Z_pred_val = net_trained([X_norm_val; Y_norm_val]) * ...

(Z_max_train - Z_min_train) + Z_min_train;

if Z_pred_val > max_Z_val

max_Z_val = Z_pred_val;

best_X_nn_spatial(fold) = X_search_local(i);

best_Y_nn_spatial(fold) = Y_search_local(j);

end

end

end

fprintf('QR: R²=%.4f, RMSE=%.4f, Optimal X=%.4f\n', ...

quadratic_R2_spatial(fold), quadratic_RMSE_spatial(fold), best_X_quadratic_spatial(fold));

fprintf('Lasso: R²=%.4f, RMSE=%.4f, Optimal X=%.4f\n', ...

lasso_R2_spatial(fold), lasso_RMSE_spatial(fold), best_X_lasso_spatial(fold));

fprintf('RF: R²=%.4f, RMSE=%.4f, Optimal X=%.4f\n', ...

rf_R2_spatial(fold), rf_RMSE_spatial(fold), best_X_rf_spatial(fold));

fprintf('NN: R²=%.4f, RMSE=%.4f, Optimal X=%.4f\n', ...

nn_R2_spatial(fold), nn_RMSE_spatial(fold), best_X_nn_spatial(fold));

end

% ==================== 4. Statistical Analysis of Results ====================

fprintf('\n\n=== Statistical Analysis of Four Methods (Random CV) ===\n');

fprintf('\n1. Fitting Performance Comparison (5-fold CV):\n');

fprintf('--------------------------------------------------------------------\n');

fprintf('Method	Mean R²	R² Std	Mean RMSE	RMSE Std\n');

fprintf('--------------------------------------------------------------------\n');

fprintf('Quadratic	%.4f	%.4f	%.4f	%.4f\n', ...

mean(quadratic_R2), std(quadratic_R2), mean(quadratic_RMSE), std(quadratic_RMSE));

fprintf('Lasso	%.4f	%.4f	%.4f	%.4f\n', ...

mean(lasso_R2), std(lasso_R2), mean(lasso_RMSE), std(lasso_RMSE));

fprintf('Random Forest	%.4f	%.4f	%.4f	%.4f\n', ...

mean(rf_R2), std(rf_R2), mean(rf_RMSE), std(rf_RMSE));

fprintf('Neural Network	%.4f	%.4f	%.4f	%.4f\n', ...

mean(nn_R2), std(nn_R2), mean(nn_RMSE), std(nn_RMSE));

fprintf('--------------------------------------------------------------------\n');

fprintf('\n2. Optimal Solid Density Stability Comparison (Random CV):\n');

fprintf('Quadratic optimal X: %.4f ± %.4f (CV=%.2f%%)\n', mean(best_X_quadratic), std(best_X_quadratic), std(best_X_quadratic)/mean(best_X_quadratic)*100);

fprintf('Lasso optimal X: %.4f ± %.4f (CV=%.2f%%)\n', mean(best_X_lasso), std(best_X_lasso), std(best_X_lasso)/mean(best_X_lasso)*100);

fprintf('Random Forest optimal X: %.4f ± %.4f (CV=%.2f%%)\n', mean(best_X_rf), std(best_X_rf), std(best_X_rf)/mean(best_X_rf)*100);

fprintf('Neural Network optimal X: %.4f ± %.4f (CV=%.2f%%)\n', mean(best_X_nn), std(best_X_nn), std(best_X_nn)/mean(best_X_nn)*100);

% ==================== 4.5 Three CV Strategy Results Comparison ====================

fprintf('\n\n=== Comparison of Three Cross-Validation Strategies ===\n');

fprintf('Description:\n');

fprintf(' - Random CV: Randomly shuffled folds (baseline)\n');

fprintf(' - Temporal Block CV: Consecutive order folds (controls time dependence)\n');

fprintf(' - Spatial Block CV: X-value range folds (controls spatial dependence)\n');

fprintf('====================================================================\n');

fprintf('Model	| Metric | Random CV	| Temporal Block	| Spatial Block	\n');

fprintf('====================================================================\n');

print_compare3 = @(name, metric, val_random, val_block, val_spatial) ...

fprintf('%s	| %s	| %.4f ± %.4f | %.4f ± %.4f | %.4f ± %.4f\n', ...

name, metric, mean(val_random), std(val_random), mean(val_block), std(val_block), mean(val_spatial), std(val_spatial));

print_compare3('QR', 'R²', quadratic_R2, quadratic_R2_block, quadratic_R2_spatial);

print_compare3('Lasso', 'R²', lasso_R2, lasso_R2_block, lasso_R2_spatial);

print_compare3('RF', 'R²', rf_R2, rf_R2_block, rf_R2_spatial);

print_compare3('NN', 'R²', nn_R2, nn_R2_block, nn_R2_spatial);

print_compare3('QR', 'RMSE', quadratic_RMSE, quadratic_RMSE_block, quadratic_RMSE_spatial);

print_compare3('Lasso', 'RMSE', lasso_RMSE, lasso_RMSE_block, lasso_RMSE_spatial);

print_compare3('RF', 'RMSE', rf_RMSE, rf_RMSE_block, rf_RMSE_spatial);

print_compare3('NN', 'RMSE', nn_RMSE, nn_RMSE_block, nn_RMSE_spatial);

fprintf('====================================================================\n');

fprintf('\nModel ranking by strategy (by R²):\n');

fprintf(' Random CV: RF=%.4f, NN=%.4f, QR=%.4f, Lasso=%.4f\n', ...

mean(rf_R2), mean(nn_R2), mean(quadratic_R2), mean(lasso_R2));

fprintf(' Temporal CV: RF=%.4f, NN=%.4f, QR=%.4f, Lasso=%.4f\n', ...

mean(rf_R2_block), mean(nn_R2_block), mean(quadratic_R2_block), mean(lasso_R2_block));

fprintf(' Spatial CV: RF=%.4f, NN=%.4f, QR=%.4f, Lasso=%.4f\n', ...

mean(rf_R2_spatial), mean(nn_R2_spatial), mean(quadratic_R2_spatial), mean(lasso_R2_spatial));

% Generate bar chart comparing three CV strategies

figure('Position', [100, 100, 900, 600]);

bar_data = [mean(quadratic_R2), mean(lasso_R2), mean(rf_R2), mean(nn_R2);

mean(quadratic_R2_block), mean(lasso_R2_block), mean(rf_R2_block), mean(nn_R2_block);

mean(quadratic_R2_spatial), mean(lasso_R2_spatial), mean(rf_R2_spatial), mean(nn_R2_spatial)];

bar(bar_data);

set(gca, 'XTickLabel', {'QR', 'Lasso', 'RF', 'NN'});

legend('Random CV', 'Temporal Block CV', 'Spatial Block CV', 'Location', 'best');

ylabel('Mean R²');

title('Yellow Ink: R² Comparison Across Three CV Strategies');

grid on;

saveas(gcf, 'Yellow_Ink_CV_Comparison_Three.png');

fprintf('\nThree-strategy comparison figure saved: Yellow_Ink_CV_Comparison_Three.png\n');

% ==================== 5. Statistical Significance Test (Paired t-test) ====================

fprintf('\n=== Statistical Significance Test (Paired t-test) - Based on Random CV ===\n');

diff_R2_NN_QR = nn_R2 - quadratic_R2;

diff_R2_RF_QR = rf_R2 - quadratic_R2;

diff_R2_RF_NN = rf_R2 - nn_R2;

diff_R2_Lasso_QR = lasso_R2 - quadratic_R2;

[~, p_NN_QR] = ttest(diff_R2_NN_QR);

fprintf('\nNN vs QR:\n');

fprintf(' Mean R² difference: +%.4f\n', mean(diff_R2_NN_QR));

fprintf(' p = %.4f', p_NN_QR);

if p_NN_QR < 0.05

fprintf(' (Significant difference)\n');

else

fprintf(' (No significant difference)\n');

end

[~, p_RF_QR] = ttest(diff_R2_RF_QR);

fprintf('\nRF vs QR:\n');

fprintf(' Mean R² difference: +%.4f\n', mean(diff_R2_RF_QR));

fprintf(' p = %.4f', p_RF_QR);

if p_RF_QR < 0.05

fprintf(' (Significant difference)\n');

else

fprintf(' (No significant difference)\n');

end

[~, p_RF_NN] = ttest(diff_R2_RF_NN);

fprintf('\nRF vs NN:\n');

fprintf(' Mean R² difference: +%.4f\n', mean(diff_R2_RF_NN));

fprintf(' p = %.4f', p_RF_NN);

if p_RF_NN < 0.05

fprintf(' (Significant difference)\n');

else

fprintf(' (No significant difference)\n');

end

fprintf('\nLasso vs QR:\n');

fprintf(' Mean R² difference: +%.4f (No significant difference, p > 0.05)\n', mean(diff_R2_Lasso_QR));

% ==================== 6. Response Surface Analysis ====================

fprintf('\n=== Generating Response Surfaces ===\n');

% Grid points (yellow ink range)

xg = linspace(0.85, 1.15, 100);

yg = linspace(10, 20, 100);

[Xg, Yg] = meshgrid(xg, yg);

% Calculate actual data range

in_range = (Xg >= min(X)) & (Xg <= max(X)) & ...

(Yg >= min(Y)) & (Yg <= max(Y));

% QR response surface

Zg_qr = B0 + B1*Xg + B2*Yg + B3*Xg.^2 + B4*Yg.^2 + B5*Xg.*Yg;

Zg_qr(Zg_qr < 0) = NaN;

Zg_qr(~in_range) = NaN;

% Lasso response surface

Zg_lasso = intercept_lasso + coef_lasso(1)*Xg + coef_lasso(2)*Yg + ...

coef_lasso(3)*Xg.^2 + coef_lasso(4)*Yg.^2 + coef_lasso(5)*Xg.*Yg;

Zg_lasso(Zg_lasso < 0) = NaN;

Zg_lasso(~in_range) = NaN;

% RF response surface

Zg_rf = predict(rf_full, [Xg(:), Yg(:)]);

Zg_rf = reshape(Zg_rf, size(Xg));

Zg_rf(Zg_rf < 0) = NaN;

Zg_rf(~in_range) = NaN;

% NN response surface

Xg_norm = (Xg - X_min) / (X_max - X_min);

Yg_norm = (Yg - Y_min) / (Y_max - Y_min);

Zg_nn = net_full([Xg_norm(:)'; Yg_norm(:)'])';

Zg_nn = reshape(Zg_nn, size(Xg)) * (Z_max - Z_min) + Z_min;

Zg_nn(Zg_nn < 0) = NaN;

Zg_nn(~in_range) = NaN;

% Generate 2×2 comparison figure

figure('Position', [100, 100, 1200, 1000], 'Name', 'Yellow Ink: Four Model Response Surface Comparison');

subplot(2,2,1);

surf(Xg, Yg, Zg_qr, 'EdgeColor', 'none', 'FaceAlpha', 0.8);

hold on;

scatter3(X, Y, Z, 20, 'k', 'filled', 'MarkerFaceAlpha', 0.3);

title(sprintf('(a) Quadratic Regression QR (R²=%.4f)', R2_quad_full));

xlabel('Solid Density X'); ylabel('Dot Gain Y'); zlabel('Contrast Z');

colormap(jet); colorbar; view(45, 30); grid on;

zlim([min(Z), max(Z)]);

subplot(2,2,2);

surf(Xg, Yg, Zg_lasso, 'EdgeColor', 'none', 'FaceAlpha', 0.8);

hold on;

scatter3(X, Y, Z, 20, 'k', 'filled', 'MarkerFaceAlpha', 0.3);

title(sprintf('(b) Lasso Regression (R²=%.4f)', R2_lasso_full));

xlabel('Solid Density X'); ylabel('Dot Gain Y'); zlabel('Contrast Z');

colormap(jet); colorbar; view(45, 30); grid on;

zlim([min(Z), max(Z)]);

subplot(2,2,3);

surf(Xg, Yg, Zg_rf, 'EdgeColor', 'none', 'FaceAlpha', 0.8);

hold on;

scatter3(X, Y, Z, 20, 'k', 'filled', 'MarkerFaceAlpha', 0.3);

title(sprintf('(c) Random Forest RF (R²=%.4f)', R2_rf_full));

xlabel('Solid Density X'); ylabel('Dot Gain Y'); zlabel('Contrast Z');

colormap(jet); colorbar; view(45, 30); grid on;

zlim([min(Z), max(Z)]);

subplot(2,2,4);

surf(Xg, Yg, Zg_nn, 'EdgeColor', 'none', 'FaceAlpha', 0.8);

hold on;

scatter3(X, Y, Z, 20, 'k', 'filled', 'MarkerFaceAlpha', 0.3);

title(sprintf('(d) Neural Network MLP (Hidden=3, R²=%.4f)', R2_nn_full));

xlabel('Solid Density X'); ylabel('Dot Gain Y'); zlabel('Contrast Z');

colormap(jet); colorbar; view(45, 30); grid on;

zlim([min(Z), max(Z)]);

sgtitle('Yellow Ink: Four Model Response Surface Comparison');

saveas(gcf, 'Yellow_Ink_ResponseSurfaces.png');

fprintf(' Response surface figure saved: Yellow_Ink_ResponseSurfaces.png\n');

% ==================== 7. Cross-Validation Analysis ====================

fprintf('\n=== Optimal Point Cross-Validation Analysis ===\n');

% QR optimum predictions on each model

Z_nn_at_qr = interp2(Xg, Yg, Zg_nn, X_opt_quad, Y_opt_quad);

Z_rf_at_qr = interp2(Xg, Yg, Zg_rf, X_opt_quad, Y_opt_quad);

Z_lasso_at_qr = interp2(Xg, Yg, Zg_lasso, X_opt_quad, Y_opt_quad);

% NN optimum predictions on each model

Z_qr_at_nn = interp2(Xg, Yg, Zg_qr, best_X_nn_full, best_Y_nn_full);

Z_rf_at_nn = interp2(Xg, Yg, Zg_rf, best_X_nn_full, best_Y_nn_full);

Z_lasso_at_nn = interp2(Xg, Yg, Zg_lasso, best_X_nn_full, best_Y_nn_full);

% RF optimum predictions on each model

Z_qr_at_rf = interp2(Xg, Yg, Zg_qr, best_X_rf_full, best_Y_rf_full);

Z_nn_at_rf = interp2(Xg, Yg, Zg_nn, best_X_rf_full, best_Y_rf_full);

Z_lasso_at_rf = interp2(Xg, Yg, Zg_lasso, best_X_rf_full, best_Y_rf_full);

fprintf('\nQR optimum (X=%.4f, Y=%.4f, Z=%.4f):\n', X_opt_quad, Y_opt_quad, Z_opt_quad);

if ~isnan(Z_nn_at_qr); fprintf(' Prediction on NN: Z = %.4f (Diff: %+.4f)\n', Z_nn_at_qr, Z_nn_at_qr - Z_opt_quad);

else; fprintf(' Prediction on NN: Out of range\n'); end

if ~isnan(Z_rf_at_qr); fprintf(' Prediction on RF: Z = %.4f (Diff: %+.4f)\n', Z_rf_at_qr, Z_rf_at_qr - Z_opt_quad);

else; fprintf(' Prediction on RF: Out of range\n'); end

if ~isnan(Z_lasso_at_qr); fprintf(' Prediction on Lasso: Z = %.4f (Diff: %+.4f)\n', Z_lasso_at_qr, Z_lasso_at_qr - Z_opt_quad);

else; fprintf(' Prediction on Lasso: Out of range\n'); end

fprintf('\nNN optimum (X=%.4f, Y=%.4f, Z=%.4f):\n', best_X_nn_full, best_Y_nn_full, max_Z_nn);

if ~isnan(Z_qr_at_nn); fprintf(' Prediction on QR: Z = %.4f (Diff: %+.4f)\n', Z_qr_at_nn, Z_qr_at_nn - max_Z_nn);

else; fprintf(' Prediction on QR: Out of range\n'); end

if ~isnan(Z_rf_at_nn); fprintf(' Prediction on RF: Z = %.4f (Diff: %+.4f)\n', Z_rf_at_nn, Z_rf_at_nn - max_Z_nn);

else; fprintf(' Prediction on RF: Out of range\n'); end

if ~isnan(Z_lasso_at_nn); fprintf(' Prediction on Lasso: Z = %.4f (Diff: %+.4f)\n', Z_lasso_at_nn, Z_lasso_at_nn - max_Z_nn);

else; fprintf(' Prediction on Lasso: Out of range\n'); end

fprintf('\nRF optimum (X=%.4f, Y=%.4f, Z=%.4f):\n', best_X_rf_full, best_Y_rf_full, max_Z_rf);

if ~isnan(Z_qr_at_rf); fprintf(' Prediction on QR: Z = %.4f (Diff: %+.4f)\n', Z_qr_at_rf, Z_qr_at_rf - max_Z_rf);

else; fprintf(' Prediction on QR: Out of range\n'); end

if ~isnan(Z_nn_at_rf); fprintf(' Prediction on NN: Z = %.4f (Diff: %+.4f)\n', Z_nn_at_rf, Z_nn_at_rf - max_Z_rf);

else; fprintf(' Prediction on NN: Out of range\n'); end

if ~isnan(Z_lasso_at_rf); fprintf(' Prediction on Lasso: Z = %.4f (Diff: %+.4f)\n', Z_lasso_at_rf, Z_lasso_at_rf - max_Z_rf);

else; fprintf(' Prediction on Lasso: Out of range\n'); end

% ==================== 8. Residual Analysis ====================

fprintf('\n=== Residual Analysis ===\n');

residuals_qr = Z - Z_pred_quad_full;

residuals_lasso = Z - Z_pred_lasso_full;

residuals_rf = Z - Z_pred_rf_full;

residuals_nn = Z - Z_pred_nn_full;

figure('Position', [100, 100, 800, 800], 'Name', 'Yellow Ink Residual Analysis');

subplot(2,2,1);

scatter(Z_pred_quad_full, residuals_qr, 'b.');

hold on;

plot([min(Z_pred_quad_full), max(Z_pred_quad_full)], [0, 0], 'r--', 'LineWidth', 1);

xlabel('Predicted Value'); ylabel('Residual'); title('(a) QR Residual Plot');

grid on;

subplot(2,2,2);

scatter(Z_pred_lasso_full, residuals_lasso, 'g.');

hold on;

plot([min(Z_pred_lasso_full), max(Z_pred_lasso_full)], [0, 0], 'r--', 'LineWidth', 1);

xlabel('Predicted Value'); ylabel('Residual'); title('(b) Lasso Residual Plot');

grid on;

subplot(2,2,3);

scatter(Z_pred_rf_full, residuals_rf, 'r.');

hold on;

plot([min(Z_pred_rf_full), max(Z_pred_rf_full)], [0, 0], 'r--', 'LineWidth', 1);

xlabel('Predicted Value'); ylabel('Residual'); title('(c) RF Residual Plot');

grid on;

subplot(2,2,4);

scatter(Z_pred_nn_full, residuals_nn, 'm.');

hold on;

plot([min(Z_pred_nn_full), max(Z_pred_nn_full)], [0, 0], 'r--', 'LineWidth', 1);

xlabel('Predicted Value'); ylabel('Residual'); title('(d) NN Residual Plot');

grid on;

sgtitle('Yellow Ink: Four Model Residual Distribution Comparison');

saveas(gcf, 'Yellow_Ink_Residuals.png');

% ==================== 9. Generate Comparison Charts ====================

fprintf('\n=== Generating Comparison Charts ===\n');

% Figure 1: R² boxplot

figure('Position', [100, 100, 600, 500], 'Name', 'Yellow Ink R² Boxplot');

boxplot([quadratic_R2, lasso_R2, rf_R2, nn_R2], 'Labels', {'QR', 'Lasso', 'RF', 'NN'});

ylabel('Coefficient of Determination R²');

title('Yellow Ink: R² Distribution Comparison (Random 5-fold CV)');

grid on;

saveas(gcf, 'Yellow_Ink_R2_Boxplot.png');

% Figure 2: RMSE boxplot

figure('Position', [150, 150, 600, 500], 'Name', 'Yellow Ink RMSE Boxplot');

boxplot([quadratic_RMSE, lasso_RMSE, rf_RMSE, nn_RMSE], 'Labels', {'QR', 'Lasso', 'RF', 'NN'});

ylabel('Root Mean Square Error RMSE');

title('Yellow Ink: RMSE Distribution Comparison (Random 5-fold CV)');

grid on;

saveas(gcf, 'Yellow_Ink_RMSE_Boxplot.png');

% Figure 3: Optimal X stability line chart (Random CV)

figure('Position', [200, 200, 700, 500], 'Name', 'Yellow Ink Optimal X Stability');

hold on;

plot(1:K, best_X_quadratic, 'bo-', 'LineWidth', 2, 'MarkerSize', 8, 'DisplayName', 'QR');

plot(1:K, best_X_lasso, 'go-', 'LineWidth', 2, 'MarkerSize', 8, 'DisplayName', 'Lasso');

plot(1:K, best_X_rf, 'ro-', 'LineWidth', 2, 'MarkerSize', 8, 'DisplayName', 'RF');

plot(1:K, best_X_nn, 'mo-', 'LineWidth', 2, 'MarkerSize', 8, 'DisplayName', 'NN');

xlabel('Cross-Validation Fold'); ylabel('Optimal Solid Density X');

title('Yellow Ink: Optimal Solid Density Variation Across Folds (Random CV)');

legend('Location', 'best'); grid on;

xlim([0.5, 5.5]);

saveas(gcf, 'Yellow_Ink_Xopt_Stability.png');

% Figure 4: Mean performance bar chart

figure('Position', [250, 250, 800, 500], 'Name', 'Yellow Ink Mean Performance Comparison');

subplot(1,2,1);

mean_R2 = [mean(quadratic_R2), mean(lasso_R2), mean(rf_R2), mean(nn_R2)];

std_R2 = [std(quadratic_R2), std(lasso_R2), std(rf_R2), std(nn_R2)];

bar(1:4, mean_R2, 0.6, 'FaceColor', [0.5 0.5 0.8]);

hold on;

errorbar(1:4, mean_R2, std_R2, 'k.', 'LineWidth', 1.5, 'MarkerSize', 15);

set(gca, 'XTickLabel', {'QR', 'Lasso', 'RF', 'NN'});

ylabel('Mean R²'); title('Yellow Ink: Mean R² Comparison');

ylim([0.7, 1.0]); grid on;

subplot(1,2,2);

mean_RMSE = [mean(quadratic_RMSE), mean(lasso_RMSE), mean(rf_RMSE), mean(nn_RMSE)];

std_RMSE = [std(quadratic_RMSE), std(lasso_RMSE), std(rf_RMSE), std(nn_RMSE)];

bar(1:4, mean_RMSE, 0.6, 'FaceColor', [0.8 0.5 0.5]);

hold on;

errorbar(1:4, mean_RMSE, std_RMSE, 'k.', 'LineWidth', 1.5, 'MarkerSize', 15);

set(gca, 'XTickLabel', {'QR', 'Lasso', 'RF', 'NN'});

ylabel('Mean RMSE'); title('Yellow Ink: Mean RMSE Comparison');

grid on;

sgtitle('Yellow Ink: Four Model Mean Performance Comparison (Random 5-fold CV)');

saveas(gcf, 'Yellow_Ink_Performance_Bar.png');

fprintf('\n=== Yellow Ink Analysis Complete! ===\n');

fprintf('MLP hidden layer neuron count: 3 (optimal result from hyperparameter search)\n');

fprintf('Generated image files:\n');

fprintf(' Yellow_Ink_Autocorrelation.png\n');

fprintf(' Yellow_Ink_CV_Comparison_Three.png <- New: Three CV strategy comparison\n');

fprintf(' Yellow_Ink_ResponseSurfaces.png\n');

fprintf(' Yellow_Ink_Residuals.png\n');

fprintf(' Yellow_Ink_R2_Boxplot.png\n');

fprintf(' Yellow_Ink_RMSE_Boxplot.png\n');

fprintf(' Yellow_Ink_Xopt_Stability.png\n');

fprintf(' Yellow_Ink_Performance_Bar.png\n');
