# Supplementary material for: Comparative study of quadratic regression, Lasso, random forest, and multilayer perceptron for modeling optimal solid density in offset printing
Source: PLoS One. 2026 Oct 21;21(10):e0361203. doi: 10.1371/journal.pone.0361203 (PMC13686465; doi:10.1371/journal.pone.0361203)
Supplement: Supplementary file 1 [file pone.0361203.s001.zip › Supporting Information/S1 Table .docx]

**Table S1. Comprehensive statistics of black ink model performance and parameter robustness**

| **Category** | **Metric** | **QR** | **Lasso** | **RF** | **MLP** |
| --- | --- | --- | --- | --- | --- |
| **Prediction Performance (5-fold CV)** | Mean R² | 0.8900 | 0.8873 | **0.9628** | 0.9588 |
|  | SD of R² | 0.0431 | 0.0488 | 0.0223 | 0.0210 |
|  | Mean RMSE | 1.6658 | 1.6794 | **0.9339** | 1.0012 |
|  | SD of RMSE | 0.2261 | 0.2732 | 0.2355 | 0.1899 |
| **Optimal Parameters (Full data)** | Optimal X | 1.7403 | 1.7747 | **1.6734** | 1.7544 |
|  | Optimal Y | 18.5649 | 18.3544 | **19.1139** | 18.0380 |
|  | Predicted Max Z | 41.4549 | 41.5651 | **43.5303** | 43.0389 |
| **Parameter Robustness (5-fold CV)** | Optimal X (Mean±Std) | 1.7606±0.0564 | 1.7916±0.0495 | **1.6721±0.0046** | 1.7401±0.0981 |
|  | CV% of X | 3.20% | 2.76% | **0.28%** | 5.64% |
|  | Relative Deviation | 1.16% | 0.95% | **0.08%** | 0.82% |
| **Consistency Check** | QR Optimum on RF (Z') | — | — | 42.09 | — |
|  | QR Optimum on MLP (Z') | — | — | — | 42.89 |
|  | RF Optimum on QR (Z') | 40.66 | — | — | — |
|  | RF Optimum on MLP (Z') | — | — | — | 42.44 |
|  | MLP Optimum on QR (Z') | 41.12 | — | — | — |
|  | MLP Optimum on RF (Z') | — | — | 41.77 | — |

**Note:** Bold values indicate the best performance among the four models.

**Paired t-test results for R² differences (5-fold CV):**

| **Comparison** | **Mean R² Difference** | **p-value** | **Significance** |
| --- | --- | --- | --- |
| RF vs QR | +0.0728 | **0.0035** | Significant |
| MLP vs QR | +0.0688 | **0.0124** | Significant |
| RF vs MLP | +0.0040 | 0.5838 | Not significant |
| Lasso vs QR | -0.0027 | >0.05 | Not significant |

**Key findings for black ink:**

**1. Prediction accuracy:** RF achieved the highest mean R² (0.9628) and lowest RMSE (0.9339), outperforming all other models.

**2. Parameter stability:** RF exhibited the best stability with CV of only 0.28% for optimal solid density, compared to QR (3.20%), Lasso (2.76%), and MLP (5.64%).

**3. Statistical significance:** Both RF and MLP significantly outperformed QR (p < 0.05), while no significant difference was found between RF and MLP (p = 0.5838).

**4. Consistency:** The RF optimal point produced consistent predictions when evaluated on other models (42.09 on MLP, 40.66 on QR), confirming the robustness of the broad plateau characteristic.

**5. Recommended model:** RF is recommended for black ink with an optimal solid density of **1.67**, achieving a target relative contrast of ≥ 42.0.
