# Supplementary material for: Comparative study of quadratic regression, Lasso, random forest, and multilayer perceptron for modeling optimal solid density in offset printing
Source: PLoS One. 2026 Oct 21;21(10):e0361203. doi: 10.1371/journal.pone.0361203 (PMC13686465; doi:10.1371/journal.pone.0361203)
Supplement: Supplementary file 1 [file pone.0361203.s001.zip › Supporting Information/S1 Text .docx]

**Modeling and Analysis for Optimal Solid Density Optimization in Offset Printing**

**Authors:** Bingzhong QIU ^1^, Liangfei LI^2, 4^, Xiaoli LIU ^1*^, Xiaolei SHENG^1^, Yujun JIANG ^1^, Xiaomei LIU ^1^,Ying-Leh Ling^3^

**Affiliation:**

^1^ General Education Department, Qilu Medical University, Shandong, China

^2^ College of Vocational Education, Tianjin University of Technology and Education，Tianjin，China

^3^ Department of Mathematics, Science and Computer, Politeknik Kuching Sarawak, Sarawak, Malaysia

^4^ Academic Affairs Office, Zibo Polytechnical Vocational School, Shandong, China

**Keywords:** Offset printing; Solid density; Quadratic regression; Lasso; Random forest; Multilayer perceptron; Response surface; 5-fold cross-validation

**1. Introduction**

This protocol describes a systematic method for optimizing the optimal solid density of four-color offset printing inks. The core of this method involves first collecting solid density, dot gain, and relative contrast data through controlled printing experiments, then constructing quadratic regression (QR), Lasso, random forest (RF), and multilayer perceptron (MLP) prediction models respectively, and systematically comparing the four modeling approaches using 5-fold cross-validation and a consistency check mechanism, ultimately determining the optimal solid density and its robust control range for each ink color. This protocol is an integral part of the submitted research paper "Comparative Study of Quadratic Regression, Lasso, Random Forest, and Multilayer Perceptron for Modeling Optimal Solid Density in Offset Printing", providing complete, reproducible step-by-step procedures for the operations described in the "Methods" section (Section 2) of the paper, ensuring research transparency and reproducibility.

**2. Experimental Materials**

**2.1 Software**

- CorelDraw 2019 (for test form design)
- MATLAB R2016a (MathWorks, USA) (for data processing and modeling)
- SPSS 26.0 (for data preprocessing and outlier screening)

**2.2 Consumables**

- **Inks:** Tianjin Toyo TGO-NO four-color offset inks (Cyan C, Magenta M, Yellow Y, Black K)
- **Paper:** 157 g/m² double-sided coated art paper

**2.3 Equipment**

- **Printing press:** Jiangsu Zhongjing PZ4740E-AL four-color offset press (or equivalent model with precise calibration)

Configuration: 175 LPI round dots

Color sequence: Black → Magenta → Cyan → Yellow

Printing pressure: 0.15 mm

Printing speed: 8,000 sheets/hour

- **Spectrophotometer:** X-Rite eXact (or equivalent model), calibrated to D50 illuminant and 2° standard observer, density repeatability ±0.01
- **Environmental control:** Temperature 23±1°C, relative humidity 60±5% RH

**3. Experimental Procedure**

**3.1 Environmental Preparation**

The experiment shall be conducted under constant environmental conditions: temperature 23±1°C, relative humidity 60±5% RH.

**3.2 Design and Print the Test Form**

**3.2.1 Design the Test Form**

Create the test form file using CorelDraw software according to the layout shown in Figure 1 of the main paper.

- **Gradient areas**: Design four sets of gradients for cyan, magenta, yellow, and black inks. Each gradient should have dot areas ranging from 0% to 100% in 5% increments (i.e., 5%, 10%, 15%, …, 100%). Use round dot shape.
- **Quality control**: Ensure sufficient blank areas are included in the test form for background measurement and quality control marks.

**3.2.2 Output the Plates**

Output the digital file to printing plates at a screen frequency of 175 LPI.

**3.2.3 Print the Test Form**

Print the test forms on the offset press using 157 g/m² coated paper.

- **Printing sequence**: Black (K) → Magenta (M) → Cyan (C) → Yellow (Y)
- Before printing formal samples, ensure the press is properly calibrated and ink-water balance is achieved.
- Extract 120 initial printed sheets from continuous production runs. After visually inspecting and eliminating samples with defects such as ghosting, ink streaks, or dirt spots, obtain 110 valid samples for measurement.

**3.3 Parameter Measurement**

**3.3.1 Calibrate the Spectrophotometer**

Power on the X-Rite eXact spectrophotometer and perform zero calibration according to the manufacturer's instructions.

**3.3.2 Measurement Parameters**

- Using the calibrated X-Rite eXact spectrophotometer, measure the following three parameters for each of the four colors on every valid sample:
  - **Solid density (X)**: Measure on the solid patch of each color. The instrument directly displays the solid density value.
  - **Dot gain (Y)**: Measure on the 50% dot patch of each color. The instrument automatically calculates and directly displays the dot gain value based on the Murray-Davies formula.
  - **Relative contrast (Z)**: Measure on both the solid patch and the 75% dot patch of each color. The instrument automatically calculates and directly displays the relative contrast value based on its built-in formula.

Each parameter shall be measured three times at the same position, and the arithmetic mean shall be taken as the final value to minimize measurement error.

- **Quality control**: Set the threshold to ±0.02 (based on instrument repeatability of ±0.01 and industry practices). If the standard deviation of the three readings exceeds this threshold, the data shall be considered abnormal and the measurement shall be repeated.

**3.3.3 Data Recording**

A total of 440 data points (4 colors × 110 samples) are obtained. The data structure should be organized as follows: for each color, a 110 × 3 data matrix is created, with columns corresponding to solid density (X), dot gain (Y), and relative contrast (Z), respectively.

**4. Data Analysis and Modeling in MATLAB**

**4.1 Data Organization in MATLAB**

Create vectors in MATLAB for each ink color to store solid density (X), dot gain (Y), and relative contrast (Z), respectively.

Using black ink as an example (the complete dataset is available in Supporting Information S1 Data):

*% Black ink data (partial example)*

X_black = [1.33, 1.35, 1.37, 1.36, 1.38, 1.38, 1.39, 1.40, 1.41, 1.41]; *% Solid density*

Y_black = [15.81, 15.85, 15.63, 15.42, 15.41, 15.52, 15.51, 15.78, 15.75, 15.66]; *% Dot gain*

Z_black = [27.8, 27.6, 27.7, 27.8, 27.5, 27.2, 27.1, 26.8, 26.7, 26.7]; *% Relative contrast*

**4.2 MLP Hyperparameter Search**

Before constructing the final MLP model, a systematic hyperparameter search shall be conducted to determine the optimal number of hidden neurons for each ink color:

*% Hyperparameter search for MLP*

hidden_sizes = [3, 4, 5, 6, 8, 10];

cv_K = 5;

rng(42);

indices_temp = crossvalind('Kfold', n, cv_K);

search_results = zeros(length(hidden_sizes), 2);

for h_idx = 1:length(hidden_sizes)

h = hidden_sizes(h_idx);

rmse_folds = zeros(cv_K, 1);

for fold = 1:cv_K

test_idx = (indices_temp == fold);

train_idx = ~test_idx;

X_train = X(train_idx);

Y_train = Y(train_idx);

Z_train = Z(train_idx);

X_test = X(test_idx);

Y_test = Y(test_idx);

Z_test = Z(test_idx);

*% Normalize*

X_min_tr = min(X_train); X_max_tr = max(X_train);

Y_min_tr = min(Y_train); Y_max_tr = max(Y_train);

Z_min_tr = min(Z_train); Z_max_tr = max(Z_train);

X_norm_train = (X_train - X_min_tr) / (X_max_tr - X_min_tr);

Y_norm_train = (Y_train - Y_min_tr) / (Y_max_tr - Y_min_tr);

Z_norm_train = (Z_train - Z_min_tr) / (Z_max_tr - Z_min_tr);

X_norm_test = (X_test - X_min_tr) / (X_max_tr - X_min_tr);

Y_norm_test = (Y_test - Y_min_tr) / (Y_max_tr - Y_min_tr);

rng(42);

net = feedforwardnet(h);

net.trainFcn = 'trainlm';

net.trainParam.epochs = 200;

net.trainParam.showWindow = false;

net.divideFcn = 'dividetrain';

net = train(net, [X_norm_train'; Y_norm_train'], Z_norm_train');

Z_norm_pred = net([X_norm_test'; Y_norm_test']);

Z_pred = Z_norm_pred' * (Z_max_tr - Z_min_tr) + Z_min_tr;

rmse_folds(fold) = sqrt(mean((Z_test - Z_pred).^2));

end

search_results(h_idx, 1) = mean(rmse_folds);

search_results(h_idx, 2) = std(rmse_folds);

fprintf('Hidden neurons=%d: RMSE=%.4f ± %.4f\n', h, search_results(h_idx, 1), search_results(h_idx, 2));

end

[~, best_idx] = min(search_results(:, 1));

best_hidden = hidden_sizes(best_idx);

fprintf('Optimal hidden neurons: %d\n', best_hidden);

**Note:** Based on hyperparameter search results:

- Black, cyan, magenta inks: 5 neurons (within 10% of optimum)
- Yellow ink: 3 neurons (5-neuron exhibited training instability)

**4.3 Core Code for 5-Fold Cross-Validation**

*% Generate 5-fold cross-validation indices*

n = length(X);

K = 5;

rng(42);

indices = crossvalind('Kfold', n, K); *% Requires Statistics and Machine Learning Toolbox*

*% Initialize storage variables for all four models*

quadratic_R2 = zeros(K, 1);

lasso_R2 = zeros(K, 1);

rf_R2 = zeros(K, 1);

mlp_R2 = zeros(K, 1);

best_X_quadratic = zeros(K, 1);

best_X_lasso = zeros(K, 1);

best_X_rf = zeros(K, 1);

best_X_mlp = zeros(K, 1);

*% Perform 5-fold cross-validation*

for fold = 1:K

*% Split training and test sets*

test_idx = (indices == fold);

train_idx = ~test_idx;

X_train = X(train_idx);

Y_train = Y(train_idx);

Z_train = Z(train_idx);

X_test = X(test_idx);

Y_test = Y(test_idx);

Z_test = Z(test_idx);

SS_tot = sum((Z_test - mean(Z_test)).^2);

*% ===== Quadratic Regression Model =====*

A_train = [ones(size(X_train)), X_train, Y_train, X_train.^2, Y_train.^2, X_train.*Y_train];

coeff = A_train \ Z_train;

A_test = [ones(size(X_test)), X_test, Y_test, X_test.^2, Y_test.^2, X_test.*Y_test];

Z_pred_quad = A_test * coeff;

SS_res = sum((Z_test - Z_pred_quad).^2);

quadratic_R2(fold) = 1 - SS_res/SS_tot;

*% ===== Lasso Regression Model =====*

A_train_lasso = [X_train, Y_train, X_train.^2, Y_train.^2, X_train.*Y_train];

[B, info] = lasso(A_train_lasso, Z_train, 'CV', 5);

idx = info.IndexMinMSE;

intercept = info.Intercept(idx);

coef = B(:, idx);

Z_pred_lasso = intercept + A_test * coef;

SS_res = sum((Z_test - Z_pred_lasso).^2);

lasso_R2(fold) = 1 - SS_res/SS_tot;

*% ===== Random Forest Model =====*

rf_model = TreeBagger(50, [X_train, Y_train], Z_train, 'Method', 'regression');

Z_pred_rf = predict(rf_model, [X_test, Y_test]);

SS_res = sum((Z_test - Z_pred_rf).^2);

rf_R2(fold) = 1 - SS_res/SS_tot;

*% ===== Multilayer Perceptron Model =====*

X_min_train = min(X_train); X_max_train = max(X_train);

Y_min_train = min(Y_train); Y_max_train = max(Y_train);

Z_min_train = min(Z_train); Z_max_train = max(Z_train);

X_norm_train = (X_train - X_min_train) / (X_max_train - X_min_train);

Y_norm_train = (Y_train - Y_min_train) / (Y_max_train - Y_min_train);

Z_norm_train = (Z_train - Z_min_train) / (Z_max_train - Z_min_train);

rng(fold * 100);

net = feedforwardnet(5); % Use 5 for black/cyan/magenta, 3 for yellow

net.trainFcn = 'trainlm';

net.trainParam.epochs = 200;

net.trainParam.showWindow = false;

inputs_train = [X_norm_train'; Y_norm_train'];

targets_train = Z_norm_train';

net = train(net, inputs_train, targets_train);

X_norm_test = (X_test - X_min_train) / (X_max_train - X_min_train);

Y_norm_test = (Y_test - Y_min_train) / (Y_max_train - Y_min_train);

Z_pred_norm = net([X_norm_test'; Y_norm_test']);

Z_pred_mlp = Z_pred_norm' * (Z_max_train - Z_min_train) + Z_min_train;

SS_res = sum((Z_test - Z_pred_mlp).^2);

mlp_R2(fold) = 1 - SS_res/SS_tot;

*% Optimal point search using 100×100 grid (for CV% calculation)*

X_search = linspace(min(X_train), max(X_train), 100);

Y_search = linspace(min(Y_train), max(Y_train), 100);

% Find optimal X for each model (code omitted for brevity)

end

*% Output results*

fprintf('QR mean R²: %.4f ± %.4f\n', mean(quadratic_R2), std(quadratic_R2));

fprintf('Lasso mean R²: %.4f ± %.4f\n', mean(lasso_R2), std(lasso_R2));

fprintf('RF mean R²: %.4f ± %.4f\n', mean(rf_R2), std(rf_R2));

fprintf('MLP mean R²: %.4f ± %.4f\n', mean(mlp_R2), std(mlp_R2));

**4.4 Consistency Check (Mutual Validation)**

*% Train full-data models*

*% Quadratic regression*

A_full = [ones(size(X)), X, Y, X.^2, Y.^2, X.*Y];

coeff_full = A_full \ Z;

B0 = coeff_full(1); B1 = coeff_full(2); B2 = coeff_full(3);

B3 = coeff_full(4); B4 = coeff_full(5); B5 = coeff_full(6);

*% Lasso regression*

A_lasso_full = [X, Y, X.^2, Y.^2, X.*Y];

[B_lasso, info_lasso] = lasso(A_lasso_full, Z, 'CV', 5);

idx_lasso = info_lasso.IndexMinMSE;

intercept_lasso = info_lasso.Intercept(idx_lasso);

coef_lasso = B_lasso(:, idx_lasso);

*% Random forest*

rf_full = TreeBagger(100, [X, Y], Z, 'Method', 'regression');

*% MLP*

X_min = min(X); X_max = max(X);

Y_min = min(Y); Y_max = max(Y);

Z_min = min(Z); Z_max = max(Z);

X_norm = (X - X_min) / (X_max - X_min);

Y_norm = (Y - Y_min) / (Y_max - Y_min);

Z_norm = (Z - Z_min) / (Z_max - Z_min);

rng(42);

mlp_full = feedforwardnet(5); *% Use 5 for black/cyan/magenta, 3 for yellow*

mlp_full.trainFcn = 'trainlm';

mlp_full.trainParam.epochs = 300;

mlp_full.trainParam.showWindow = false;

mlp_full = train(mlp_full, [X_norm'; Y_norm'], Z_norm');

*% Grid search for optimal points (100×100 grid)*

X_search = linspace(min(X), max(X), 100);

Y_search = linspace(min(Y), max(Y), 100);

*% Find optimal points for each model (code omitted for brevity)*

*% Consistency check: predict optimal points across models*

*% Example for QR optimal point in MLP*

X_opt_quad = 1.7403; Y_opt_quad = 18.5649; *% QR optimal point*

X_norm_quad = (X_opt_quad - X_min) / (X_max - X_min);

Y_norm_quad = (Y_opt_quad - Y_min) / (Y_max - Y_min);

Z_mlp_at_quad = mlp_full([X_norm_quad; Y_norm_quad]) * (Z_max - Z_min) + Z_min;

fprintf('Consistency: QR optimal point predicted by MLP = %.4f\n', Z_mlp_at_quad);

**4.5 Response Surface Visualization**

***% Construct grid with 100×100 resolution***

**[X_mesh, Y_mesh] = meshgrid(linspace(min(X), max(X), 100), linspace(min(Y), max(Y), 100));**

***% Restrict to convex hull of training data***

**DT = delaunayTriangulation(X, Y);**

**[K, ~] = convexHull(DT);**

**conv_hull_vertices = [X(K), Y(K)];**

**in_hull = inpolygon(X_mesh, Y_mesh, conv_hull_vertices(:,1), conv_hull_vertices(:,2));**

***% Calculate quadratic regression surface***

**Z_quad = B0 + B1*X_mesh + B2*Y_mesh + B3*X_mesh.^2 + B4*Y_mesh.^2 + B5*X_mesh.*Y_mesh;**

**Z_quad(~in_hull) = NaN;**

***% Calculate Lasso surface***

**Z_lasso = intercept_lasso + coef_lasso(1)*X_mesh + coef_lasso(2)*Y_mesh + ...**

**coef_lasso(3)*X_mesh.^2 + coef_lasso(4)*Y_mesh.^2 + coef_lasso(5)*X_mesh.*Y_mesh;**

**Z_lasso(~in_hull) = NaN;**

***% Calculate random forest surface***

**Z_rf = reshape(predict(rf_full, [X_mesh(:), Y_mesh(:)]), size(X_mesh));**

**Z_rf(~in_hull) = NaN;**

***% Calculate MLP surface***

**X_mesh_norm = (X_mesh - X_min) / (X_max - X_min);**

**Y_mesh_norm = (Y_mesh - Y_min) / (Y_max - Y_min);**

**Z_mlp = mlp_full([X_mesh_norm(:)'; Y_mesh_norm(:)'])';**

**Z_mlp = reshape(Z_mlp, size(X_mesh)) * (Z_max - Z_min) + Z_min;**

**Z_mlp(~in_hull) = NaN;**

***% Plot 2×2 comparison figure***

**figure('Position', [100, 100, 1200, 1000]);**

**subplot(2,2,1);**

**surf(X_mesh, Y_mesh, Z_quad, 'EdgeColor', 'none', 'FaceAlpha', 0.8);**

**hold on;**

**scatter3(X, Y, Z, 20, 'k', 'filled');**

**title('(a) Quadratic Regression (QR)');**

**xlabel('Solid Density X'); ylabel('Dot Gain Y'); zlabel('Relative Contrast Z');**

**colormap(jet); colorbar; view(45, 30); grid on;**

**subplot(2,2,2);**

**surf(X_mesh, Y_mesh, Z_lasso, 'EdgeColor', 'none', 'FaceAlpha', 0.8);**

**hold on;**

**scatter3(X, Y, Z, 20, 'k', 'filled');**

**title('(b) Lasso Regression');**

**xlabel('Solid Density X'); ylabel('Dot Gain Y'); zlabel('Relative Contrast Z');**

**colormap(jet); colorbar; view(45, 30); grid on;**

**subplot(2,2,3);**

**surf(X_mesh, Y_mesh, Z_rf, 'EdgeColor', 'none', 'FaceAlpha', 0.8);**

**hold on;**

**scatter3(X, Y, Z, 20, 'k', 'filled');**

**title('(c) Random Forest (RF)');**

**xlabel('Solid Density X'); ylabel('Dot Gain Y'); zlabel('Relative Contrast Z');**

**colormap(jet); colorbar; view(45, 30); grid on;**

**subplot(2,2,4);**

**surf(X_mesh, Y_mesh, Z_mlp, 'EdgeColor', 'none', 'FaceAlpha', 0.8);**

**hold on;**

**scatter3(X, Y, Z, 20, 'k', 'filled');**

**title('(d) Multilayer Perceptron (MLP)');**

**xlabel('Solid Density X'); ylabel('Dot Gain Y'); zlabel('Relative Contrast Z');**

**colormap(jet); colorbar; view(45, 30); grid on;**

**sgtitle('Response Surface Comparison of Four Models');**

**saveas(gcf, 'ResponseSurfaces.png');**

**4.6 Robust Control Range Calculation**

*% Calculate control range from cross-validation results*

mu_X = mean(best_X_mlp);

sigma_X = std(best_X_mlp);

fprintf('Statistical control range (μ ± 2σ): [%.4f, %.4f]\n', mu_X - 2*sigma_X, mu_X + 2*sigma_X);

**4.7 Statistical Significance Test (Paired t-test)**

*% Perform paired t-test between model pairs*

diff_R2_MLP_QR = mlp_R2 - quadratic_R2;

[~, p_MLP_QR] = ttest(diff_R2_MLP_QR);

fprintf('MLP vs QR: p = %.4f\n', p_MLP_QR);

diff_R2_RF_QR = rf_R2 - quadratic_R2;

[~, p_RF_QR] = ttest(diff_R2_RF_QR);

fprintf('RF vs QR: p = %.4f\n', p_RF_QR);

diff_R2_RF_MLP = rf_R2 - mlp_R2;

[~, p_RF_MLP] = ttest(diff_R2_RF_MLP);

fprintf('RF vs MLP: p = %.4f\n', p_RF_MLP);

**5. Expected Results**

5.1 Successful printing of test forms containing four-color scales.

5.2 Acquisition of 440 reliable measurement data points for solid density, dot gain, and relative contrast.

5.3 Successful construction of quadratic regression, Lasso, random forest, and MLP prediction models for each ink color.

5.4 Determination of optimal MLP architecture via hyperparameter search:

Black, cyan, magenta inks: 5 neurons

Yellow ink: 3 neurons

5.5 Obtainment of performance metrics (R², RMSE, CV%) for all four models under 5-fold cross-validation, along with paired t-test results.

5.6 Generation of response surface plots for all four models for each ink color.

5.7 Determination of recommended optimal solid density values:

Black: 1.67 (RF)

Cyan: 1.44 (RF)

Magenta: 1.42 (RF)

Yellow: 0.99 (MLP with 3 neurons)

5.8 Validation results demonstrating that non-parametric methods (RF and MLP) significantly outperform parametric methods (QR and Lasso) in prediction accuracy, with RF recommended for black, cyan, and magenta inks, and MLP recommended for yellow ink.

**6.** **Troubleshooting**

| **Problem** | **Possible Cause** | **Solution** |
| --- | --- | --- |
| Poor model fit (low R²) | Data noise or outliers | Check calibration; review and clean data; ensure stable printing conditions |
| MLP overfitting | Too many neurons or insufficient data | Reduce neurons; increase regularization; use early stopping |
| Analytical solution failure | Singular matrix or solution out of range | Use grid search instead; verify parameter range |
| Unstable CV results | Non-random partitioning or model instability | Use random partitioning; fix random seeds; check fold distribution |
| Large consistency check deviation | Systematic model differences | Expected phenomenon; analyze with response surface characteristics |
| Grid search misses optimum | Wrong range or step size | Adjust range and step; use coarse-to-fine search |
| MATLAB errors | Syntax or dimension mismatch | Check code; ensure consistent vector lengths and matrix dimensions |
| Yellow MLP training instability | 5-neuron configuration | Use 3-neuron MLP based on hyperparameter search |

**7. Linkage to the Main Text**

This supporting information protocol is directly linked to the following sections of the main paper:

(1) Section 3 (Experimental Procedure) of this protocol corresponds to the "Experimental Design" and "Data Collection" sections of the main paper, providing detailed operational procedures for experimental equipment, materials, environmental control, and data acquisition.

(2) Section 4.2 (MLP Hyperparameter Search) of this protocol corresponds to the "Multilayer Perceptron" section of the main paper, providing the hyperparameter search implementation that determines the optimal number of hidden neurons for each ink color.

(3) Section 4.3 (Core Code for 5-Fold Cross-Validation) of this protocol corresponds to the "Predictive Model Construction" and "Model Validation and Robustness Assessment" sections of the main paper, offering MATLAB code implementations for all four modeling approaches, 5-fold cross-validation, and the consistency check mechanism.

(4) Section 4.4 (Consistency Check) of this protocol corresponds to the "consistency check" mechanism proposed in the "Model Validation and Robustness Assessment" section of the main paper.

(5) Section 4.5 (Response Surface Visualization) of this protocol corresponds to the "Surface Reconstruction and Visualization" section of the main paper, providing complete code for generating response surfaces with convex hull restrictions.

(6) Section 4.6 (Robust Control Range Calculation) of this protocol corresponds to the "Robust Control Range Calculation" section of the main paper.

(7) Section 4.7 (Statistical Significance Test) of this protocol corresponds to the statistical analysis section of the main paper.

All R², RMSE, CV%, optimal solid density values, control ranges, p-values, and final conclusions presented in the main paper are strictly derived from the experimental procedures and MATLAB code described in this protocol.

**References**

References are identical to those cited in the main article.
