# Supplementary material for: Comparative study of quadratic regression, Lasso, random forest, and multilayer perceptron for modeling optimal solid density in offset printing
Source: PLoS One. 2026 Oct 21;21(10):e0361203. doi: 10.1371/journal.pone.0361203 (PMC13686465; doi:10.1371/journal.pone.0361203)
Supplement: Supplementary file 1 [file pone.0361203.s001.zip › Supporting Information/S2 Code.docx]

**S2 Code: Normality Testing and Statistical Analysis**

% Purpose: Perform normality testing on 5-fold cross validation results, and select paired t-test or Wilcoxon signed-rank test based on results

% Applicable to: Black, Cyan, Magenta, Yellow four inks

clear; clc;

%% 1. Load data (using black ink as example)

load('Black_Ink_CV_Results.mat'); % Contains quadratic_R2, lasso_R2, rf_R2, nn_R2, etc.

%% 2. Define statistical test function

% Input: Performance differences between two models (5 values, from 5-fold CV)

% Output: p-value, and the test method used

function [p_value, test_used] = conditional_test(diff_data)

% Shapiro-Wilk normality test

[~, p_norm] = swtest(diff_data); % swtest.m needs to be downloaded

if p_norm > 0.05

% Conforms to normal distribution, use paired t-test

[~, p_value] = ttest(diff_data);

test_used = 'paired t-test';

else

% Does not conform to normal distribution, use Wilcoxon signed-rank test

p_value = signrank(diff_data);

test_used = 'Wilcoxon signed-rank test';

end

end

%% 3. Test R² (RF vs QR)

diff_RF_QR = rf_R2 - quadratic_R2;

[p_RF_QR, test_RF_QR] = conditional_test(diff_RF_QR);

fprintf('RF vs QR (R²): %s, p = %.4f\n', test_RF_QR, p_RF_QR);

%% 4. Test R² (NN vs QR)

diff_NN_QR = nn_R2 - quadratic_R2;

[p_NN_QR, test_NN_QR] = conditional_test(diff_NN_QR);

fprintf('NN vs QR (R²): %s, p = %.4f\n', test_NN_QR, p_NN_QR);

%% 5. Test R² (RF vs NN)

diff_RF_NN = rf_R2 - nn_R2;

[p_RF_NN, test_RF_NN] = conditional_test(diff_RF_NN);

fprintf('RF vs NN (R²): %s, p = %.4f\n', test_RF_NN, p_RF_NN);

%% 6. Perform the same tests for RMSE (code similar, omitted)

% ...

%% 7. Save test results

save('Normality_Test_Results.mat', 'p_*', 'test_*');

fprintf('\nTesting complete, results saved.\n');
