# Supplementary material for: Comparative study of quadratic regression, Lasso, random forest, and multilayer perceptron for modeling optimal solid density in offset printing
Source: PLoS One. 2026 Oct 21;21(10):e0361203. doi: 10.1371/journal.pone.0361203 (PMC13686465; doi:10.1371/journal.pone.0361203)
Supplement: Supplementary file 1 [file pone.0361203.s001.zip › Supporting Information/S2 Table .docx]

**Table S2. Comprehensive statistics of cyan ink model performance and parameter robustness**

| **Category** | **Metric** | **QR** | **Lasso** | **RF** | **MLP** |
| --- | --- | --- | --- | --- | --- |
| **Prediction Performance (5-fold CV)** | Mean R² | 0.7635 | 0.7075 | **0.9836** | 0.9753 |
|  | SD of R² | 0.0500 | 0.0667 | **0.0094** | 0.0271 |
|  | Mean RMSE | 2.9908 | 3.3153 | **0.7604** | 0.8637 |
|  | SD of RMSE | 0.3917 | 0.4041 | 0.2369 | 0.3567 |
| **Optimal Parameters (Full data)** | Optimal X | 1.4337 | 1.6000 | **1.4367** | 1.4481 |
|  | Optimal Y | 16.0598 | 16.0000 | **16.1519** | 16.0000 |
|  | Predicted Max Z | 38.2823 | 45.8292 | 42.9596 | **43.0462** |
| **Parameter Robustness (5-fold CV)** | Optimal X (Mean±Std) | 1.4354±0.0070 | 1.6300±0.0000 | **1.4378±0.0026** | 1.3699±0.1286 |
|  | CV% of X | 0.49% | 0.00% | **0.18%** | 9.39% |
|  | Relative Deviation | 0.12% | 1.87% | **0.08%** | 5.40% |
| **Consistency Check** | QR Optimum on RF (Z') | — | — | **42.25** | — |
|  | QR Optimum on MLP (Z') | — | — | — | 42.65 |
|  | RF Optimum on QR (Z') | 38.24 | — | — | — |
|  | RF Optimum on MLP (Z') | — | — | — | **42.54** |
|  | MLP Optimum on QR (Z') | 37.19 | — | — | — |
|  | MLP Optimum on RF (Z') | — | — | 42.43 | — |

**Note:** Bold values indicate the best performance among the four models.

**Paired t-test results for R² differences (5-fold CV):**

| **Comparison** | **Mean R² Difference** | **p-value** | **Significance** |
| --- | --- | --- | --- |
| RF vs QR | +0.2201 | **0.0004** | Significant |
| MLP vs QR | +0.2118 | **0.0014** | Significant |
| RF vs MLP | +0.0083 | 0.4985 | Not significant |
| Lasso vs QR | -0.0560 | >0.05 | Not significant |

**Key findings for black ink:**

**1. Prediction accuracy:** RF achieved the highest mean R² (0.9836) and lowest RMSE (0.7604), outperforming all other models. MLP also showed good performance (R²=0.9753, RMSE=0.8637).

**2. Parameter stability:** RF exhibited excellent stability with CV of only 0.18% for optimal solid density, compared to QR (0.49%), Lasso (0.00%), and MLP (9.39%). Notably, MLP showed higher variability (CV=9.39%) despite good prediction accuracy.

**3. Statistical significance:** Both RF and MLP significantly outperformed QR (p < 0.01), while no significant difference was found between RF and MLP (p = 0.4985).

**4. Consistency:** The RF optimal point produced consistent predictions when evaluated on other models (42.25 on QR, 42.54 on MLP), confirming the robustness of the broad plateau characteristic.

**5. Recommended model:** RF is recommended for cyan ink with an optimal solid density of **1.44**, achieving a target relative contrast of ≥ 43.0.
