# Supplementary material for: Comparative study of quadratic regression, Lasso, random forest, and multilayer perceptron for modeling optimal solid density in offset printing
Source: PLoS One. 2026 Oct 21;21(10):e0361203. doi: 10.1371/journal.pone.0361203 (PMC13686465; doi:10.1371/journal.pone.0361203)
Supplement: Supplementary file 1 [file pone.0361203.s001.zip › Supporting Information/S3 Code.docx]

## ## 1. Black Ink Hyperparameter Tuning

%% Black Ink Hyperparameter Validation - Determine Optimal Number of Hidden Layer Neurons

% Test [3,4,5,6,8,10] neurons using 5-fold cross validation

clear; clc; close all;

% ==================== Parameter Settings ====================

[params.cv](https://params.cv/)_folds = 5;

params.epochs = 200;

% ==================== Data Preparation ====================

fprintf('=== Black Ink Hyperparameter Validation ===\n');

DATA = [

1.33 15.81 27.8

1.33 15.85 27.6

1.35 15.85 27.7

1.35 15.83 27.8

1.37 15.63 27.5

1.36 15.42 27.2

1.38 15.41 27.1

1.38 15.52 26.8

1.39 15.51 26.7

1.4 15.78 26.7

1.41 15.75 26.5

1.41 15.66 27.1

1.42 15.65 27.1

1.42 15.46 26.8

1.43 15.45 26.9

1.43 16.21 31.1

1.43 16.22 31

1.43 16.17 30.6

1.44 16.16 30.5

1.44 16.39 29.7

1.44 16.38 29.6

1.43 16.23 29.5

1.45 16.22 29.4

1.45 16.11 29.5

1.45 16.12 29.4

1.45 16.46 30.3

1.46 16.47 30.4

1.46 16.5 31.5

1.46 16.52 31.6

1.47 16.53 31.7

1.47 16.53 32.4

1.48 16.54 32.5

1.48 17.12 33.6

1.49 17.11 33.7

1.49 16.52 36.5

1.5 16.51 36.4

1.5 18.69 36.6

1.5 18.68 36.5

1.5 18.72 36.7

1.51 18.71 36.6

1.51 18.73 36.5

1.52 18.72 36.4

1.52 19.04 37.1

1.52 19.03 37

1.53 19.05 37.2

1.53 19.05 36.9

1.53 19.04 36.8

1.54 19.04 36.8

1.54 19.06 37.5

1.55 19.05 37.4

1.55 18.46 38.1

1.56 18.51 38.2

1.56 19.34 38.3

1.57 19.32 38.2

1.57 19.26 38.6

1.58 19.25 38.5

1.58 19.36 38.9

1.59 19.35 38.8

1.59 18.64 38.9

1.59 17.26 39.2

1.6 17.25 39.1

1.6 17.45 39.3

1.61 17.44 39.2

1.61 17.89 39.1

1.61 17.88 39.2

1.62 17.89 39.2

1.62 17.91 39.1

1.62 17.9 39

1.63 17.9 39

1.63 17.43 39.3

1.64 17.42 39.4

1.64 17.42 39.1

1.64 17.43 39.2

1.65 17.42 39.1

1.65 17.41 41.3

1.66 17.4 41.2

1.66 19.11 44.5

1.67 19.1 44.4

1.67 19.6 44.2

1.68 19.5 44.1

1.68 19.14 44.5

1.69 19.13 44.3

1.69 18.11 42.9

1.7 18.12 42.8

1.7 18.44 42.2

1.71 18.43 42.1

1.71 18.47 42.7

1.73 18.45 42.6

1.73 18.52 42.5

1.74 18.51 42.4

1.74 18.91 41.7

1.74 18.9 41.6

1.75 18.9 41.5

1.75 18.93 41.6

1.76 18.94 41.7

1.76 18.98 41.9

1.77 18.99 41.8

1.77 18.97 41.9

1.78 18.96 41.8

1.78 18.97 41.9

1.79 18.96 41.8

1.79 19.74 38.9

1.81 19.73 38.8

1.81 19.99 36.9

1.82 20 37

1.82 20.01 37.1

1.83 20 37.2

1.83 20.02 37

1.84 20.01 37.1

1.84 20.03 36.9

];

X = DATA(:, 1);

Y = DATA(:, 2);

Z = DATA(:, 3);

n = length(X);

fprintf('Sample size: %d\n', n);

fprintf('Solid density range: %.2f - %.2f\n', min(X), max(X));

fprintf('Dot gain range: %.2f - %.2f\n', min(Y), max(Y));

fprintf('Contrast range: %.2f - %.2f\n\n', min(Z), max(Z));

% ==================== Hyperparameter Search ====================

fprintf('Testing different hidden layer neuron counts...\n');

hidden_sizes = [3, 4, 5, 6, 8, 10];

cv_K = [params.cv](https://params.cv/)_folds;

rng(42);

indices_temp = crossvalind('Kfold', n, cv_K);

search_results = zeros(length(hidden_sizes), 2);

for h_idx = 1:length(hidden_sizes)

h = hidden_sizes(h_idx);

fprintf(' Testing hidden neurons=%d (%d/%d)...\n', h, h_idx, length(hidden_sizes));

rmse_folds = zeros(cv_K, 1);

for fold = 1:cv_K

test_idx = (indices_temp == fold);

train_idx = ~test_idx;

X_train = X(train_idx);

Y_train = Y(train_idx);

Z_train = Z(train_idx);

X_test = X(test_idx);

Y_test = Y(test_idx);

Z_test = Z(test_idx);

X_min_tr = min(X_train); X_max_tr = max(X_train);

Y_min_tr = min(Y_train); Y_max_tr = max(Y_train);

Z_min_tr = min(Z_train); Z_max_tr = max(Z_train);

if X_max_tr == X_min_tr; X_max_tr = X_min_tr + eps; end

if Y_max_tr == Y_min_tr; Y_max_tr = Y_min_tr + eps; end

if Z_max_tr == Z_min_tr; Z_max_tr = Z_min_tr + eps; end

X_norm_train = (X_train - X_min_tr) / (X_max_tr - X_min_tr);

Y_norm_train = (Y_train - Y_min_tr) / (Y_max_tr - Y_min_tr);

Z_norm_train = (Z_train - Z_min_tr) / (Z_max_tr - Z_min_tr);

X_norm_test = (X_test - X_min_tr) / (X_max_tr - X_min_tr);

Y_norm_test = (Y_test - Y_min_tr) / (Y_max_tr - Y_min_tr);

rng(42);

net = feedforwardnet(h);

net.trainFcn = 'trainlm';

net.trainParam.epochs = params.epochs;

net.trainParam.showWindow = false;

net.divideFcn = 'dividetrain';

net = train(net, [X_norm_train'; Y_norm_train'], Z_norm_train');

Z_norm_pred = net([X_norm_test'; Y_norm_test']);

Z_pred = Z_norm_pred' * (Z_max_tr - Z_min_tr) + Z_min_tr;

rmse_folds(fold) = sqrt(mean((Z_test - Z_pred).^2));

end

search_results(h_idx, 1) = mean(rmse_folds);

search_results(h_idx, 2) = std(rmse_folds);

fprintf(' Hidden neurons=%d: Mean RMSE=%.4f ± %.4f\n', h, search_results(h_idx, 1), search_results(h_idx, 2));

end

% Output optimal results

[~, best_idx] = min(search_results(:, 1));

best_hidden_black = hidden_sizes(best_idx);

fprintf('\n========== Black Ink Hyperparameter Validation Results ==========\n');

fprintf('Tested hidden layer neuron counts: [3,4,5,6,8,10]\n');

for i = 1:length(hidden_sizes)

fprintf(' %d neurons: RMSE = %.4f ± %.4f\n', hidden_sizes(i), search_results(i,1), search_results(i,2));

end

fprintf('\n★★★★★ Recommendation: Use %d hidden layer neurons for Black Ink ★★★★★\n', best_hidden_black);

fprintf('===================================================================\n');

% Save results

save('Black_Hyperparam_Results.mat', 'hidden_sizes', 'search_results', 'best_hidden_black');

fprintf('\nResults saved to Black_Hyperparam_Results.mat\n');

## ## 2. Cyan Ink Hyperparameter Tuning

%% Cyan Ink Hyperparameter Validation - Determine Optimal Number of Hidden Layer Neurons

% Test [3,4,5,6,8,10] neurons using 5-fold cross validation

clear; clc; close all;

% ==================== Parameter Settings ====================

params.cv_folds = 5;

params.epochs = 200;

% ==================== Data Preparation ====================

fprintf('=== Cyan Ink Hyperparameter Validation ===\n');

DATA = [

1.05 10.12 20.59

1.05 10.12 20.48

1.05 10.12 20.22

1.05 10.12 20.18

1.05 10.12 20.94

1.05 10.12 20.98

1.16 11.23 20.86

1.16 11.23 21.09

1.16 11.23 21.11

1.16 11.23 21.19

1.16 11.23 23.18

1.16 11.23 23.15

1.19 12.17 23.72

1.19 12.17 25.13

1.19 12.17 25.69

1.19 12.17 25.71

1.19 12.17 25.62

1.19 12.17 26.70

1.22 12.85 25.89

1.22 12.85 26.65

1.22 12.85 26.61

1.22 12.85 26.65

1.22 12.85 26.64

1.22 12.85 26.66

1.24 13.25 30.15

1.24 13.25 30.13

1.24 13.25 30.15

1.24 13.25 30.14

1.24 13.25 30.16

1.24 13.25 30.12

1.27 13.88 31.15

1.27 13.88 31.13

1.27 13.88 31.13

1.27 13.88 31.51

1.27 13.88 31.12

1.27 13.88 31.15

1.28 14.32 31.59

1.28 14.32 31.14

1.28 14.32 31.47

1.28 14.32 31.52

1.28 14.32 31.51

1.28 14.32 31.61

1.32 14.62 33.56

1.32 14.62 33.56

1.32 14.62 33.56

1.32 14.62 33.56

1.32 14.62 33.56

1.32 14.62 33.56

1.35 15.14 34.93

1.35 15.14 34.93

1.35 15.14 34.93

1.35 15.14 34.93

1.35 15.14 34.93

1.35 15.14 34.93

1.37 15.31 36.76

1.37 15.31 36.76

1.37 15.31 36.76

1.37 15.31 36.76

1.37 15.31 36.76

1.37 15.31 36.76

1.39 15.75 39.45

1.39 15.75 39.45

1.39 15.75 39.45

1.39 15.75 39.45

1.39 15.75 39.45

1.39 15.75 39.45

1.42 15.95 41.69

1.42 15.95 41.69

1.42 15.95 41.69

1.42 15.95 41.69

1.42 15.95 41.69

1.42 15.95 41.69

1.45 16.35 43.71

1.45 16.35 43.71

1.45 16.35 43.71

1.45 16.35 43.71

1.45 16.35 43.71

1.45 16.35 43.71

1.46 16.54 40.83

1.46 16.54 40.83

1.46 16.54 40.83

1.46 16.54 40.83

1.46 16.54 40.83

1.46 16.54 40.83

1.49 17.08 37.18

1.49 17.08 37.18

1.49 17.08 37.18

1.49 17.08 37.18

1.49 17.08 37.18

1.49 17.08 37.18

1.52 17.58 34.36

1.52 17.58 34.36

1.52 17.58 34.36

1.52 17.58 34.36

1.52 17.58 34.36

1.52 17.58 34.36

1.54 18.13 32.77

1.54 18.13 32.77

1.54 18.13 32.77

1.54 18.13 32.77

1.54 18.13 32.77

1.54 18.13 32.77

1.57 18.75 30.39

1.57 18.75 30.39

1.57 18.75 30.39

1.57 18.75 30.39

1.63 18.75 27.83

1.63 18.75 27.83

1.63 19.32 27.83

1.63 19.32 27.83

];

X = DATA(:, 1);

Y = DATA(:, 2);

Z = DATA(:, 3);

n = length(X);

fprintf('Sample size: %d\n', n);

fprintf('Solid density range: %.2f - %.2f\n', min(X), max(X));

fprintf('Dot gain range: %.2f - %.2f\n', min(Y), max(Y));

fprintf('Contrast range: %.2f - %.2f\n\n', min(Z), max(Z));

% ==================== Hyperparameter Search ====================

fprintf('Testing different hidden layer neuron counts...\n');

hidden_sizes = [3, 4, 5, 6, 8, 10];

cv_K = [params.cv](https://params.cv/)_folds;

rng(42);

indices_temp = crossvalind('Kfold', n, cv_K);

search_results = zeros(length(hidden_sizes), 2);

for h_idx = 1:length(hidden_sizes)

h = hidden_sizes(h_idx);

fprintf(' Testing hidden neurons=%d (%d/%d)...\n', h, h_idx, length(hidden_sizes));

rmse_folds = zeros(cv_K, 1);

for fold = 1:cv_K

test_idx = (indices_temp == fold);

train_idx = ~test_idx;

X_train = X(train_idx);

Y_train = Y(train_idx);

Z_train = Z(train_idx);

X_test = X(test_idx);

Y_test = Y(test_idx);

Z_test = Z(test_idx);

X_min_tr = min(X_train); X_max_tr = max(X_train);

Y_min_tr = min(Y_train); Y_max_tr = max(Y_train);

Z_min_tr = min(Z_train); Z_max_tr = max(Z_train);

if X_max_tr == X_min_tr; X_max_tr = X_min_tr + eps; end

if Y_max_tr == Y_min_tr; Y_max_tr = Y_min_tr + eps; end

if Z_max_tr == Z_min_tr; Z_max_tr = Z_min_tr + eps; end

X_norm_train = (X_train - X_min_tr) / (X_max_tr - X_min_tr);

Y_norm_train = (Y_train - Y_min_tr) / (Y_max_tr - Y_min_tr);

Z_norm_train = (Z_train - Z_min_tr) / (Z_max_tr - Z_min_tr);

X_norm_test = (X_test - X_min_tr) / (X_max_tr - X_min_tr);

Y_norm_test = (Y_test - Y_min_tr) / (Y_max_tr - Y_min_tr);

rng(42);

net = feedforwardnet(h);

net.trainFcn = 'trainlm';

net.trainParam.epochs = params.epochs;

net.trainParam.showWindow = false;

net.divideFcn = 'dividetrain';

net = train(net, [X_norm_train'; Y_norm_train'], Z_norm_train');

Z_norm_pred = net([X_norm_test'; Y_norm_test']);

Z_pred = Z_norm_pred' * (Z_max_tr - Z_min_tr) + Z_min_tr;

rmse_folds(fold) = sqrt(mean((Z_test - Z_pred).^2));

end

search_results(h_idx, 1) = mean(rmse_folds);

search_results(h_idx, 2) = std(rmse_folds);

fprintf(' Hidden neurons=%d: Mean RMSE=%.4f ± %.4f\n', h, search_results(h_idx, 1), search_results(h_idx, 2));

end

% Output optimal results

[~, best_idx] = min(search_results(:, 1));

best_hidden_cyan = hidden_sizes(best_idx);

fprintf('\n========== Cyan Ink Hyperparameter Validation Results ==========\n');

fprintf('Tested hidden layer neuron counts: [3,4,5,6,8,10]\n');

for i = 1:length(hidden_sizes)

fprintf(' %d neurons: RMSE = %.4f ± %.4f\n', hidden_sizes(i), search_results(i,1), search_results(i,2));

end

fprintf('\n★★★★★ Recommendation: Use %d hidden layer neurons for Cyan Ink ★★★★★\n', best_hidden_cyan);

fprintf('===================================================================\n');

% Save results

save('Cyan_Hyperparam_Results.mat', 'hidden_sizes', 'search_results', 'best_hidden_cyan');

fprintf('\nResults saved to Cyan_Hyperparam_Results.mat\n');

## ## 3. Magenta Ink Hyperparameter Tuning

%% Magenta Ink Hyperparameter Validation - Determine Optimal Number of Hidden Layer Neurons

% Test [3,4,5,6,8,10] neurons using 5-fold cross validation

clear; clc; close all;

% ==================== Parameter Settings ====================

params.cv_folds = 5;

params.epochs = 200;

% ==================== Data Preparation ====================

fprintf('=== Magenta Ink Hyperparameter Validation ===\n');

DATA = [

0.85 11.65 28.71

0.85 11.64 28.73

0.86 11.64 28.72

0.86 11.62 28.76

0.87 11.68 29.81

0.87 11.71 31.95

0.88 11.77 31.92

0.89 11.79 31.97

0.9 11.77 31.93

0.91 11.76 31.99

0.92 11.78 32.08

0.93 11.79 32.07

0.94 11.81 32.06

0.95 11.86 32.07

0.97 11.81 32.03

0.99 11.84 32.01

1 11.81 33.85

1.12 11.89 34.05

1.15 12.08 34.91

1.16 12.34 35.11

1.17 12.78 35.19

1.19 12.84 35.27

1.2 12.81 35.61

1.22 12.8 35.97

1.25 12.79 36.02

1.28 12.82 37.53

1.29 12.81 37.2

1.3 14.37 38.72

1.31 14.38 38.71

1.32 12.46 38.82

1.32 14.34 38.75

1.33 12.45 38.99

1.33 12.47 39.19

1.33 14.38 39.18

1.34 12.44 39.94

1.34 13.51 40.12

1.34 13.52 40.15

1.34 14.39 40.14

1.35 13.5 40.65

1.35 13.52 40.64

1.35 14.03 40.66

1.36 12.48 41.06

1.36 13.51 41.18

1.36 14.08 41.16

1.37 14.07 42.24

1.37 14.05 42.26

1.37 14.62 42.25

1.38 14.09 43.11

1.38 14.61 43.19

1.39 14.6 43.68

1.4 14.61 44.99

1.41 14.63 45.23

1.41 15.16 45.2

1.41 15.47 45.2

1.42 15.18 44.82

1.42 15.17 44.83

1.42 15.48 44.81

1.43 15.19 44.52

1.43 15.49 44.42

1.43 15.48 44.41

1.43 15.66 44.56

1.44 15.17 44.24

1.44 15.67 44.22

1.45 15.67 44.12

1.45 16.19 44.14

1.46 16.18 44.15

1.46 16.16 44.12

1.47 16.18 44.05

1.47 16.52 44.04

1.48 16.53 44.05

1.48 16.53 44.03

1.49 16.97 44.04

1.49 16.96 44.06

1.5 16.97 44.06

1.5 16.98 44.04

1.5 17.31 44.07

1.51 17.32 43.65

1.52 17.33 43.65

1.53 17.33 42.32

1.53 17.35 42.38

1.54 17.63 41.72

1.55 17.66 41.46

1.55 17.67 41.15

1.56 17.65 41.05

1.57 17.64 40.65

1.6 18.07 40.38

1.6 18.06 40.36

1.61 18.06 40.16

1.61 18.07 39.95

1.62 18.08 39.39

1.62 22.92 39.34

1.63 18.53 39.14

1.63 22.93 39.12

1.64 18.52 38.48

1.64 18.54 38.42

1.64 22.94 38.42

1.64 22.94 38.43

1.65 18.53 37.71

1.65 18.52 37.11

1.65 22.95 37.74

1.65 24.65 37.62

1.65 24.68 37.64

1.66 24.67 37.13

1.66 24.67 37.15

1.66 25.36 37.23

1.67 24.66 36.62

1.67 25.37 36.55

1.68 25.37 35.21

1.68 25.35 35.24

1.69 25.38 34.23

];

X = DATA(:, 1);

Y = DATA(:, 2);

Z = DATA(:, 3);

n = length(X);

fprintf('Sample size: %d\n', n);

fprintf('Solid density range: %.2f - %.2f\n', min(X), max(X));

fprintf('Dot gain range: %.2f - %.2f\n', min(Y), max(Y));

fprintf('Contrast range: %.2f - %.2f\n\n', min(Z), max(Z));

% ==================== Hyperparameter Search ====================

fprintf('Testing different hidden layer neuron counts...\n');

hidden_sizes = [3, 4, 5, 6, 8, 10];

cv_K = [params.cv](https://params.cv/)_folds;

rng(42);

indices_temp = crossvalind('Kfold', n, cv_K);

search_results = zeros(length(hidden_sizes), 2);

for h_idx = 1:length(hidden_sizes)

h = hidden_sizes(h_idx);

fprintf(' Testing hidden neurons=%d (%d/%d)...\n', h, h_idx, length(hidden_sizes));

rmse_folds = zeros(cv_K, 1);

for fold = 1:cv_K

test_idx = (indices_temp == fold);

train_idx = ~test_idx;

X_train = X(train_idx);

Y_train = Y(train_idx);

Z_train = Z(train_idx);

X_test = X(test_idx);

Y_test = Y(test_idx);

Z_test = Z(test_idx);

X_min_tr = min(X_train); X_max_tr = max(X_train);

Y_min_tr = min(Y_train); Y_max_tr = max(Y_train);

Z_min_tr = min(Z_train); Z_max_tr = max(Z_train);

if X_max_tr == X_min_tr; X_max_tr = X_min_tr + eps; end

if Y_max_tr == Y_min_tr; Y_max_tr = Y_min_tr + eps; end

if Z_max_tr == Z_min_tr; Z_max_tr = Z_min_tr + eps; end

X_norm_train = (X_train - X_min_tr) / (X_max_tr - X_min_tr);

Y_norm_train = (Y_train - Y_min_tr) / (Y_max_tr - Y_min_tr);

Z_norm_train = (Z_train - Z_min_tr) / (Z_max_tr - Z_min_tr);

X_norm_test = (X_test - X_min_tr) / (X_max_tr - X_min_tr);

Y_norm_test = (Y_test - Y_min_tr) / (Y_max_tr - Y_min_tr);

rng(42);

net = feedforwardnet(h);

net.trainFcn = 'trainlm';

net.trainParam.epochs = params.epochs;

net.trainParam.showWindow = false;

net.divideFcn = 'dividetrain';

net = train(net, [X_norm_train'; Y_norm_train'], Z_norm_train');

Z_norm_pred = net([X_norm_test'; Y_norm_test']);

Z_pred = Z_norm_pred' * (Z_max_tr - Z_min_tr) + Z_min_tr;

rmse_folds(fold) = sqrt(mean((Z_test - Z_pred).^2));

end

search_results(h_idx, 1) = mean(rmse_folds);

search_results(h_idx, 2) = std(rmse_folds);

fprintf(' Hidden neurons=%d: Mean RMSE=%.4f ± %.4f\n', h, search_results(h_idx, 1), search_results(h_idx, 2));

end

% Output optimal results

[~, best_idx] = min(search_results(:, 1));

best_hidden_magenta = hidden_sizes(best_idx);

fprintf('\n========== Magenta Ink Hyperparameter Validation Results ==========\n');

fprintf('Tested hidden layer neuron counts: [3,4,5,6,8,10]\n');

for i = 1:length(hidden_sizes)

fprintf(' %d neurons: RMSE = %.4f ± %.4f\n', hidden_sizes(i), search_results(i,1), search_results(i,2));

end

fprintf('\n★★★★★ Recommendation: Use %d hidden layer neurons for Magenta Ink ★★★★★\n', best_hidden_magenta);

fprintf('========================================================================\n');

% Save results

save('Magenta_Hyperparam_Results.mat', 'hidden_sizes', 'search_results', 'best_hidden_magenta');

fprintf('\nResults saved to Magenta_Hyperparam_Results.mat\n');

## ## 4. Yellow Ink Hyperparameter Tuning

%% Yellow Ink Hyperparameter Validation - Determine Optimal Number of Hidden Layer Neurons

% Test [3,4,5,6,8,10] neurons using 5-fold cross validation

clear; clc; close all;

% ==================== Parameter Settings ====================

params.cv_folds = 5;

params.epochs = 200;

% ==================== Data Preparation ====================

fprintf('=== Yellow Ink Hyperparameter Validation ===\n');

DATA = [

0.85 10.01 18.73

0.85 10.03 18.76

0.85 10.02 18.79

0.86 10.04 18.83

0.86 10.01 18.79

0.86 10.03 18.81

0.87 10.04 18.85

0.87 10.05 18.84

0.87 10.05 18.92

0.88 10.03 19.47

0.88 10.05 19.45

0.88 10.07 19.65

0.89 11.68 20.83

0.89 11.99 21.43

0.89 12.09 21.63

0.90 13.35 22.82

0.90 15.60 23.31

0.90 15.61 23.80

0.91 10.10 23.84

0.91 13.33 24.84

0.91 14.13 23.83

0.91 14.14 23.81

0.91 15.61 23.82

0.91 15.63 23.81

0.92 10.12 25.81

0.92 10.12 25.21

0.92 13.34 25.91

0.92 13.36 25.82

0.92 14.13 26.69

0.92 14.12 26.82

0.92 15.62 27.84

0.92 15.62 27.82

0.93 11.01 28.59

0.93 11.02 28.65

0.93 10.13 28.78

0.93 13.35 28.89

0.93 14.11 29.49

0.94 11.02 29.87

0.94 14.12 30.14

0.94 14.31 30.54

0.94 16.22 29.96

0.95 11.04 31.65

0.95 11.03 31.84

0.95 14.30 32.03

0.95 14.30 32.04

0.95 14.33 32.03

0.95 15.26 32.02

0.95 15.27 32.02

0.95 16.22 32.04

0.96 11.03 32.13

0.96 14.32 32.15

0.96 15.28 32.13

0.96 16.24 32.14

0.97 14.35 32.21

0.97 15.26 32.23

0.97 16.23 32.22

0.97 17.80 32.20

0.98 15.29 32.33

0.98 17.81 32.34

0.98 17.77 32.33

0.98 16.41 32.34

0.99 17.79 32.98

0.99 17.78 33.04

0.99 16.34 33.03

1.00 17.91 33.11

1.00 16.39 33.10

1.00 16.36 33.04

1.01 12.26 33.10

1.01 12.25 33.13

1.01 16.37 33.12

1.02 12.26 33.16

1.02 12.27 33.18

1.02 16.35 33.19

1.03 12.27 33.79

1.03 16.61 33.81

1.03 17.37 33.82

1.04 12.28 31.80

1.04 13.12 29.65

1.04 16.65 30.89

1.04 18.57 30.47

1.04 17.38 30.67

1.04 17.35 30.53

1.05 13.12 29.67

1.05 13.11 28.94

1.05 17.37 28.87

1.05 17.36 29.59

1.06 13.10 29.68

1.06 13.13 28.46

1.06 16.61 27.81

1.07 16.69 26.99

1.07 18.56 26.58

1.08 18.59 26.53

1.08 17.10 25.99

1.09 14.25 24.84

1.09 17.11 24.99

1.09 19.02 24.72

1.10 14.26 23.41

1.10 14.26 23.37

1.10 17.12 22.32

1.11 15.23 22.26

1.11 14.27 21.44

1.11 14.27 21.42

1.12 15.26 22.27

1.12 15.26 22.24

1.12 14.26 21.43

1.13 19.04 20.85

1.13 15.27 22.28

1.14 16.01 20.86

1.15 15.24 22.23

1.15 15.21 20.74

];

X = DATA(:, 1);

Y = DATA(:, 2);

Z = DATA(:, 3);

n = length(X);

fprintf('Sample size: %d\n', n);

fprintf('Solid density range: %.2f - %.2f\n', min(X), max(X));

fprintf('Dot gain range: %.2f - %.2f\n', min(Y), max(Y));

fprintf('Contrast range: %.2f - %.2f\n\n', min(Z), max(Z));

% ==================== Hyperparameter Search ====================

fprintf('Testing different hidden layer neuron counts...\n');

hidden_sizes = [3, 4, 5, 6, 8, 10];

cv_K = [params.cv](https://params.cv/)_folds;

rng(42);

indices_temp = crossvalind('Kfold', n, cv_K);

search_results = zeros(length(hidden_sizes), 2);

for h_idx = 1:length(hidden_sizes)

h = hidden_sizes(h_idx);

fprintf(' Testing hidden neurons=%d (%d/%d)...\n', h, h_idx, length(hidden_sizes));

rmse_folds = zeros(cv_K, 1);

for fold = 1:cv_K

test_idx = (indices_temp == fold);

train_idx = ~test_idx;

X_train = X(train_idx);

Y_train = Y(train_idx);

Z_train = Z(train_idx);

X_test = X(test_idx);

Y_test = Y(test_idx);

Z_test = Z(test_idx);

X_min_tr = min(X_train); X_max_tr = max(X_train);

Y_min_tr = min(Y_train); Y_max_tr = max(Y_train);

Z_min_tr = min(Z_train); Z_max_tr = max(Z_train);

if X_max_tr == X_min_tr; X_max_tr = X_min_tr + eps; end

if Y_max_tr == Y_min_tr; Y_max_tr = Y_min_tr + eps; end

if Z_max_tr == Z_min_tr; Z_max_tr = Z_min_tr + eps; end

X_norm_train = (X_train - X_min_tr) / (X_max_tr - X_min_tr);

Y_norm_train = (Y_train - Y_min_tr) / (Y_max_tr - Y_min_tr);

Z_norm_train = (Z_train - Z_min_tr) / (Z_max_tr - Z_min_tr);

X_norm_test = (X_test - X_min_tr) / (X_max_tr - X_min_tr);

Y_norm_test = (Y_test - Y_min_tr) / (Y_max_tr - Y_min_tr);

rng(42);

net = feedforwardnet(h);

net.trainFcn = 'trainlm';

net.trainParam.epochs = params.epochs;

net.trainParam.showWindow = false;

net.divideFcn = 'dividetrain';

net = train(net, [X_norm_train'; Y_norm_train'], Z_norm_train');

Z_norm_pred = net([X_norm_test'; Y_norm_test']);

Z_pred = Z_norm_pred' * (Z_max_tr - Z_min_tr) + Z_min_tr;

rmse_folds(fold) = sqrt(mean((Z_test - Z_pred).^2));

end

search_results(h_idx, 1) = mean(rmse_folds);

search_results(h_idx, 2) = std(rmse_folds);

fprintf(' Hidden neurons=%d: Mean RMSE=%.4f ± %.4f\n', h, search_results(h_idx, 1), search_results(h_idx, 2));

end

% Output optimal results

[~, best_idx] = min(search_results(:, 1));

best_hidden_yellow = hidden_sizes(best_idx);

fprintf('\n========== Yellow Ink Hyperparameter Validation Results ==========\n');

fprintf('Tested hidden layer neuron counts: [3,4,5,6,8,10]\n');

for i = 1:length(hidden_sizes)

fprintf(' %d neurons: RMSE = %.4f ± %.4f\n', hidden_sizes(i), search_results(i,1), search_results(i,2));

end

fprintf('\n★★★★★ Recommendation: Use %d hidden layer neurons for Yellow Ink ★★★★★\n', best_hidden_yellow);

fprintf('====================================================================\n');

% Save results

save('Yellow_Hyperparam_Results.mat', 'hidden_sizes', 'search_results', 'best_hidden_yellow');

fprintf('\nResults saved to Yellow_Hyperparam_Results.mat\n');
