# Supplementary material for: Comparative study of quadratic regression, Lasso, random forest, and multilayer perceptron for modeling optimal solid density in offset printing
Source: PLoS One. 2026 Oct 21;21(10):e0361203. doi: 10.1371/journal.pone.0361203 (PMC13686465; doi:10.1371/journal.pone.0361203)
Supplement: Supplementary file 1 [file pone.0361203.s001.zip › Supporting Information/S3 Table.docx]

**Table S3. Comprehensive statistics of magenta ink model performance and parameter robustness**

| **Category** | **Metric** | **QR** | **Lasso** | **RF** | **MLP** |
| --- | --- | --- | --- | --- | --- |
| **Prediction Performance (5-fold CV)** | Mean R² | 0.9078 | 0.8852 | 0.9702 | **0.9778** |
|  | SD of R² | 0.0474 | 0.0507 | 0.0084 | 0.0128 |
|  | Mean RMSE | 1.2543 | 1.4157 | 0.7351 | **0.6039** |
|  | SD of RMSE | 0.0996 | 0.1465 | 0.1124 | 0.0927 |
| **Optimal Parameters (Full data)** | Optimal X | 1.4814 | 1.3000 | **1.4165** | 1.4671 |
|  | Optimal Y | 15.4934 | 26.0000 | **15.3671** | 14.0000 |
|  | Predicted Max Z | 43.3202 | 63.4824 | 44.7679 | **45.7903** |
| **Parameter Robustness (5-fold CV)** | Optimal X (Mean±Std) | 1.4813±0.0020 | 0.8500±0.0000 | **1.4205±0.0045** | 1.4718±0.1666 |
|  | CV% of X | 0.13% | 0.00% | **0.31%** | 11.32% |
|  | Relative Deviation | 0.00% | 34.62% | **0.28%** | 0.32% |
| **Consistency Check** | QR Optimum on RF (Z') | — | — | **44.31** | — |
|  | QR Optimum on MLP (Z') | — | — | — | 44.52 |
|  | RF Optimum on QR (Z') | 43.45 | — | — | — |
|  | RF Optimum on MLP (Z') | — | — | — | **44.33** |
|  | MLP Optimum on QR (Z') | 43.49 | — | — | — |
|  | MLP Optimum on RF (Z') | — | — | 42.65 | — |

**Note:** Bold values indicate the best performance among the four models.

**Paired t-test results for R² differences (5-fold CV):**

| **Comparison** | **Mean R² Difference** | **p-value** | **Significance** |
| --- | --- | --- | --- |
| MLP vs QR | +0.0700 | **0.0127** | Significant |
| RF vs QR | +0.0625 | **0.0342** | Significant |
| RF vs MLP | -0.0076 | 0.2592 | Not significant |
| Lasso vs QR | -0.0225 | >0.05 | Not significant |

**Key findings for black ink:**

**1. Prediction accuracy:** MLP achieved the highest mean R² (0.9778) and lowest RMSE (0.6039), followed closely by RF (R²=0.9702, RMSE=0.7351). Both non-parametric methods significantly outperformed QR and Lasso.

**2. Parameter stability:** RF exhibited excellent stability with CV of only 0.31% for optimal solid density, while MLP showed substantially higher variability (CV=11.32%). QR also showed good stability (CV=0.13%), but with lower prediction accuracy.

**3. Statistical significance:** Both MLP and RF significantly outperformed QR (p < 0.05), while no significant difference was found between RF and MLP (p = 0.2592).

**4. Consistency:** The RF optimal point produced consistent predictions when evaluated on other models (44.31 on QR, 44.33 on MLP), confirming the robustness of the broad plateau characteristic.

**5. Recommended model:** Although MLP achieved slightly higher prediction accuracy, the difference from RF was not statistically significant. Given RF's superior stability (CV=0.31% vs. MLP's 11.32%), **RF is recommended for magenta ink** with an optimal solid density of **1.42**, achieving a target relative contrast of ≥ 44.7.
