# Supplementary material for: Comparative study of quadratic regression, Lasso, random forest, and multilayer perceptron for modeling optimal solid density in offset printing
Source: PLoS One. 2026 Oct 21;21(10):e0361203. doi: 10.1371/journal.pone.0361203 (PMC13686465; doi:10.1371/journal.pone.0361203)
Supplement: Supplementary file 1 [file pone.0361203.s001.zip › Supporting Information/S4 Table.docx]

**Table S4. Comprehensive statistics of yellow ink model performance and parameter robustness**

| **Category** | **Metric** | **QR** | **Lasso** | **RF** | **MLP** |
| --- | --- | --- | --- | --- | --- |
| **Prediction Performance (5-fold CV)** | Mean R² | 0.8577 | 0.8532 | 0.9377 | **0.9770** |
|  | SD of R² | 0.0423 | 0.0514 | 0.0420 | **0.0155** |
|  | Mean RMSE | 1.7887 | 1.8070 | 1.1369 | **0.6904** |
|  | SD of RMSE | 0.2237 | 0.2038 | 0.2341 | **0.1425** |
| **Optimal Parameters (Full data)** | Optimal X | 1.0011 | 0.9981 | 1.0133 | **0.9905** |
|  | Optimal Y | 15.0113 | 20.0000 | 16.4557 | **10.6031** |
|  | Predicted Max Z | 32.0361 | 32.0495 | 32.9321 | **33.6639** |
| **Parameter Robustness (5-fold CV)** | Optimal X (Mean±Std) | 1.0018±0.0021 | 1.0027±0.0070 | 1.0009±0.0054 | **0.9912±0.0017** |
|  | CV% of X | 0.21% | 0.70% | 0.54% | **0.17%** |
|  | Relative Deviation | 0.06% | 0.46% | 1.22% | **0.07%** |
| **Consistency Check** | Hidden Neurons | — | — | — | **3** |
|  | QR Optimum on RF (Z') | — | — | 31.35 | — |
|  | QR Optimum on MLP (Z') | — | — | — | **33.24** |
|  | RF Optimum on QR (Z') | 31.94 | — | — | — |
|  | RF Optimum on MLP (Z') | — | — | — | **32.81** |
|  | MLP Optimum on QR (Z') | *Out of range* | — | — | — |
|  | MLP Optimum on RF (Z') | — | — | *Out of range* | — |

**Note:** Bold values indicate the best performance among the four models.

**Paired t-test results for R² differences (5-fold CV):**

| **Comparison** | **Mean R² Difference** | **p-value** | **Significance** |
| --- | --- | --- | --- |
| MLP vs QR | +0.1193 | **0.0008** | Significant |
| RF vs QR | +0.0800 | **0.0011** | Significant |
| RF vs MLP | -0.0393 | **0.0296** | **Significant** |
| Lasso vs QR | -0.0045 | >0.05 | Not significant |

**Key findings for black ink:**

**1. Prediction accuracy:** MLP with 3 hidden neurons achieved the highest mean R² (0.9770) and lowest RMSE (0.6904), significantly outperforming all other models.

**2. Parameter stability:** MLP exhibited excellent stability with CV of only 0.17% for optimal solid density, the lowest among all four models. QR also showed good stability (CV=0.21%), but with much lower prediction accuracy.

**3. Statistical significance:** MLP significantly outperformed QR (p=0.0008), RF (p=0.0296), and Lasso. RF also significantly outperformed QR (p=0.0011). This is the only ink color where MLP showed statistically significant superiority over RF.

**4. MLP architecture:** Based on systematic hyperparameter search over [3,4,5,6,8,10] hidden neurons, the 3-neuron MLP was adopted. The 5-neuron MLP exhibited training instability (RMSE range: 0.67-2.58).

**5. Consistency:** The MLP optimal point (X=0.9905, Y=10.0000) lies near the lower boundary of the dot gain range, causing out-of-range predictions when evaluated on other models. The RF optimal point produced consistent predictions on MLP (32.81), confirming reliability.

**6. Recommended model:** MLP with 3 hidden neurons is recommended for yellow ink with an optimal solid density of **0.99**, achieving a target relative contrast of ≥ 32.9.
